# Synthesis and Evaluation of Boron-Containing Heterocyclic Compounds with Antimicrobial and Anticancer Activities

**DOI:** 10.3390/molecules30051117

**Published:** 2025-02-28

**Authors:** João Lucas Bruno Prates, Samanta de Matos Silva, Kaila Petrolina Medina-Alarcón, Kelvin Sousa dos Santos, Jenyffie Araujo Belizario, Juliana Romano Lopes, Freddy Humberto Marin-Dett, Debora Leite Campos, Maria José Soares Mendes Giannini, Ana Marisa Fusco-Almeida, Paula Aboud Barbugli, Fernando Rogério Pavan, Jean Leandro Dos Santos

**Affiliations:** 1 School of Pharmaceutical Sciences, São Paulo State University (UNESP), Araraquara 14800-903, SP, Brazil; samanta.matos@unesp.br (S.d.M.S.); kaila.medina@unesp.br (K.P.M.-A.); k.santos@unesp.br (K.S.d.S.); jenyffie.belizario@unesp.br (J.A.B.); romano.lopes@unesp.br (J.R.L.); freddy.m.dett@unesp.br (F.H.M.-D.); leite.debora26@gmail.com (D.L.C.); maria.giannini@unesp.br (M.J.S.M.G.); ana.marisa@unesp.br (A.M.F.-A.); paula.barbugli@unesp.br (P.A.B.); fernando.pavan@unesp.br (F.R.P.); 2 Institute of Chemistry, São Paulo State University (UNESP), Araraquara 14800-900, SP, Brazil; 3 School of Dentistry, São Paulo State University (UNESP), Araraquara 14801-385, SP, Brazil

**Keywords:** organoboron, boronic acid, benzoxaborole, boron-containing compounds, *Mycobacterium tuberculosis*, antimicrobial, fungi, cancer

## Abstract

Organoboron compounds, especially those containing boronic acid and benzoxaborole in their structure, have been gaining prominence in medicinal chemistry, following the FDA approval of tavaborole for the treatment of onychomycosis and bortezomib for multiple myeloma. The antimicrobial and anticancer effects of organoboron compounds motivate the investigation of the effects of the novel derivatives described here. A total of fourteen new boronic derivatives were synthesized and characterized using analytical methods. The antimicrobial activities were evaluated against *M. tuberculosis* (*Mtb*) H37Rv strains and fungal dermatophytes (*C. albicans*, ATCC 90028; *T. rubrum*, ATCC 28189; and *T. mentagrophytes*, ATCC 11481), while the anticancer effect was evaluated against oral squamous cell carcinoma (SCC) cell lines. Several promising boron-containing prototypes were identified, providing a foundation for further molecular optimization in the development of new antimicrobial and anticancer compounds.

## 1. Introduction

**Figure 1:**
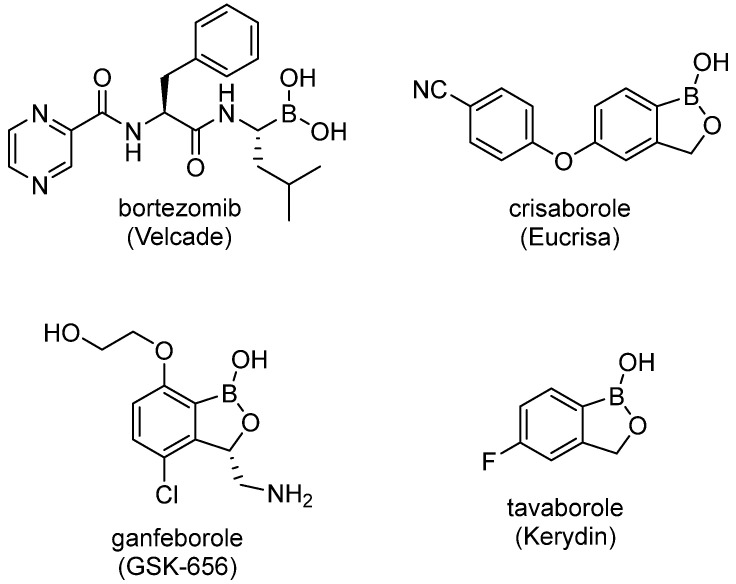
Chemical structure of boronic acid, bortezomibe (Velcade^®^), crisaborole (Eucrisa^®^) ganfeborole (GSK-656), and tavaborole (Kerydin^®^).

Organic molecules containing boron in their structure have garnered significant attention in chemistry due to their diverse applications, in both synthetic and biological contexts [1,2]. Boronic acid derivatives possess the ability to form reversible covalent bonds with nucleophilic amino acid residues [3,4,5,6], enabling their use in drug design for a range of therapeutic applications, including anticancer, anti-inflammatory, antibacterial, and antifungal activities (Figure 1) [7,8,9,10].

The therapeutic potential of boronic acid and its derivatives was first acknowledged in the mid-2000s, when the Food and Drug Administration (FDA) approved bortezomib (Velcade^®^) in 2003, establishing it as the first drug of its class (Figure 1). Bortezomib, an anticancer drug utilized in the treatment of multiple myeloma and mantle cell lymphoma, works by reversibly inhibiting the 26S proteasome, a protein complex responsible for degrading ubiquitinated proteins. This inhibition is achieved through the covalent binding of its boron atom to the threonine residue (Thr1) at the catalytic site of the proteasome, demonstrating a notable affinity with a Ki of 0.62 nM [11,12].

Crisaborole (Eucrisa^®^) (Figure 1), approved by the FDA in 2016 for the treatment of atopic dermatitis, serves as a noteworthy example of a boron-containing anti-inflammatory drug that emerged as an alternative to topical calcineurin inhibitors and corticosteroids. Acting as a reversible and competitive inhibitor, the boron atom in crisaborole binds to the zinc and magnesium ions at the active site of phosphodiesterase 4 (PDE4), leading to increased intracellular levels of cyclic adenosine monophosphate (cAMP) and ultimately reducing pro-inflammatory cytokine production [13].

In the pursuit of new therapeutic agents against tuberculosis, researchers developed the compound (*S*)-3-(aminomethyl)-4-chloro-7-(2-hydroxyethoxy)benzo[*c*][1,2]oxaborol-1(*3H*)-ol, commonly known as GSK-3036656 (ganfeborole) (Figure 1). This benzoxaborole derivative acts as an inhibitor of leucyl-tRNA synthetase (LeuRS) in *Mycobacterium tuberculosis*, disrupting the sequential transport of amino acids by forming a covalent bond between the boron atom in its structure and the adenosine monophosphate (AMP) Ade76 of tRNALeu. By inhibiting protein synthesis, a vital process for the survival of *M. tuberculosis*, this interaction effectively disrupts mycobacterial function. In vitro assays showed that ganfeborole exhibited an IC_50_ of 0.20 µM for LeuRS and an MIC of 0.08 µM against *M. tuberculosis* H37Rv strains. Currently, it is being evaluated in a phase 2a open-label randomized clinical trial [14,15].

Tavaborole, also known as AN2690, received FDA approval in 2014 for the treatment of onychomycosis and is commercially available under the trade name Kerydin^®^ (Figure 1). This antifungal agent exerts its effect by inhibiting tRNA synthetase, a mechanism of action comparable to that of GSK-656. By targeting tRNA synthetase, tavaborole disrupts protein synthesis in the fungus, ultimately compromising its viability [16,17]. Tavaborole exhibits over 1000-fold selectivity for fungal leucyl-tRNA synthetase compared to its mammalian counterpart. Furthermore, it demonstrates potent antifungal activity, with MIC values of 2.0 µg/mL against dermatophyte strains, such as *T. rubrum* (ATCC 1890, ATCC 18759), *T. mentagrophytes* (ATCC 28185), *M. audouinii* (ATCC 42558), and *M. gypseum* (ATCC 24103), and 1.0 µg/mL against *E. floccosum* (ATCC 525066) [18].

**Figure 2:**
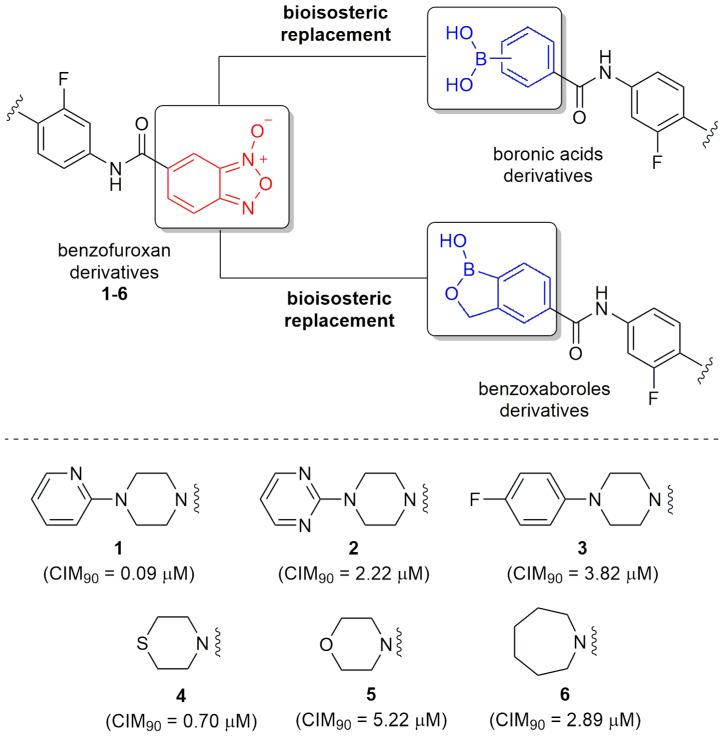
Design of boronic acid and benzoxaborole derivatives.

In a constant effort to search for new antimicrobial and anticancer agents, our laboratory has carried out molecular optimization focusing on boron-containing derivatives. Previously, we synthesized and evaluated a series of benzofuroxan derivatives (1–6) with promising anti-*Mycobacterium tuberculosis* with MIC_90_ values ranging from 0.09 to 19.20 µM, including multi-drug-resistant clinical isolates (MDR-TB) (Figure 2) [19]. Preliminary studies on the mode of action suggested that protein synthesis inhibition could be a potential mechanism [19]. Therefore, by exploring the bioisosteric replacement of benzofuroxan with benzoxaboroles and boronic acids, we synthesized and evaluated a total of fourteen novel compounds (Figure 2). Moreover, we explored their antifungal activity against dermatophytes and *Candida albicans*, drawing inspiration from the well-established antifungal properties of tavaborole.

Furthermore, this screening was enhanced by a comprehensive evaluation of its anticancer potential. Although the anticancer effects of boron-containing compounds have been reported in the literature [7,20], there is limited information regarding their effects on head and neck cancer. Thus, to better understand their potential application, we further evaluated their activity against SCC-25 and NOK-si cell lines.

## 2. Results

### 2.1. Chemistry

#### 2.1.1. Synthesis of Boronic Acid Derivatives

**Scheme 1:**
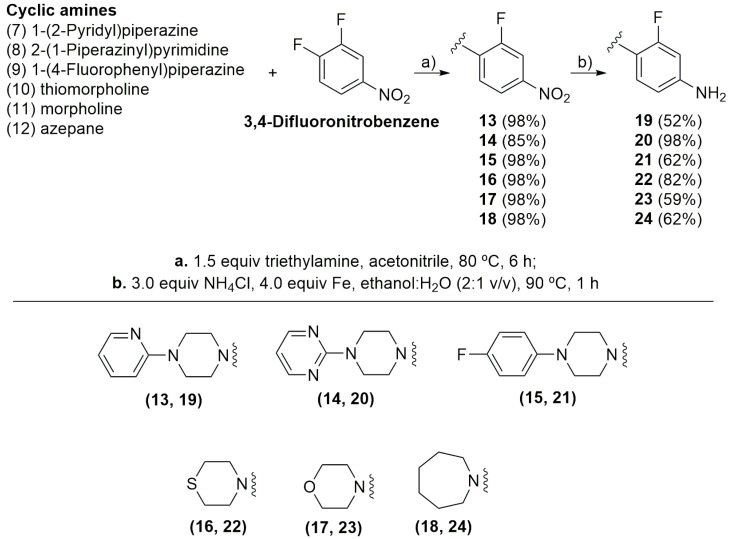
Synthesis of nitro (**13**–**18**) and amine aromatics (**19**–**24**).

The synthesis of the target series **29**–**40**, **44**, and **45** started with the preparation of intermediates **13**–**18** (Scheme 1), implying, firstly, the expected regioselective *p*-SN_2_Ar amination of 3,4-difluoronitrobenzene carried out with a variety of cyclamines.

The second step consisted of reductive conversion of nitro derivatives **13**–**18** into the corresponding amines **19**–**24** in classical Béchamp conditions.

**Scheme 2:**
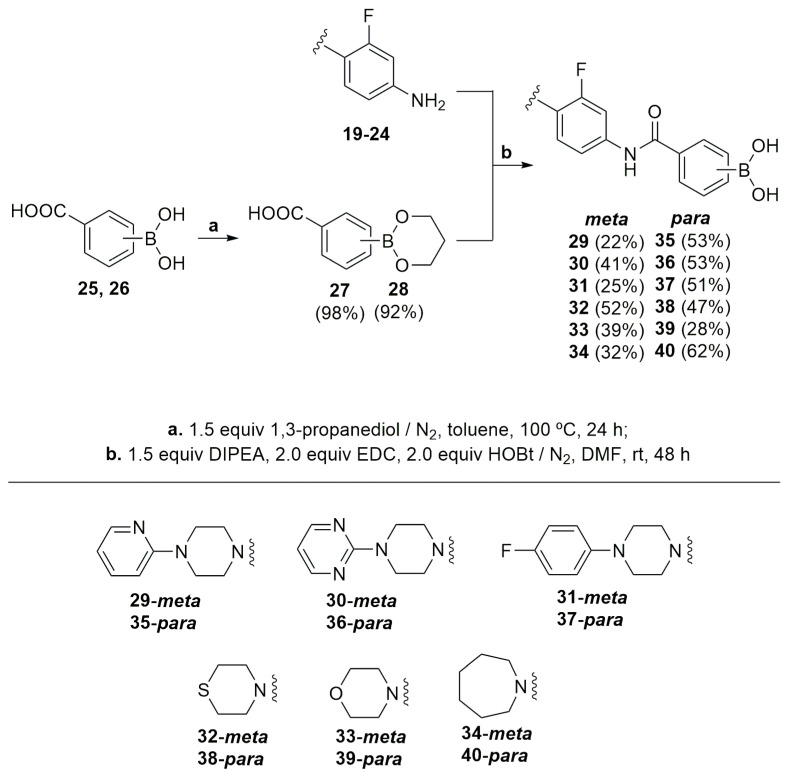
Synthesis of boronic acid derivatives (**29**–**40**).

In the final step, direct amidation coupling between 3-carboxyphenylboronic acid **25** or 4-carboxyphenylboronic acid **26** and arylamines **19**–**24** was performed. *N*-(3-dimethylaminopropyl)-*N’*-ethylcarbodiimide (EDC) was used as the coupling agent, yielding the boronic derivatives **29**–**40** [21,22,23] (Scheme 2).

#### 2.1.2. Synthesis of Benzoxaboroles Derivatives

**Scheme 3:**
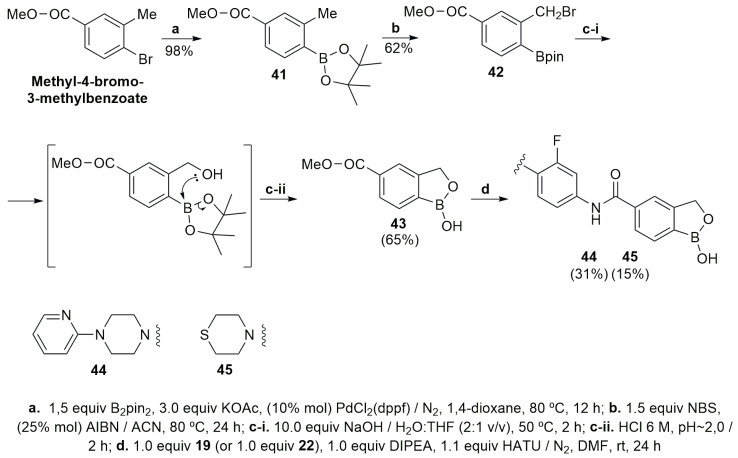
Synthesis of benzoxaborole phenyl amide **44** and **45**.

First (a), the bromine in the commercial 4-bromo-3-methylbenzoate was replaced by the pinacol boronic acid ester group, applying the Miyaura borylation protocol [24,25,26] to afford the arylboronic acid **41** as a white solid (98% yield). The second step (b) involved the Wohl–Ziegler benzylic bromination of **41** with the use of the tandem NBS/AIBN to obtain compound **42** (yellow oil, 62% yield). The third stage (c) occurred in a one-pot two-step manipulation (**c-i** and **c-ii**), the first being the alkaline SN hydroxylating replacement of bromine (**c-i**); this was followed by the intramolecular transesterification/acidolysis of the pinacol boronic ester **(c-ii**), thus leading to a five-membered oxaborole ring closure [27]. In addition, the same acidolysis conditions also favored formation of the free carboxyl termini group, producing the benzoxaborole acid **43** (slightly yellow solid, 65% yield). The last step (**d**) consisted of the direct amidation coupling between the carboxylic benzoxaborole acid **43** and fluorinated phenyl amines **19** and **22**, carried out in the presence of azabenzotriazole tetramethyl uranium (HATU) [23], producing benzoxaborole phenyl amides **44** and **45** (31% and 15% yields, respectively, solid materials) (Scheme 3).

### 2.2. Biological Assay

#### 2.2.1. In Vitro Antimicrobial Activity Against *Mycobacterium tuberculosis* and Fungi

All the compounds were evaluated against the inhibition activity of the *M. tuberculosis* H37RV ATCC 27294 strain, using the resazurin microtiter assay (REMA) method. The results were determined using the minimum inhibitory concentration (MIC_90_), which is the minimum concentration required to inhibit 90% of the mycobacterium’s activity [28]. As in-house protocol, we established a cut-off for the MIC_90_ as being less than 25 µM [29]. All the compounds, **29**–**40**, **44**, and **45**, were considered inactive, with a MIC_90_ value above 25 µM (Table 1).

Compounds **29**–**40**, **44**, and **45** were evaluated for their antifungal activity against *C. albicans* (ATCC 90028), *T. rubrum* (ATCC 28189), and *T. mentagrophytes* (ATCC 11481). The MIC_90_ values were determined, along with the minimum fungicidal concentration (MFC), which represents the lowest concentration required to kill the fungi. Compound **34** demonstrated promising inhibition activity against *T. rubrum* and *T. mentagrophytes* with MIC_90_ values of 42.0 μM and 21.0 μM, respectively. Compound **40** did not exhibit inhibition activity comparable to **34**, but it did demonstrate activity with MIC_90_ values of 88.0 μM and 42.0 μM against *T. rubrum* and *T. mentagrophytes*, respectively. Compound **44** exhibits inhibition activity against *T. mentagrophytes* with an MIC_90_ of 146 µM (Table 1). None of the compounds exhibited activity against *C. albicans*.

#### 2.2.2. Cellular Viability

For the cytotoxicity assay, the HaCaT cell line, which is characterized as immortalized normal human keratinocytes, was used. The cytotoxic effects of the most active antifungal compounds **34**, **40**, **44**, and **45** were evaluated. It was observed that the boronic acid derivatives **34** and **40** exhibited higher cytotoxic effects compared to the benzoboroxole derivatives **44** and **45**.

**Figure 3:**
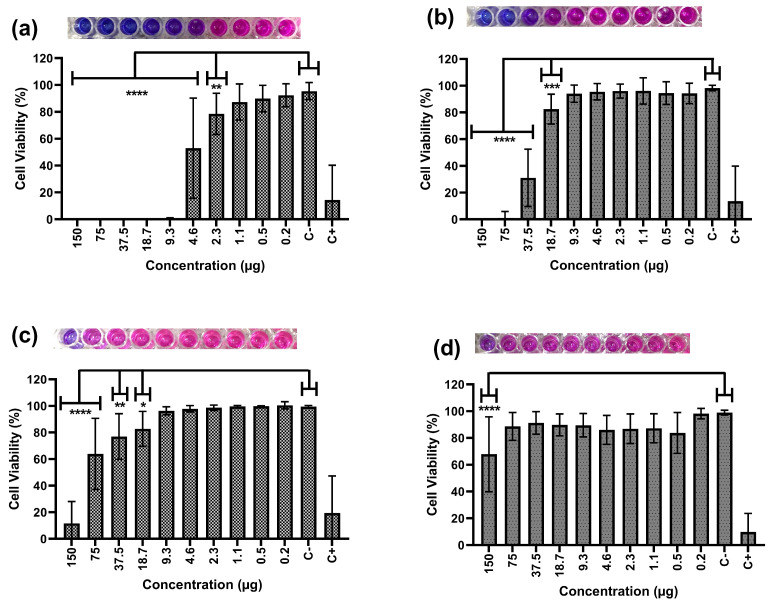
(**a**) Resazurin cytotoxicity assay using HaCaT cells for compound **24**, (**b**) for compound **30**, (**c**) for compound **36**, and (**d**) for compound **37**. **** *p* < 0.0001 *** *p* < 0.001 ** *p* < 0.01 * *p* < 0.1.

For compound **34**, no cytotoxic effects were observed at concentrations ranging from 0.2 to 1.1 μg/mL. Similarly, for compound **40**, the absence of cytotoxic effects was noted at concentrations between 0.2 and 9.3 μg/mL. In contrast, the benzoboroxole derivatives **44** and **47** did not exhibit cytotoxic effects at concentrations ranging from 0.2 to 75 μg/mL (Figure 3).

The distribution coefficient (cLog D) of the boronic derivatives was calculated using ChemAxon’s calculator (Table 1) to assess the impact of this physicochemical property on their biological evaluation. All the compounds exhibited cLogD values ranging from 2.76 to 5.19. For the most active antifungal compounds **34**, **40**, **44**, and **45**, the calculated cLogD values were 4.22, 4.22, 4.9, and 3.8, respectively. These compounds displayed high lipophilicity, which is consistent with the characteristics of antifungal drugs effective against dermatophytes.

#### 2.2.3. In Vitro Anticancer Activity

Boronic acid and benzoboroxole derivatives have been identified as potential anticancer agents [3,9,20,30]. To investigate their efficacy against head and neck cancer cell lines, SCC-25 cells were utilized in this study, as a model for oral squamous cell carcinoma. Additionally, cytotoxicity was performed in normal oral keratinocytes NOK-si cells to evaluate the selective index of the compounds. A compound is considered promising when it demonstrates high cytotoxicity against tumor cells while exhibiting minimal toxicity toward normal cells. The selectivity of the compounds is quantified by the selectivity index (SI), with those with an SI greater than 3.0 being considered promising for further evaluation, in accordance with internal protocols. Among the fourteen boronic acid derivatives tested, compound **35** exhibited the highest potency against SCC-25 cells (IC_50_: 45.01 μM) with a selectivity index of 3.37. It displayed minimal toxicity to NOK-si cells (IC_50_: 220.7 μM), demonstrating its potential for further investigation (Table 2). In contrast, its regioisomer **29** showed low inhibition activity (SCC-25 IC_50_: 79.06 μM), but non-selectivity (SI < 1.0) (Table 2). The benzoboroxol derivative **44** IC_50_ values were not determined (N/A) for either SCC-25 or NOK-si cells due to its absent killing effect in SCC-25 cells (Table 2).

Like compound **35**, compound **31** decreased SCC-25 cell viability (IC_50_: 59.07 µM) with excellent selectivity (SI: 3.73) and no cytotoxicity effect on NOK-si cells (IC_50_ > 220.7 µM). However, its derivative **27** showed moderate inhibition (IC_50_: 79.06 µM) and lacked significant selectivity (SI: 1.55) (Table 2). The compounds **34**, **39**, and **40** exhibited moderate inhibitory effects against SCC-25 cells (IC_50_: 67.57, 74.09, and 85.09 μM, respectively), but displayed low selectivity, as evidenced by their selectivity index (SI) values below 3.0 (SI: 1.91, 1.04, and 1.49, respectively). Derivative **36** demonstrated weak inhibitory activity against SCC-25 cells (IC_50_: 133.4 μM) and was also cytotoxic to NOK-si cells (IC_50_: 133.5 μM), whereas compound **45** exhibited the highest cytotoxicity towards NOK-si cells within the series (IC_50_: 65.77 μM). It was not feasible to ascertain the IC_50_ values for compounds **30**, **32**, and **33** against either cell line (Table 2). These data suggested the potential of compounds **31** and **35** as prototypes for further optimization, aiming towards new anticancer agents for the treatment of oral cancers.

## 3. Discussion

In recent years, there has been growing interest in the application of boron derivatives in medicinal chemistry, following the approval of new drugs, such as crisaborole, bortezomib, and tavaborole. The distinctive ability of boronic acid and benzoboroxole derivatives to form reversible covalent bonds with amino acid residues has inspired the exploration of new therapeutic agents for the treatment of cancer, inflammation, and microbial infections [31,32].

In previous work conducted by our research group, we identified potent benzofuroxan derivatives with activity against *Mycobacterium tuberculosis*, exhibiting MIC_90_ values of 0.09 µM [19]. Transcriptomic studies indicated that the mechanism of action of these compounds involved the inhibition of protein synthesis. A similar mechanism of action has been reported for ganfeborole (GSK-3036656, MIC_90_: 0.08 µM), which inhibits the leucyl-tRNA synthetase (LeuRS) enzyme [14]. These findings led us to hypothesize that the biosynthetic substitution of the benzofuroxan scaffold with benzoboroxole and boronic acid derivatives could further optimize anti-*Mtb* activity. To test this hypothesis, fourteen new derivatives were synthesized and evaluated as antimicrobial agents, yielding overall synthesis rates between 15% and 62%.

The presence of boronic acids has been investigated in the development of new anti-*MtB* drugs. Umerisi and colleagues synthesized and tested a series of derivatives containing this functional group as inhibitors of the enoyl acyl carrier protein reductase (InhA) enzyme, which plays a key role in the biosynthesis of fatty acids, including mycolic acid. The MIC_90_ values ranged from 0.083 to 0.125 mg/mL, highlighting the potential of this chemical group in anti-*Mtb* activity [33]. In another study, Chatterjee and collaborators explored the ability of boronic acids to inhibit the LexA/RecA system in mycobacteria. This system is critical for the “SOS” response, and its inhibition disrupts the mycobacteria’s ability to repair DNA damage under stress conditions. Molecular modeling studies indicated that boronic acid can interact with the catalytic residues (S160 and K197) of the *Mtb* LexA enzyme [34]. The reactivity of boronic acids with glycans in the mycobacterial membrane structure has prompted the synthesis of dimeric boronic acid derivatives, aimed at forming reversible covalent bonds with the 1,2- and 1,3-diols found in the carbohydrate structure of the *Mtb* membrane. It is well understood that the glycan layer of mycobacteria is vital for its survival and pathogenesis, and disruption of this structure can result in the bacillus’s death. In this study, the researchers identified boronic acid derivatives with MIC_90_ values ranging from 0.78 to 12.5 mM. Additionally, the authors proposed optimal linker distances, ranging from 1.59 to 9.68 nm, to facilitate binding with the glycans in the *Mtb* cell wall [35,36]. Boronic acid transition state inhibitors (BATSIs) are recognized as an important class of beta-lactamase inhibitors. The BlaC enzyme is known to be involved in the inactivation of beta-lactam antibiotics, and the combination of meropenem with clavulanate has been shown to have sterilizing effects on *Mtb* cultures. In this regard, and given the ability of boronic acids to form reversible covalent bonds, Kurz and colleagues described new boronic acid derivatives that mimic the transition state and inhibit the BlaC enzyme in mycobacteria, offering a promising new approach in the search for antimycobacterial agents [37]. In light of the potential of boronic acids and benzoboroxol, and leveraging a bioisosterism strategy, this study investigates the capacity of these compounds to inhibit mycobacterial growth. However, exploratory studies assessing their activity against *M. tuberculosis* did not confirm the anticipated bioisosteric relationship, as the compounds showed no significant activity against the bacillus, with MIC_90_ values exceeding 25 µM. One possible reason for the lack of effect is that benzofuroxan compounds may exert additional influences, such as by increasing oxidative stress and generating free radicals, which could enhance their anti-*Mtb* activity [19,29].

Alongside the antimycobacterial assays, we investigated the effects of the compounds on fungal cells to assess their activity in eukaryotic systems. This investigation aimed to explore the spectrum of action of the compounds and conduct preliminary screening, given that tavaborole had been previously reported as an antifungal agent against dermatophytes. Tavaborole has been reported to exhibit fungistatic effects, with MIC values of 8 μg/mL against *T. mentagrophytes* and *T. rubrum* [38]. The rates of mycological cure and complete cure have been observed to reach up to 36% and 10%, respectively [39]. Furthermore, resistance to tavaborole has already been documented, highlighting the need for the discovery of new antifungal agents.

The antifungal properties of organoboron compounds have been extensively documented [40]. Notably, these compounds have been shown to interfere with carbohydrate metabolism and other metabolic pathways essential for fungal growth and reproduction [41]. For instance, boric acid disrupts hyphal transformation and the development of fungal biofilms [42]. Campbell and colleagues synthesized a series of 3-substituted-2(5*H*)-oxaboroles with antifungal activity against several species, including *T. mentagrophytes*, *P. chrysogenum*, and *A. flavus*, with MIC values ranging from 5.2 to 6.25 μg/mL. The planarity of the system was found to enhance the antifungal effects, and none of the compounds exhibited hemolytic effects or cytotoxicity against rat myoblast cells (H9c2) [43]. Borys and colleagues have reported the antifungal properties of a series of 2-formylphenylboronic acids, with MIC values ranging from <1 to 125 μg/mL against *Aspergillus*, *Fusarium*, *Penicillium*, and *Candida* species. Notably, these derivatives exhibit cyclic tautomers in solution, leading to the formation of 3-hydroxybenzoxaborole [44]. A similar approach was reported by Adamczyk-Woźniak and colleagues, who identified 5-trifluoromethyl-2-formyl phenylboronic acid as an inhibitor of leucyl-tRNA synthetase (LeuRS). The authors observed MIC values ranging from 8 to 250 μg/mL against *Candida albicans*, *Aspergillus niger*, *Escherichia coli*, and *Bacillus cereus* [45].

In our studies, we evaluated the antifungal activity of the compounds against the dermatophytes *T. rubrum* (ATCC 28189) and *T. mentagrophytes* (ATCC 11481), as well as against *C. albicans* (ATCC 90028). Compounds **34**, **40**, **44**, and **45** demonstrated moderate antifungal activity, with compound 34 showing MIC_90_ values of 42.0 μM and 21.0 μM against *T. rubrum* and *T. mentagrophytes*, respectively. Although the boronic acid derivatives exhibited stronger antifungal activity than the benzoboroxole derivatives, cytotoxicity studies using HaCaT cells revealed that the latter showed lower toxicity. The benzoboroxole derivatives did not exhibit cytotoxic effects at concentrations ranging from 0.2 to 75 μg/mL. No clear structure–activity relationship was identified for this series; however, compounds containing 3- or 4-(azepan-1-yl) (**34** and **40**, respectively) exhibited promising antifungal effects, suggesting their potential for further molecular optimization.

To explore potential secondary targets involved in the action of the compounds, and in light of the observed cytotoxic effects, we proceeded to screen for anticancer activity. The anticancer activity of boron-containing compounds has been well documented in the literature [20], which inspired the evaluation of these compounds against tumor cells. Head and neck cancers and oral squamous cell carcinoma rank among the fifth most common cancer types and can present challenges in treatment [46]. Boronic acid has been shown to inhibit the proliferation of head and neck cancer cell lines, with GI_50_ values ranging from 800 to 1600 µg/mL [47]. Additionally, boric acid was evaluated for its effects on medullary thyroid cancer, revealing an IC_50_ of 35 µM after 48 h of treatment, through the modulation of apoptotic pathways [48]. In addition to bortezomib, other organoboron drugs, such as ixazomib, have been shown to treat cancers with greater efficacy and safety compared to bortezomib [32]. Furthermore, non-dipeptide boron derivatives have demonstrated potent anticancer effects against head and neck cancer cell lines. For instance, Kong and collaborators synthesized novel tubulin polymerization derived from combretastatin, where a hydroxyl group was replaced by a boronic acid. These compounds showed activity across 16 human cancer cell lines, with MIC values ranging from 10 to 200 nM. In the case of SNC, they tested the compounds on cell lines SF-268, SF-295, SF-539, SNB-19, and U251, reporting GI50 values ranging from <0.01 to 2.73 µM [49]. These findings prompted us to explore the anticancer effects of the synthesized boronic acid and benzoboroxole derivatives. During the screening, two promising prototype compounds, **31** and **35**, demonstrated potent activity against SCC-25 cells, with IC_50_ values of 59.07 µM (**31**) and 45.61 µM (**35**), and SI values of 3.73 (**31**) and 3.37 (**35**), while showing no cytotoxicity toward non-tumoral NOK-si cells (IC_50_ > 220.7 µM). Like the findings in antifungal activity, no clear structure–activity relationship was identified for the anticancer effects.

Thus, in this screening study, we identified the benzoxaborole compounds **44** and **45** as promising prototypes for the development of new antifungal agents, while boronic acid derivatives emerged as potential prototypes for anticancer agents. The identification of these prototype compounds represents a key paradigm in medicinal chemistry, enabling continuous optimization in the pursuit of new therapeutic agents for the treatment of infectious diseases and cancer.

## 4. Experimental

A. General

All the reagents were of analytical purity and purchased from Acros, Alfa Aesar, Oakwood Chemical, Sigma-Aldrich, and Synth, with no need for additional purification, except for 1,4-dioxane, acetonitrile, and toluene which were dried over molecular sieves porosities 5 Å and 1.6 Å, (Sigma-Aldrich, St Louis, MO, USA) prior to use. Sensitive reactions were carried out under an inert atmosphere (N_2_).

Melting points were determined using Stuart Scientific^®^ Melting Point Apparatus SMP3 capillary equipment (Bibby Stuart Scientific, Cole-Parmer, Staffordshire, UK) and were not corrected. TLC was carried out on aluminum sheets (plates) with silica gel 90 Å Merck^®^ (visualization with fluorescence indicator under UV at λ = 254 nm). Column chromatography was conducted on Sigma-Aldrich^®^ 60 Å silica gel (40–60 μm, 230–400 mesh) (Sigma-Aldrich, St Louis, MO, USA) using ethyl acetate, n-hexane, and methanol as eluents (mobile phase).

IR spectra (KBr) were recorded using Prestige-21 Shimadzu^®^ equipment (Shimadzu, Kyoto, Japan) operating in the range absorption between 400 and 4000 cm^−1^. Only relevant absorption maxima (ν_max_) are listed as s (strong), m (medium), and w (weak). 1D-^1^H- and -^13^C-NMR spectra were carried out using a Bruker Fourier 300 spectroscope (Bruker Corporation, Billerica, MA, USA) operating at 300 and 75 MHz for ^1^H and ^13^C nuclei, respectively. 1D-ATP and 2D-^1^H, ^13^C-HSQS, -HMBC spectra/charts were obtained using Bruker Avance III 600 (Bruker Corporation, Billerica, Massachusetts, USA) equipment operating at 600 and 150 MHz for ^1^H and ^13^C nuclei, respectively. 1D-ATP and 2D-^1^H, ^13^C-HSQS, -HMBC spectra/charts were obtained using Bruker Avance III 600 equipment operating at 600 and 150 MHz for ^1^H and ^13^C nuclei, respectively. All chemical shifts (*δ*_X_ values, X = ^1^H and ^1^) are given in parts per million (ppm); all homo- and heterocoupling patterns (^n^*J*_C,F_ values) are given in Hertz. No TMS was added; the chemical shifts were measured against the solvent peak: Acetone-*d*_6_ (*δ*_H_ = 2.09 and *δ*_C_ = 205.87 ppm), CDCl_3_ (*δ*_H_ = 7.26 and *δ*_C_ = 77.36 ppm) and DMSO-*d*_6_, (*δ*_H_ = 2.50 and *δ*_C_ = 39.52 ppm). The multiplicity of ^1^H and ^13^C signals was assigned as: singlet (s), doublet (d), triplet (t), quadruplet (q), double doublet (dd), double double of doublet (ddd), and multiplet (m).High-resolution mass spectrometry (ESI+) spectra were obtained by using the Bruker Maxis Impact quadrupole time-of-flight tandem mass spectrometer (Q-TOF MS/MS) (Bruker Corporation, Billerica, MA, USA). 

For further details, please refer to the Supplementary Materials, where the spectra for all analytical methods are described.

B. General Procedure for the Synthesis of Nitroaromatic Intermediates **13**–**18** [17,19]

At room temperature, under inert atmosphere (N_2_) and after 15 min. of vigorous stirring, 3,4-difluoronitrobenzene (7.03 mmol, 1.0 equiv.) was added to an anhydrous acetonitrile (8 mL) solution containing (7.03 mmol, 1 equiv.) of phenyl amine **7**–**12** and triethylamine (10.55 mmol, 1.5 equiv.). The reaction mixtures were heated at reflux for 6 h. After cooling at room temperature, the solvent and the excess triethylamine were removed to dryness under reduced pressure. The resulting residues were purified by flash column chromatography (eluent n-hexane/AcOEt 1:1 *v*/*v*) [17,19].

*1-(2-Fluoro-4-nitrophenyl)-4-(pyridin-2-yl)piperazine* **13** yellow solid; yield 98%; mp: 152–154 °C (n-hexane/AcOEt, 5:5); ^1^H-NMR (600 MHz, DMSO-*d*_6_) *δ*_H_ 8.15–8.17 (ddd, *J* = 4.9, 2.0, 0.8 Hz, H-6, pyridine), 8.06–8.03 (m, H-5, nitrobenzene), 8.03–8.02 (m, H-3, nitrobenzene), 7.58–7.55 (ddd, *J* = 8.8, 7.1, 2.0 Hz, H-4, pyridine), 7.23–7.20 (m, H-6, nitrobenzene), 6.89–6.88 (d, *J* = 8.8 Hz, H-3, pyridine), 6.69–6.67 (dd, *J* = 7.1, 4.9, 0.7 Hz, H-5, pyridine), 3.67–3.65 (dd, *J* = 6.2, 4.2 Hz, H-2, -6, piperazine), 3.42–3.40 (dd, *J* = 6.2, 4.9 Hz, H-3, -5, piperazine) ppm. ^13^C-NMR (150 MHz, DMSO-*d*_6_) *δ*_C_ 158.8 (C-2, pyridine), 152.9 (d, ^1^*J*_C–F_ = 247.4 Hz, C-2, nitrobenzene), 147.6 (C-6, pyridine), 145.3 (d, ^2^*J*_C–F_ = 7.6 Hz, C-1, nitrobenzene), 139.4 (d, ^3^*J*_C–F_ = 9.2 Hz, C-4, nitrobenzene), 137.7 (C-4, pyridine), 121.3 (d, ^4^*J*_C–F_ = 2.9 Hz, C-5, nitrobenzene), 118.0 (d, ^3^*J*_C–F_ = 4.3 Hz, C-6, nitrobenzene), 113.4 (C-5, pyridine), 112.3 (d, ^2^*J*_C–F_ = 26.2 Hz, C-3, nitrobenzene), 107.2 (C-3, pyridine), 48.8 (C-2, -6, piperazine), 44.3 (C-3, -5, piperazine) ppm.

*2-[4-(2-Fluoro-4-nitrophenyl)piperazin-1-yl]pyrimidine* **14** yellow solid; yield 85%; mp: 191–192 °C (n-hexane/AcOEt, 5:5); ^1^H-NMR (600 MHz, DMSO-*d*_6_) *δ*_H_ 8.41–8.40 (d, *J* = 4.7 Hz, H-4, -6, pyrimidine), 8.06–8.04 (m, H-5, nitrobenzene), 8.04–8.02 (m, H-3, nitrobenzene), 7.23–7.20 (m, H-6, nitrobenzene), 6.69–6.67 (t, *J* = 4.7 Hz, H-5, pyrimidine), 3.91–3.89 (m, H-3, -5, piperazine), 3.39–3.37 (m, H-2, -6, piperazine) ppm. ^13^C-NMR (150 MHz, DMSO-*d*_6_) *δ*_C_ 161.1 (C-2, pyrimidine), 158.0 (C-4, -6, pyrimidine), 152.9 (d, ^1^*J*_C–F_ = 247.8 Hz, C-2, nitrobenzene), 145.4 (d, ^2^*J*_C–F_ = 8.0 Hz, C-1, nitrobenzene), 139.5 (d, ^3^*J*_C–F_ = 9.4 Hz, C-4, nitrobenzene), 121.3 (d, ^4^*J*_C–F_ = 3.1 Hz, C-5, nitrobenzene), 118.1 (d, ^3^*J*_C–F_ = 4.4 Hz, C-6, nitrobenzene), 112.4 (d, ^2^*J*_C–F_ = 26.4 Hz, C-3, nitrobenzene), 110.4 (C-5, pyrimidine), 48.9 (C-3, -5, piperazine), 43.0 (C-2, -6, piperazine) ppm.

*1-(2-Fluoro-4-nitrophenyl)-4-(4-fluorophenyl)piperazine* **15** yellow solid; yield 98%; mp: 135–137 °C (n-hexane/AcOEt, 5:5); ^1^H-NMR (600 MHz, DMSO- *d*_6_) *δ*_H_ 8.06–8.04 (dd, *J* = 6.4, 2.0 Hz, H-5, fluorobenzene), 8.04–8.03 (m, H-3, nitrobenzene), 7.23–7.22 (m, H-6, nitrobenzene), 7.10–7.06 (m, H-3, -5, fluorobenzene), 7.03–7.01 (m, H-2, -6, fluorobenzene), 3.45–3.43 (dd, *J* = 4.7, 2.4 Hz, H-2, -6, piperazine), 3.25–3.24 (dd, *J* = 4.7, 2.4 Hz, H-3, -5, piperazine) ppm. ^13^C-NMR (150 MHz, DMSO-*d*_6_) *δ*_C_ 157.1 (d, ^1^*J*_C–F_ = 236.1 Hz, C-4, fluorobenzene), 152.9 (d, ^1^*J*_C–F_ = 247.5 Hz, C-2, nitrobenzene), 147.6 (d, ^4^*J*_C–F_ = 2.3 Hz, C-1, fluorobenzene), 145.2 (d, ^2^*J*_C–F_ = 7.7 Hz, C-1, nitrobenzene), 139.6 (d, ^3^*J*_C–F_ = 9.4 Hz, C-4, nitrobenzene), 121.3 (d, ^4^*J*_C–F_ = 2.9 Hz, C-5, nitrobenzene), 118.1 (d, ^2^*J*_C–F_ = 4.4 Hz, C-3, -5, fluorobenzene), 117.5 (d, ^3^*J*_C–F_ = 7.9 Hz, C-6, nitrobenzene), 115.4 (d, ^3^*J*_C–F_ = 22.0 Hz, C-2, -6, fluorobenzene), 112.3 (d, ^2^*J*_C–F_ = 26.5 Hz, C-3, nitrobenzene), 49.1 (C-2, -6, piperazine), 48.9 (C-3, -5, piperazine) ppm.

*4-(2-Fluoro-4-nitrophenyl)thiomorpholine* **16** orange solid; yield 98%; mp: 54–56 °C (n-hexane/AcOEt, 5:5); ^1^H-NMR (600 MHz, CDCl_3_) *δ*_H_ 7.97 (dd, *J* = 9.0, 1.9 Hz, H-5, nitrobenzene), 7.89 (dd, *J* = 12.9, 2.6 Hz, H-3, nitrobenzene), 6.92 (t, *J* = 8.8 Hz, H-6, nitrobenzene), 3.59–3.56 (m, H-3, -5, thiomorpholine), 2.81–2.78 (m, H-2, -6, thiomorpholine) ppm. ^13^C-NMR (150 MHz, CDCl_3_) *δ*_C_ 153.9 (d, ^1^*J*_C–F_ = 249.0 Hz, C-2, nitrobenzene), 146.0 (d, ^2^*J*_C–F_ = 7.6 Hz, C-1, nitrobenzene), 140.7 (d, ^3^*J*_C–F_ = 8.7 Hz, C-4, nitrobenzene), 121.1 (d, ^4^*J*_C–F_ = 2.6 Hz, C-5, nitrobenzene), 117.9 (d, ^3^*J*_C–F_ = 3.5 Hz, C-6, nitrobenzene), 112.9 (d, ^2^*J*_C–F_ = 26.5 Hz, C-3, nitrobenzene), 52.5 (C-3, -5, thiomorpholine), 27.5 (C-2, -6, thiomorpholine) ppm [19].

*4-(2-Fluoro-4-nitrophenyl)morpholine* **17** orange solid; yield 98%; mp: 109–111 °C (n-hexane/AcOEt, 5:5); ^1^H-NMR (600 MHz, CDCl_3_) *δ*_H_ 7.99 (ddd, *J* = 9.0, 2.6, 0.9 Hz, H-5, nitrobenzene), 7.91 (dd, *J* = 13.1, 2.6 Hz, H-3, nitrobenzene), 6.92 (t, *J* = 8.8 Hz, H-6, nitrobenzene), 3.89–3.86 (m, H-2, -6, morpholine), 3.29–3.26 (m, H-3, -5, morpholine) ppm. ^13^C-NMR (150 MHz, CDCl_3_) *δ*_C_ 154.1 (d, ^1^*J*_C–F_ = 249.5 Hz, C-2, nitrobenzene), 145.6 (d, ^2^*J*_C–F_ = 7.4 Hz, C-1, nitrobenzene), 141.0 (d, ^3^*J*_C–F_ = 8.7 Hz, C-4, nitrobenzene), 121.1 (d, ^4^*J*_C–F_ = 2.7 Hz, C-5, nitrobenzene), 117.0 (d, ^3^*J*_C–F_ = 3.9 Hz, C-6, nitrobenzene), 112.8 (d, ^2^*J*_C–F_ = 26.3 Hz, C-3, nitrobenzene), 66.7 (C-3, -5, morpholine), 50.0 (C-2, -6 morpholine) ppm [19].

*1-(2-Fluoro-4-nitrophenyl)azepane* **18** yellow solid; yield 98%; mp: 32–33 °C (n-hexane/AcOEt, 5:5); ^1^H-NMR (600 MHz, CDCl_3_) *δ*_H_ 7.91 (dd, *J* = 9.3, 2.7 Hz, H-5, nitrobenzene), 7.86 (dd, *J* = 15.0, 2.6 Hz, H-3, nitrobenzene), 6.71 (t, *J* = 9.2 Hz, H-6, nitrobenzene), 3.57 (td, *J* = 6.3; 1.5 Hz, H-2, -7, azepane), 1.87–1.82 (m, H-3, -5, azepane), 1.63–1.56 (m, H-4, -5, azepane) ppm. ^13^C-NMR (150 MHz, CDCl_3_) *δ*_C_ 150.6 (d, ^1^*J*_C–F_ = 245.1 Hz, C-2, nitrobenzene), 144.4 (d, ^2^*J*_C–F_ = 7.0 Hz, C-1, nitrobenzene), 136.7 (d, ^3^*J*_C–F_ = 8.5 Hz, C-4, nitrobenzene), 121.7 (d, ^4^*J*_C–F_ = 1.4 Hz, C-5, nitrobenzene), 113.8 (d, ^3^*J*_C–F_ = 5.5 Hz, C-6, nitrobenzene), 113.5 (d, ^2^*J*_C–F_ = 27.8 Hz, C-3, nitrobenzene), 52.3 (C-2, -7, azepane), 28.5 (C-3, -6, azepane), 26.9 (C-4, -5, azepane) ppm [19].

C. General Procedure for the Synthesis of Amine Aromatic Intermediates **19**–**24** [17,19]

In a mixture of EtOH (15.0 mL)/H_2_O (7.5 mL), (3.31 mmol, 1.0 equiv.) of the corresponding nitro aromatic intermediates **13**–**18** were added, followed by the addition of (9.93 mmol, 3.0 equiv.) of ammonium chloride and (13.24 mmol, 4.0 equiv.) of metallic iron, in a 50.0 mL round-bottom flask, with stirring for 1 h and refluxing. Then, the reaction mixture was vacuum filtered with the aid of celite 545 and washed in methanol; the resulting solvent was removed under reduced pressure. Soon afterwards, the resulting mixture was solubilized in 50.0 mL of dichloromethane and washed (3 × 50.0 mL) with saturated sodium chloride solution and (3 × 50.0 mL) saturated sodium bicarbonate solution. The organic phase was collected and the solvent removed under reduced pressure. The crude product was purified by column chromatography (eluent n-hexane/AcOEt, 6:4) [17,19].

*3-Fluoro-4-[4-(pyridin-2-yl)piperazin-1-yl]aniline* **19** brown solid; yield 52%; mp: 92–95 °C (n-hexane/AcOEt, 6:4); ^1^H-NMR (600 MHz, DMSO-*d*_6_) *δ*_H_ 8.13–8.11 (ddd, *J* = 4.9, 2.0, 0.8 Hz, H-6, pyridine), 7.55–7.52 (ddd, *J* = 8.7, 7.1, 2.0 Hz, H-4, pyridine), 6.86–6.84 (d, *J* = 8.7 Hz, H-3, pyridine), 6.82–6.78 (dd, *J* = 9.9, 8.6 Hz, H-5, aniline), 6.66–6.64 (ddd, *J* = 7.1, 4.9, 0.8 Hz, H-5, pyridine), 6.37–6.34 (dd, *J* = 14.4, 2.5 Hz, H-2, aniline), 6.32–6.30 (m, H-6, aniline), 5.01 (s, 2H, NH_2_), 3.59–3.57 (m, H-2, -6, piperazine), 2.91–2.90 (m, H-3, -5, piperazine) ppm. ^13^C-NMR (150 MHz, DMSO-*d*_6_) *δ*_C_ 159.0 (C-2, pyridine), 157.1 (d, ^1^*J*_C–F_ = 241.6 Hz, C-3, aniline), 147.6 (C-6, pyridine), 145.6 (d, ^3^*J*_C–F_ = 11.0 Hz, C-1, aniline), 137.7 (C-4, pyridine), 129.0 (d, ^2^*J*_C–F_ = 10.3 Hz, C-4, aniline), 120.8 (d, ^4^*J*_C–F_ = 4.9 Hz, C-5, aniline), 113.1 (C-5, pyridine), 109.3 (d, ^3^*J*_C–F_ = 2.9 Hz, C-6, aniline), 107.1 (C-3, pyridine), 101.9 (d, ^2^*J*_C–F_ = 23.5 Hz, C-2, aniline), 51.1 (C-3, -5, piperazine), 49.0 (C-2, -6, piperazine) ppm.

*3-Fluoro-4-[4-(pyrimidin-2-yl)piperazin-1-yl]aniline* **20** brown solid; yield 98%; mp: 81–82 °C (n-hexane/AcOEt, 6:4); ^1^H-NMR (600 MHz, DMSO-*d*_6_) *δ*_H_ 8.37–8.36 (d, *J* = 4.7 Hz, H-6, -4, pyrimidine), 6.81–6.77 (dd, *J* = 9.9, 8.6 Hz, H-5, aniline), 6.64–6.63 (t, *J* = 4.7 Hz, H-5, pyrimidine), 6.37 (dd, *J* = 14.4, 2.5 Hz, H-2, aniline), 6.31–6.29 (m, H-6, aniline), 5.01 (s, 2H, NH_2_), 3.84–3.83 (m, H-2, -6, piperazine), 2.87–2.85 (m, H-3, -5, piperazine) ppm. ^13^C-NMR (150 MHz, DMSO-*d*_6_) *δ*_C_ 161.2 (C-2, pyrimidine), 157.9 (C-4, -6, pyrimidine), 157.2 (d, ^1^*J*_C–F_ = 241.3 Hz, C-3, aniline), 145.7 (d, ^1^*J*_C–F_ = 11.2 Hz, C-1, aniline), 129.1 (d, ^1^*J*_C–F_ = 10.0 Hz, C-4, aniline), 120.9 (d, ^1^*J*_C–F_ = 5.0 Hz, C-5, aniline), 110.2 (C-5, pyrimidine), 109.5 (d, ^1^*J*_C–F_ = 3.0 Hz, C-6, aniline), 101.8 (d, ^1^*J*_C–F_ = 25.2 Hz, C-2, aniline), 51.2 (C-3, -5, piperazine), 43.6 (C-2, -6, piperazine) ppm.

*3-Fluoro-4-[4-(4-fluorophenyl)piperazin-1-yl]aniline* **21** brown solid; yield 62%; mp: 161–164 °C (n-hexane/AcOEt, 6:4); ^1^H-NMR (600 MHz, DMSO-*d*_6_) *δ*_H_ 7.07–7.04 (m, H-3, -5, fluorobenzene), 6.99–6.97 (dt, *J* = 4.5, 2.3 Hz, H-2, -6, fluorobenzene), 6.82–6.79 (dd, *J* = 9.9, 8.5 Hz, H-5, aniline), 6.37–6.34 (dd, *J* = 14.4, 2.5 Hz, H-2, aniline), 6.33–6.31 (dd, *J* = 8.4, 2.5 Hz, H-6, aniline), 5.01 (s, 2H, NH_2_), 3.19–3.17 (m, H-2, -6, piperazine), 2.98–2.95 (m, H-3, -5, piperazine) ppm. ^13^C NMR (150 MHz, DMSO-*d*_6_) *δ*_C_ 157.1 (d, ^1^*J*_C–F_ = 242.1 Hz, C-4, fluorobenzene), 156.9 (d, ^1^*J*_C–F_ = 235.3 Hz, C-3, aniline), 147.9 (d, ^4^*J*_C–F_ = 2.1 Hz, C-1, fluorobenzene), 145.5 (d, ^2^*J*_C–F_ = 11.0 Hz, C-1, aniline), 128.9 (d, ^3^*J*_C–F_ = 10.1 Hz, C-4, aniline), 120.6 (d, ^4^*J*_C–F_ = 5.0 Hz, C-5, aniline), 117.3 (d, ^3^*J*_C–F_ = 8.2 Hz, C-2, -6, fluorobenzene), 115.3 (d, ^2^*J*_C–F_ = 22.0 Hz, C-3, -5, fluorobenzene), 109.5 (d, ^4^*J*_C–F_ = 2.8 Hz, C-6, aniline), 101.9 (d, ^2^*J*_C–F_ = 23.7 Hz, C-2, aniline), 51.2 (C-3, -5, piperazine), 49.4 (C-2, -6, piperazine) ppm.

*3-Fluoro-4-thiomorpholinoaniline* **22** brown solid; yield 82%; mp: 63–66 °C (n-hexane/AcOEt, 6:4); ^1^H-NMR (600 MHz, CDCl_3_) *δ*_H_ 6.81 (t, *J* = 8.8 Hz, H-5, aniline), 6.41 (dd, *J* = 13.3, 2.3 Hz, H-2, aniline), 6.38 (dd, *J* = 8.3 Hz, H-6, aniline), 3.56 (s, 2H, NH_2_), 3.24–3.16 (m, H-3, -5, thiomorpholine), 2.84–2.76 (m, H-2, -6, thiomorpholine) ppm. ^13^C-NMR (150 MHz, CDCl_3_) *δ*_C_ 157.9 (d, ^1^*J*_C–F_ = 245.3 Hz, C-3, aniline), 143.3 (d, ^3^*J*_C–F_ = 10.4 Hz, C-1, aniline), 133.3 (d, ^2^*J*_C–F_ = 9.9 Hz, C-4, aniline), 122.0 (d, ^3^*J*_C–F_ = 4.2 Hz, C-5, aniline), 110.7 (d, ^4^*J*_C–F_ = 3.1 Hz, C-6, aniline), 103.9 (d, ^2^*J*_C–F_ = 23.7 Hz, C-2, aniline), 54.1 (C-3, -5, thiomorpholine), 28.4 (C-2, -6, thiomorpholine) ppm [19].

*3-Fluoro-4-morpholinoaniline* **23** brown solid; yield 59%; mp: 119–121 °C (n-hexane/AcOEt, 6:4); ^1^H-NMR (600 MHz, CDCl_3_) *δ*_H_ 6.81–6.77 (m, H-5, aniline), 6.43 (dd, *J* = 13.5, 2.5 Hz, H-2, aniline), 6.40 (dd, *J* = 8.4, 3.1 Hz, H-6, aniline), 3.87–3.83 (m, H-2, -6, morpholine), 3.56 (s, 2H, NH_2_), 2.98–2.94 (m, H-3, -5, morpholine) ppm. ^13^C-NMR (150 MHz, CDCl_3_) *δ*_C_ 157.6 (d, ^1^*J*_C–F_ = 245.2 Hz, C-3, aniline), 142.9 (d, ^3^*J*_C–F_ = 10.4 Hz, C-1, aniline), 131.8 (d, ^2^*J*_C–F_ = 9.7 Hz, C-4, aniline), 120.3 (d, ^3^*J*_C–F_ = 4.2 Hz, C-5, aniline), 110.7 (d, ^4^*J*_C–F_ = 3.1 Hz, C-6, aniline), 103.9 (d, ^2^*J*_C–F_ = 23.9 Hz, C-2, aniline), 67.3 (C-3, -5, morpholine), 51.8 (C-2, -6, morpholine) ppm [19].

*4-(Azepan-1-yl)-3-fluoroaniline* **24** black oil; yield 62%; (n-hexane/AcOEt, 6:4); ^1^H-NMR (600 MHz, DMSO-*d*_6_) *δ*_H_ 6.71 (dd, *J* = 10.2, 8.6 Hz, H-5, aniline), 6.30 (dd, *J* = 14.9, 2.5 Hz, H-2, aniline), 6.25 (dd, *J* = 8.5, 2.5 Hz, H-6, aniline), 4.81 (s, 2H, NH_2_), 3.07–3.03 (m, H-2, -7, azepane), 1.70–1.66 (m, H-3, -6, azepane), 1.58–1.55 (m, H-4, -5, azepane), ^13^C-NMR (150 MHz, DMSO *d*_6_) *δ*_C_ 156.1 (d, ^1^*J*_C–F_ = 243.6 Hz, C-3, aniline) ppm. 143.5 (d, ^3^*J*_C–F_ = 10.2 Hz, C-1, aniline), 133.8 (d, ^2^*J*_C–F_ = 9.4 Hz, C-4, aniline), 120.4 (d, ^3^*J*_C–F_ = 4.9 Hz, C-5, aniline), 109.6 (d, ^4^*J*_C–F_ = 3.0 Hz, C-6, aniline), 100.2 (d, ^2^*J*_C–F_ = 24.4 Hz, C-2, aniline), 53.1 (C-4, -5, azepane), 28.8 (C-3, -7, azepane), 26.8 (C-3, -6, azepane) ppm [19].

D. General Procedure for the Synthesis of Boronic Acid Derivatives **29**–**40** [19,24]

At room temperature and under nitrogen atmosphere, (0.49 mmol, 1.0 equiv.) of boronic acids **27** and **28**, (0.98, 2.0 equiv.) of the coupling agent *N*-(3-dimethylaminopropyl)-*N’*-Ethylcarbodiimide hydrochloride, and (0.98 mmol, 2.0 equiv.) of 1-hydroxybenzotriazole reagent were added to 10.0 mL of anhydrous *N’N*-dimethylformamide, in which the reaction medium was stirred for 2 h. Then, simultaneously, (0.74 mmol, 1.5 equiv.) of the aromatic amines **19**–**24** and *N*,*N*-diisopropylethylamine (DIPEA) were added, and they remained under stirring for 48 h, in N_2_, at room temperature. After this time, 50.0 mL of water was added to the reaction medium, and the resulting solution was washed with ethyl acetate (3 × 50.0 mL) in a separatory funnel. The organic phase was collected and concentrated under reduced pressure. The resulting products were purified by column chromatography (eluent, n-hexane/AcOEt/MeOH, gradient mode, starting with Hex/AcOEt 80:20 increasing the percentage of AcOEt by 10%, up to AcOEt/MeOH 90:10) [18,24].

*{3-({3-Fluoro-4-[4-(pyridin-2-yl)piperazin-1-yl]phenyl}carbamoyl)phenyl}boronic acid* **29** white solid; yield 22%; mp: 235–239 °C (n-hexane/AcOEt/MeOH, gradient mode, starting with Hex/AcOEt 80:20 increasing the percentage of AcOEt by 10%, up to AcOEt/MeOH 90:10); IR (KBr) ν_max_ 3263 (B–OH boronic acid; N–H amide), 2848 (B–O boronic acid), 1643 (C=O amide), 1600 (C=C aromatic), 1440 (C=C aromatic), 1315 (C–F allyl floride), 1242 (C–H methylene) cm^−1^. ^1^H-NMR (600 MHz, DMSO-*d*_6_) *δ*_H_ 10.30 (s, 1H, NH), 8.32 (m, H-2, carbamoyphenylboronic acid), 8.24 (s, 2H, OH), 8.15–8.13 (ddd, *J* = 4.9, 2.0 Hz, H-6, pyridine), 7.98–7.96 (dd, *J* = 7.4, 1.2 Hz, H-6, carbamoyphenylboronic acid), 7.94–7.93 (m, H-4, carbamoyphenylboronic acid), 7.75–7.72 (dd, *J* = 14.9, 2.4 Hz, H-2, fluorophenyl), 7.57–7.55 (ddd, *J* = 9.0, 7.1, 2.0 Hz, H-4, pyridine), 7.50–7.49 (m, H-5, carbamoyphenylboronic acid), 7.49–7.48 (m, H-6, fluorophenyl), 7.10–7.07 (m, H-5, flhorophenyl), 6.90–6.88 (ddd, *J* = 7.1, 4.9 Hz, H-3, pyridine), 3.65–3.63 (m, H- 2, -6, piperazine), 3.09–3.07 (m, H-3, -5, piperazine) ppm. ^13^C-NMR (150 MHz, DMSO-*d*_6_) *δ*_C_ 166.0 (1C, C=O), 158.9 (C-2, pyridine), 155.2 (d, ^1^*J*_C–F_ = 247.2 Hz, C-3, fluorophenyl), 147.6 (C-6, pyridine), 137.6 (C-4, pyridine), 137.1 (C-6, carbamoyphenylboronic acid), 135.6 (d, ^1^*J*_C–F_ = 9.4 Hz, C-4, fluorophenyl), 134.6 (d, ^3^*J*_C–F_ = 11.0 Hz, C-1, fluorophenyl), 134.1 (C-3, carbamoyphenylboronic acid), 133.4 (C-2, carbamoyphenylboronic acid), 129.2 (C-4, carbamoyphenylboronic acid), 127.4 (C-5, carbamoyphenylboronic acid), 119.4 (d, ^3^*J*_C–F_ = 4.6 Hz, C-5, fluorophenyl), 116.2 (d, ^4^*J*_C–F_ = 3.7 Hz, C-6, fluorophenyl), 113.3 (C-5, pyridine), 108.4 (d, ^2^*J*_C–F_ = 25.7 Hz, C-2, fluorophenyl), 107.2 (C-3, pyridine), 50.3 (C-3, -5, piperazine), 44.8 (C-2, -6, piperazine) ppm. HMRS (ESI^+^, methanol) C_22_H_23_BFN_4_O_3_ [M+H]^+^ = 421.1843.

*[3-({3-Fluoro-4-[4-(pyrimidin-2-yl)piperazin-1-yl]phenyl}carbamoyl)phenyl]boronic acid* **30** white solid; yield 41%; mp: 262–266 °C (n-hexane/AcOEt/MeOH, gradient mode, starting with Hex/AcOEt 80:20 increasing the percentage of AcOEt by 10%, up to AcOEt/MeOH 90:10); IR (KBr) ν_max_ 3360 (N–H amide), 3265 (B–OH boronic acid), 2908 (C–H methylene), 1647 (C=O amide), 1591 (C=C aromatic), 1442 (C=C aromatic), 1311 (B–O boronic acid), 1247 (C–F allyl floride) cm^−1^. ^1^H-NMR (600 MHz, DMSO-*d*_6_) *δ*_H_ 10.30 (s, 1H, NH), 8.40–8.39 (d, *J* = 4.7 Hz, H-4, -6, pyrimidine), 8.32 (m, H-2, carbamoyphenylboronic acid), 8.24 (s, 2H, OH), 7.98–7.97 (m, H-6, carbamoyphenylboronic acid), 7.94–7.93 (m, H-4, carbamoyphenylboronic acid), 7.75–7.72 (dd, *J* = 14.9, 2.3 Hz, H-2, fluorophenyl), 7.50–7.49 (m, H-5, carbamoyphenylboronic acid), 7.49–7.48 (m, H-6, fluorophenyl), 7.09–7.06 (m, H-5, fluorophenyl), 6.67–6.65 (t, *J* = 4.7 Hz, H-5, pyrimidine), 3.90–3.89 (m, H-2, -6, piperazine), 3.05–3.03 (m, H-3, -5, piperazine) ppm. ^13^C-NMR (151 MHz, DMSO-*d*_6_) *δ*_C_ 166.0 (1C, C=O), 161.2 (C-2, pyrimidine), 158.0 (C-4, -6, pyrimidine), 155.2 (d, ^1^*J*_C–F_ = 243.4 Hz, C-3, fluorophenyl), 137.2 (C-6, carbamoyphenylboronic acid), 135.5 (d, ^2^*J*_C–F_ = 9.4 Hz, C-4, fluorophenyl), 134.6 (d, ^3^*J*_C–F_ = 11.6 Hz, C-1, fluorophenyl), 134.1 (C-3, carbamoyphenylboronic acid), 133.4 (C-2, carbamoyphenylboronic acid), 129.2 (C-4, carbamoyphenylboronic acid), 127.4 (C-5, carbamoyphenylboronic acid), 119.4 (d, ^3^*J*_C–F_ = 4.5 Hz, C-5, fluorophenyl), 116.2 (d, ^4^*J*_C–F_ = 3.5 Hz, C-6, fluorophenyl), 110.4 (C-5, pyrimidine), 108.4 (d, ^2^*J*_C–F_ = 25.0 Hz, C-2, fluorophenyl), 50.4 (C-3, -5, piperazine), 49.4 (C-2, -6, piperazine) ppm. HMRS (ESI^+^, methanol) C_21_H_22_BFN_5_O_3_ [M+H]^+^ = 422.1798.

*[3-({3-Fluoro-4-[4-(4-fluorophenyl)piperazin-1-yl]phenyl}carbamoyl)phenyl]boronic acid* **31** white solid; yield 25%; mp: 241–246 °C (n-hexane/AcOEt/MeOH, gradient mode, starting with Hex/AcOEt 80:20 increasing the percentage of AcOEt by 10%, up to AcOEt/MeOH 90:10); IR (KBr) ν_max_ 3304 (B–OH boronic acid; N–H amide), 2825 (C–H methylene), 1649 (C=O amide), 1589 (C=C aromatic), 1423 (C=C aromatic), 1365 (B–O boronic acid), 1222 (C–F allyl floride) cm^−1^. ^1^H-NMR (600 MHz, DMSO-*d*_6_) *δ*_H_ 10.30 (s, 1H, NH), 8.33 (m, H-2, carbamoyphenylboronic acid), 8.24 (s, 2H, OH), 7.98–7.97 (dd, *J* = 7.4, 1.2 Hz, H-6, carbamoyphenylboronic acid), 7.94–7.93 (m, H-4, carbamoyphenylboronic acid), 7.74–7.71 (dd, *J* = 15.0, 2.4 Hz, H-2, fluorophenyl), 7.51–7.50 (m, H-5, carbamoyphenylboronic acid), 7.50–7.48 (m, H-6, fluorophenyl), 7.11–7.09 (m, H-5, fluorophenyl), 7.07–7.05 (m, H-3, -5, fluorobenzene), 7.03–7.00 (dt, *J* = 7.1, 2.3 Hz, H-2, -6, fluorobenzene), 3.65–3.63 (m, H-2, -6, piperazine), 3.09–3.07 (m, H-3, -5, piperazine) ppm. ^13^C-NMR (150 MHz, DMSO-*d*_6_) *δ*_C_ 166.0 (1C, C=O), 156.9 (d, ^1^*J*_C–F_ = 236.6 Hz, C-4, fluorobenzene), 155.2 (d, ^1^*J*_C–F_ = 241.8 Hz, C-3, fluorophenyl), 147.9 (d, ^4^*J*_C–F_ = 2.1 Hz, C-1, fluorobenzene), 137.2 (C-6, carbamoyphenylboronic acid), 135.4 (d, ^2^*J*_C–F_ = 9.4 Hz, C-4, fluorophenyl), 134.5 (d, ^3^*J*_C–F_ = 11.8 Hz, C-1, fluorophenyl), 134.1 (C-3, carbamoyphenylboronic acid), 133.4 (C-2, carbamoyphenylboronic acid), 129.2 (C-4, carbamoyphenylboronic acid), 127.4 (C-5, carbamoyphenylboronic acid), 119.3 (d, ^3^*J*_C–F_ = 4.5 Hz, C-5, fluorophenyl), 117.4 (d, ^3^*J*_C–F_ = 7.9 Hz, C-2, -6, fluorobenzene),116.2 (d, ^4^*J*_C–F_ = 3.2 Hz, C-6, fluorophenyl), 115.3 (d, ^2^*J*_C–F_ = 21.7 Hz, C-3, -5, fluorobenzene), 108.4 (d, ^2^*J*_C–F_ = 24.6 Hz, C-2, fluorophenyl), 50.3 (C-3, -5, piperazine), 44.8 (C-2, -6, piperazine) ppm. HMRS (ESI^+^, methanol) C_23_H_23_BF_2_N_3_O_3_ [M+H]^+^ = 438.1796.

*{3-[(3-Fluoro-4-thiomorpholinophenyl)carbamoyl]phenyl}boronic acid* **32** white solid; yield 52%; mp: 116–122 °C (n-hexane/AcOEt/MeOH, gradient mode, starting with Hex/AcOEt 80:20 increasing the percentage of AcOEt by 10%, up to AcOEt/MeOH 90:10); IR (KBr) ν_max_ 3327 (N–H amide), 3268 (B–OH boronic acid), 2810 (C–H methylene), 1654 (C=O amide), 1589 (C=C aromatic), 1433 (C=C aromatic), 1319 (B–O boronic acid), 1249 (C–F allyl floride) cm^−1^. ^1^H-NMR (600 MHz, DMSO-*d*_6_) *δ*_H_ 10.28 (s, 1H, NH), 8.32 (m, H-2, carbamoyphenylboronic acid), 8.22 (s, 2H, OH), 7.98–7.96 (m, H-6, carbamoyphenylboronic acid), 7.94–7.93 (m, H-4, carbamoyphenylboronic acid), 7.71–7.69 (dd, *J* = 14.6, 2.3 Hz, H-2, fluorophenyl), 7.50–7.49 (m, H-5, carbamoyphenylboronic acid), 7.48–7.47 (m, H-6, fluorophenyl), 7.09–7.05 (t, *J* = 9.3 Hz, H-5, fluorophenyl), 3.28–3.20 (m, H-3, -5, thiomorpholine), 2.76–2.74 (m, H-2, -6, thiomorpholine) ppm. ^13^C-NMR (151 MHz, DMSO-*d*_6_) *δ*_C_ 166.0 (1C, C=O), 155.3 (d, ^1^*J*_C–F_ = 244.8 Hz, C-3, fluorophenyl), 137.1 (C-6, carbamoyphenylboronic acid), 136.4 (d, ^2^*J*_C–F_ = 9.9 Hz, C-4, fluorophenyl), 134.8 (d, ^3^*J*_C–F_ = 10.8 Hz, C-1, fluorophenyl), 134.1 (C-3, carbamoyphenylboronic acid), 133.4 (C-2, carbamoyphenylboronic acid), 129.1 (C-4, carbamoyphenylboronic acid), 127.4 (C-5, carbamoyphenylboronic acid), 120.4 (d, ^3^*J*_C–F_ = 3.8 Hz, C-5, fluorophenyl), 116.2 (d, ^4^*J*_C–F_ = 3.5 Hz, C-6, fluorophenyl), 108.4 (d, ^2^*J*_C–F_ = 25.8 Hz, C-2, fluorophenyl), 50.4 (C-2, -6, thiomorpholine), 49.4 (C-3, -5, thiomorpholine) ppm. HMRS (ESI^+^, methanol) C_17_H_19_BFN_2_O_3_S [M+H]^+^ = 361.1192.

*{3-[(3-Fluoro-4-morpholinophenyl)carbamoyl]phenyl}boronic acid* **33** white solid; yield 39%; mp: 248–256 °C (n-hexane/AcOEt/MeOH, gradient mode, starting with Hex/AcOEt 80:20 increasing the percentage of AcOEt by 10%, up to AcOEt/MeOH 90:10); IR (KBr) ν_max_ 3533 (N–H amide), 3298 (B–OH boronic acid), 2843 (C–H methylene), 1643 (C=O amide), 1589 (C=C aromatic), 1409 (C=C aromatic), 1315 (B–O boronic acid), 1255 (C–F allyl floride) cm^−1^. ^1^H-NMR (600 MHz, DMSO-*d*_6_) *δ*_H_ 10.28 (s, 1H, NH), 8.32 (m, H-2, carbamoyphenylboronic acid), 8.23 (s, 2H, OH), 7.98–7.96 (dt, *J* = 7.4, 1.1 Hz, H-6, carbamoyphenylboronic acid), 7.94–7.92 (m, H-4, carbamoyphenylboronic acid), 7.72–7.69 (dd, *J* = 15.0, 2.3 Hz, H-2, fluorophenyl), 7.50–7.49 (m, H-5, carbamoyphenylboronic acid), 7.49–7.48 (m, H-6, fluorophenyl), 7.05–7.02 (m, H-5, fluorophenyl), 3.75–3.73 (m, H-3, -5, morpholine), 2.98–2.96 (m, H-2, -6, morpholine) ppm. ^13^C-NMR (151 MHz, DMSO-*d*_6_) *δ*_C_ 166.0 (1C, C=O), 155.2 (d, ^1^*J*_C–F_ = 243.0 Hz, C-3, fluorophenyl), 137.2 (C-6, carbamoyphenylboronic acid), 135.5 (d, ^2^*J*_C–F_ = 9.5 Hz, C-4, fluorophenyl), 134.4 (d, ^3^*J*_C–F_ = 11.5 Hz, C-1, fluorophenyl), 134.1 (C-3, carbamoyphenylboronic acid), 133.4 (C-2, carbamoyphenylboronic acid), 129.1 (C-4, carbamoyphenylboronic acid), 127.4 (C-5, carbamoyphenylboronic acid), 119.0 (d, ^3^*J*_C–F_ = 4.2 Hz, C-5, fluorophenyl), 116.2 (d, ^4^*J*_C–F_ = 3.2 Hz, C-6, fluorophenyl), 108.4 (d, ^2^*J*_C–F_ = 24.9 Hz, C-2, fluorophenyl), 66.2 (C-3, -5, morpholine), 50.8 (C-2, -6, morpholine) ppm. HMRS (ESI^+^, methanol) C_17_H_19_BFN_2_O_4_ [M+H]^+^ = 345.1420.

*(3-{[4-(Azepan-1-yl)-3-fluorophenyl]carbamoyl}phenyl)boronic acid* **34** white solid; yield 32%; mp: 107–114 °C (n-hexane/AcOEt/MeOH, gradient mode, starting with Hex/AcOEt 80:20 increasing the percentage of AcOEt by 10%, up to AcOEt/MeOH 90:10); IR (KBr) ν_max_ 3495 (N–H amide), 3321 (B–OH boronic acid), 2924 (C–H methylene), 1643 (C=O amide), 1591 (C=C aromatic), 1425 (C=C aromatic), 1340 (B–O boronic acid), 1271 (C–F allyl floride) cm^−1^. ^1^H-NMR (600 MHz, DMSO-*d*_6_) *δ*_H_ 10.14 (s, 1H, NH), 8.31 (m, H-2, carbamoyphenylboronic acid), 8.21 (s, 2H, OH), 7.97–7.95 (dt, *J* = 7.5, 1.1 Hz, H-6, carbamoyphenylboronic acid), 7.93–7.91 (dt, *J* = 7.8, 1.1 Hz, H-4, carbamoyphenylboronic acid), 7.63–7.60 (dd, *J* = 16.2, 2.2 Hz, H-2, fluorophenyl), 7.49–7.46 (t, *J* = 7.5 Hz, H-5, carbamoyphenylboronic acid), 7.39–7.37 (dd, *J* = 9.0, 2.2 Hz, H-6, fluorophenyl), 6.92–6.89 (t, *J* = 10.4, 9.0 Hz, H-5, fluorophenyl), 3.20–3.28 (t, *J* = 5.7 Hz, H-2, -7, azepane), 1.75 (m, H-3, -6, azepane), 1.57–1.55 (t, *J* = 5.7, 2.6 Hz, H-4, -5, azepane) ppm. ^13^C-NMR (150 MHz, DMSO-*d*_6_) *δ*_C_ 165.7 (1C, C=O), 152.8 (d, ^1^*J*_C–F_ = 239.2 Hz, C-3, fluorophenyl), 137.0 (C-7, carbamoyphenylboronic acid), 135.6 (d, ^2^*J*_C–F_ = 9.8 Hz, C-4, fluorophenyl), 134.1 (C-3, carbamoyphenylboronic acid), 133.3 (C-2, carbamoyphenylboronic acid), 130.9 (d, ^3^*J*_C–F_ = 11.2 Hz, C-1, fluorophenyl), 129.0 (C-4, carbamoyphenylboronic acid), 127.4 (C-5, carbamoyphenylboronic acid), 117.1 (d, ^3^*J*_C–F_ = 5.8 Hz, C-5, fluorophenyl), 116.2 (d, ^4^*J*_C–F_ = 3.2 Hz, C-6, fluorophenyl), 108.8 (d, ^2^*J*_C–F_ = 26.6 Hz, C-2, fluorophenyl), 51.7 (C-2, -7, azepane), 28.5 (C-3, -6, azepane), 26.7 (C-4, -5, azepane). HMRS (ESI^+^, methanol) C_19_H_23_BFN2O_3_ [M+H]^+^ = 357.1787.

*[4-({3-Fluoro-4-[4-(pyridin-2-yl)piperazin-1-yl]phenyl}carbamoyl)phenyl]boronic acid* **35** white solid; yield 53%; mp: 198–204 °C (n-hexane/AcOEt/MeOH, gradient mode, starting with Hex/AcOEt 80:20 increasing the percentage of AcOEt by 10%, up to AcOEt/MeOH 90:10); IR (KBr) ν_max_ 3277 (B–OH boronic acid; N–H amide), 1643 (C=O amide), 1591 (C=C aromatic), 1444 (C=C aromatic), 1317 (B–O boronic acid), 1240 (C–F allyl floride) cm^−1^. ^1^H-NMR (600 MHz, DMSO-*d*_6_) *δ*_H_ 10.28 (s, 1H, NH), 8.25 (s, 2H, OH), 8.14–8.13 (dd, *J* = 4.6, 1.6 Hz, H-6, pyridine), 7.93–7.91 (m, H-3, -5, carbamoyphenylboronic acid), 7.90–7.89 (m, H-2, -6, carbamoyphenylboronic acid), 7.75–7.73 (dd, *J* = 14.9, 2.3 Hz, H-2, fluorophenyl), 7.56–7.54 (ddd, *J* = 8.6, 7.1, 2.0 Hz, H-4, pyridine), 7.51–7.49 (dd, *J* = 8.7, 1.8 Hz, H-6, fluorophenyl), 7.10–7.07 (m, H-5, fluorophenyl), 6.89–6.88 (d, *J* = 8.6 Hz, H-3, pyridine), 6.68–6.66 (m, H-5, pyridine), 3.65–3.66 (m, H-2, -6, piperazine), 3.09–3.07 (m, H-3, -5, piperazine) ppm. ^13^C-NMR (150 MHz, DMSO-*d*_6_) *δ*_C_ 165.5 (1C, C=O), 159.0 (C-2, pyridine), 155.2 (d, ^1^*J*_C–F_ = 248.2 Hz, C-3, fluorophenyl), 147.6 (C-6, pyperidine), 137.6 (C-4, pyperidine), 136.0 (C-4, carbamoyphenylboronic acid), 135.8 (d, ^3^*J*_C–F_ = 9.3 Hz, C-1, fluorophenyl), 134.4 (d, ^2^*J*_C–F_ = 11,0 Hz, C-4, fluorophenyl), 134.0 (C-3, -5, carbamoyphenylboronic acid), 126.5 (C-2, -4, carbamoyphenylboronic acid), 119.3 (d, ^3^*J*_C–F_ = 4.8 Hz, C-5, fluorophenyl), 116.3 (d, ^4^*J*_C–F_ = 2.8 Hz, C-6, fluorophenyl), 113.3 (C-2, pyridine), 108.4 (d, ^2^*J*_C–F_ = 25.2 Hz, C-2, fluorophenyl), 107.2 (C-3, pyridine), 50.3 (C-3, -5, piperazine), 44.8 (C-2, -6, piperazine) ppm. HMRS (ESI^+^, methanol) C_22_H_23_BFN_4_O_3_ [M+H]^+^ = 421.1840.

*[4-({3-Fluoro-4-[4-(pyrimidin-2-yl)piperazin-1-yl]phenyl}carbamoyl)phenyl]boronic acid* **36** white solid; yield 53%; mp: 214–220 °C (n-hexane/AcOEt/MeOH, gradient mode, starting with Hex/AcOEt 80:20 increasing the percentage of AcOEt by 10%, up to AcOEt/MeOH 90:10); IR (KBr) ν_max_ 3460 (N–H amide), 3286 (B–OH boronic acid), 2852 (C–H methylene), 1645 (C=O amide), 1591 (C=C aromatic), 1442 (C=C aromatic), 1317 (B–O boronic acid), 1251 (C–F allyl floride) cm^−1^. ^1^H-NMR (600 MHz, DMSO-*d*_6_) *δ*_H_ 10.27 (s, 1H, NH), 8.39–8.38 (ddd, *J* = 4.7 Hz, H-4, -6, pyrimidine), 8.24 (s, 2H, OH), 7.92–7.91 (m, H-3, -5, carbamoyphenylboronic acid), 7.90–7.88 (m, H-2, -6, carbamoyphenylboronic acid), 7.75–7.72 (dd, *J* = 14.9, 2.0 Hz, H-2, fluorophenyl), 7.50–7.48 (m, H-6, fluorophenyl), 7.09–7.07 (m, H-5, fluorophenyl), 6.66–6.65 (t, *J* = 4.7 Hz, H-5, pyrimidine), 3.90–3.89 (m, H-2, -6, piperazine), 3.05–3.03 (m, H-3, -5, piperazine) ppm. ^13^C-NMR (150 MHz, DMSO-*d*_6_) *δ*_H_ 165.5 (1C, C=O), 161.2 (C-2, pyrimidine), 158.0 (C-4, -6, pyrimidine), 155.2 (d, ^1^*J*_C–F_ = 242.8 Hz, C-3, fluorophenyl), 136.0 (C-4, carbamoyphenylboronic acid), 135.6 (d, ^3^*J*_C–F_ = 8.9 Hz, C-1, fluorophenyl), 134.5 (d, ^2^*J*_C–F_ = 10.8 Hz, C-4, fluorophenyl), 134.0 (C-3, -5, carbamoyphenylboronic acid), 126.5 (C-2, -6, carbamoyphenylboronic acid), 119.4 (d, ^3^*J*_C–F_ = 3.7 Hz, C-5, fluorophenyl), 116.3 (d, ^4^*J*_C–F_ = 2.4 Hz, C-6, fluorophenyl), 110.3 (C-5, pyrimidine), 108.4 (d, ^2^*J*_C–F_ = 25.5 Hz, C-2, fluorophenyl), 50.3 (C-3, -5, piperazine), 43.4 (C-2, -6, piperazine) ppm. HMRS (ESI^+^, methanol) C_21_H_22_BFN_5_O_3_ [M+H]^+^ = 422.1801.

*[4-({3-Fluoro-4-[4-(4-fluorophenyl)piperazin-1-yl]phenyl}carbamoyl)phenyl]boronic acid* **37** white solid; yield 51%; mp: 225–232 °C (n-hexane/AcOEt/MeOH, gradient mode, starting with Hex/AcOEt 80:20 increasing the percentage of AcOEt by 10%, up to AcOEt/MeOH 90:10); IR (KBr) ν_max_ 3304 (B–OH boronic acid; N–H amide), 2850 (C–H methylene), 1643 (C=O amide), 1589 (C=C aromatic), 1222 (C–F allyl floride) cm^−1^. ^1^H-NMR (600 MHz, DMSO-*d*_6_) *δ*_H_ 10.28 (s, 1H, NH), 8.24 (s, 2H, OH), 7.93–7.91 (m, H-3, -5, carbamoyphenylboronic acid), 7.90–7.88 (m, H-2, -6, carbamoyphenylboronic acid), 7.76–7.71 (dd, *J* = 15.0, 2.3 Hz, H-2, fluorophenyl), 7.52–7.50 (m, H-6, fluorophenyl), 7.12–7.09 (m, H-5, fluorophenyl), 7.08–7.06 (m, H-3, -5, fluorobenzene), 7.03–7.09 (m, H-H-2, -6, fluorobenzene), 3.25–3.23 (m, H-2, -6, piperazine), 3.15–3.12 (m, H-3, -5, piperazine) ppm. ^13^C-NMR (150 MHz, DMSO-*d*_6_) *δ*_C_ 165.5 (1C, C=O), 156.9 (d, ^1^*J*_C–F_ = 234.5 Hz, C-4, fluorobenzene), 155.2 (d, ^1^*J*_C–F_ = 243.3 Hz, C-3, fluorophenyl), 147.9 (C-1, fluorobenzene), 136.0 (C-4, carbamoyphenylboronic acid), 135.5 (d, ^3^*J*_C–F_ = 9.8 Hz, C-1, fluorophenyl), 134.4 (d, ^2^*J*_C–F_ = 11.0 Hz, C-4, fluorophenyl), 134.0 (C-3, -5, carbamoyphenylboronic acid), 126.5 (C-2, -6, carbamoyphenylboronic acid), 119.2 (d, ^3^*J*_C–F_ = 4.1 Hz, C-5, fluorophenyl), 117.4 (d, ^3^*J*_C–F_ = 8.6 Hz, C-2, -6, fluorobenzene), 116.3 (d, ^4^*J*_C–F_ = 2.9 Hz, C-6, fluorophenyl), 115.4 (d, ^2^*J*_C–F_ = 22.1 Hz, C-3, -5, fluorobenzene), 108.4 (d, ^2^*J*_C–F_ = 26.7 Hz, C-2, fluorophenyl), 50.4 (C-3, -5, piperazine), 49.3 (C-2, -6, piperazine) ppm. HMRS (ESI^+^, methanol) C_23_H_23_BF_2_N_3_O_3_ [M+H]^+^ = 438.1795.

*{4-[(3-Fluoro-4-thiomorpholinophenyl)carbamoyl]phenyl}boronic acid* **38** white solid; yield 47%; mp: 194–198 °C (n-hexane/AcOEt/MeOH, gradient mode, starting with Hex/AcOEt 80:20 increasing the percentage of AcOEt by 10%, up to AcOEt/MeOH 90:10); IR (KBr) ν_max_ 3302 (B–OH boronic acid; N–H amide), 2823 (C–H methylene), 1643 (C=O amide), 1589 (C=C aromatic), 1425 (C=C aromatic), 1327 (B–O boronic acid), 1284 (C–F allyl floride) cm^−1^. ^1^H-NMR (600 MHz, DMSO-*d6*) *δ*_H_ 10.27 (s, 1H, NH), 8.25 (s, 2H, OH), 7.92–7.91 (m, H-3, -5 carbamoyphenylboronic acid), 7.89–7.88 (m, H-2, -6, carbamoyphenylboronic acid), 7.72–7.69 (dd, *J* = 14.6, 2.2 Hz, H-2, fluorophenyl), 7.49–7.48 (m, H-6, fluorophenyl), 7.09–7.06 (m, H-5, fluorophenyl), 3.22–3.20 (m, H-3, -5, thiomorpholine), 3.15–3.12 (m, H-2, -6, thiomorpholine) ppm. ^13^C-NMR (150 MHz, DMSO-*d*_6_) *δ*_C_ 165.5 (1C, C=O), 155.3 (d, ^1^*J*_C–F_ = 242.1 Hz, C-3, fluorophenyl), 136.5 (d, ^3^*J*_C–F_ = 10.1 Hz, C-1, fluorophenyl), 135.9 (C-4, carbamoyphenylboronic acid), 134.6 (d, ^2^*J*_C–F_ = 11.0 Hz, C-4, fluorophenyl), 134.0 (C-3, -5, carbamoyphenylboronic acid), 126.5 (C-2, -6, carbamoyphenylboronic acid), 120.4 (d, ^3^*J*_C–F_ = 4.2 Hz, C-5, fluorophenyl), 116.3 (d, ^4^*J*_C–F_ = 2.8 Hz, C-6, fluorophenyl), 108.4 (d, ^2^*J*_C–F_ = 26.2 Hz, C-2, fluorophenyl), 50.1 (C-2, -6, thiomorpholine), 50.4 (C-3, -5, thiomorpholine) ppm. HMRS (ESI^+^, methanol) C_17_H_19_BFN_2_O_3_S [M+H]^+^ = 361.1184.

*{4-[(3-Fluoro-4-morpholinophenyl)carbamoyl]phenyl}boronic acid* **39** brown solid; yield 28%; mp: 206–214 °C (n-hexane/AcOEt/MeOH, gradient mode, starting with Hex/AcOEt 80:20 increasing the percentage of AcOEt by 10%, up to AcOEt/MeOH 90:10); IR (KBr) ν_max_ 3273 (B–OH boronic acid; N–H amide), 1647 (C=O amide), 1591 (C=C aromatic), 1440 (C=C aromatic), 1317 (B–O boronic acid), 1257 (C–F allyl floride) cm^−1^. ^1^H-NMR (600 MHz, DMSO-*d*_6_) *δ*_H_ 10.26 (s, 1H, NH), 8.24 (s, 2H, OH), 7.92–7.91 (m, H-3, -5, carbamoyphenylboronic acid), 7.89–7.88 (m, H-2, -6, carbamoyphenylboronic acid), 7.73–7.70 (dd, *J* = 15.0, 2.0 Hz, H-2, fluorophenyl), 7.50–7.48 (dd, *J* = 9.0, 2.0 Hz, H-6, flhorophenyl), 7.05–7.02 (t, *J* = 9.0 Hz, H-5, fluorophenyl), 3.75–3.73 (m, H-3, -5, morpholine), 2.98–2.96 (m, H-2, -6, morpholine) ppm. ^13^C-NMR (151 MHz, DMSO-*d*_6_) *δ*_C_ 165.4 (1C, C=O), 155.1 (d, ^1^*J*_C–F_ = 242.0 Hz, C-3, fluorophenyl), 136.0 (C-4, carbamoyphenylboronic acid), 135.5 (d, ^3^*J*_C–F_ = 10.5 Hz, C-1, fluorophenyl), 134.3 (d, ^2^*J*_C–F_ = 11.2 Hz, C-4, fluorophenyl), 134.0 (C-3, -5, carbamoyphenylboronic acid), 126.5 (C-2, -6, carbamoyphenylboronic acid), 118.9 (d, ^3^*J*_C–F_ = 4.4 Hz, C-5, fluorophenyl), 116.3 (d, ^4^*J*_C–F_ = 2.8 Hz, C-6, fluorophenyl), 108.5 (d, ^2^*J*_C–F_ = 26.1 Hz, C-2, fluorophenyl), 66.2 (C-3, -5, morpholine), 50.8 (C-2, -6, morpholine) ppm. HMRS (ESI^+^, methanol) C_17_H_19_BFN_2_O_4_ [M+H]^+^ = 345.1420.

*(4-{[4-(Azepan-1-yl)-3-fluorophenyl]carbamoyl}phenyl)boronic acid* **40** white solid; yield 62%; mp: 211–216 °C (n-hexane/AcOEt/MeOH, gradient mode, starting with Hex/AcOEt 80:20 increasing the percentage of AcOEt by 10%, up to AcOEt/MeOH 90:10); IR (KBr) ν_max_ 3321 (B–OH boronic acid; N–H amide), 2924 (C–H methylene), 1643 (C=O amide), 1427 (C=C aromatic), 1340 (B–O boronic acid), 1271 (C–F allyl floride) cm^−1^. ^1^H-NMR (600 MHz, DMSO-*d*_6_) *δ*_H_ 10.14 (s, 1H, NH), 8.24 (s, 2H, OH), 7.92–7.90 (m, H-3, -5, carbamoyphenylboronic acid), 7.89–7.87 (m, H-2, -6, carbamoyphenylboronic acid), 7.64–7.61 (dd, *J* = 16.2, 2.1 Hz, H-2, fluorophenyl), 7.40–7.38 (dd, *J* = 9.0, 2.1 Hz, H-6, fluorophenyl), 6.92–6.89 (t, *J* = 10.3, 9.0 Hz, H-5, fluorophenyl), 3.30–3.28 (m, H-2, -7, azepane), 1.75 (m, H-3, -6, azepane), 1.57–1.55 (m, H-4, -5, azepane) ppm. ^13^C-NMR (150 MHz, DMSO-*d*_6_) *δ*_C_ 165.1 (1C, C=O), 155.7 (d, ^1^*J*_C–F_ = 240.9 Hz, C-3, fluorophenyl), 136.1 (C-4, carbamoyphenylboronic acid), 135.7 (d, ^3^*J*_C–F_ = 9.4 Hz, C-1, fluorophenyl), 130.8 (d, ^2^*J*_C–F_ = 11.3 Hz, C-2, fluorophenyl), 126.5 (C-2, -6, carbamoyphenylboronic acid), 117.0 (d, ^3^*J*_C–F_ = 5.8 Hz, C-5, fluorophenyl), 116.5 (d, ^4^*J*_C–F_ = 1.9 Hz, C-6, fluorophenyl), 109.0 (d, ^2^*J*_C–F_ = 26.5 Hz, C-2, fluorophenyl), 51.7 (C-2, -7, azepane), 39.5 (C-3, -6, azepane), 26.7 (C-4, -5, azepane) ppm. HMRS (ESI^+^, methanol) C_19_H_23_BFN_2_O_3_ [M+H]^+^ = 357.1780.

E. General Miyaura borylation synthesis procedure **41** [25,26,27]

In a 25.0 mL flask, (0.65 mmol, 1.0 equiv.) methyl 4-bromo-3-methylbenzoate, (0.98 mmol, 1.5 equivalent) of bis(pinacolato)diboron (B_2_pin_2_), (1.95, 3.0 equivalent) of potassium acetate (KOAc), and (0.065 mmol, 10 mol%) of the catalyst were added. Then, [1,1′-bis(diphenylphosphino)ferrocene]palladium(II) dichloride [PdCl_2_(dppf)] was added to 10.0 mL of previously deaerated 1,4-dioxane, under a nitrogen atmosphere, at a temperature of 80 °C. After 12 h of reaction, the reaction mixture was filtered through celite 545, and the solvent was removed under reduced pressure; then, the resulting product was solubilized in dichloromethane, which was taken to a separatory funnel and washed with saturated sodium chloride solution (3 × 50.0 mL). The collected organic phase and the product were purified by column chromatography (eluent n-hexane/AcOEt, 9:1) [25,27].

*Methyl 3-methyl-4-(4*,*4*,*5*,*5-tetramethyl-1*,*3*,*2-dioxaborolan-2-yl)benzoate* **41** slightly yellow solid; yield 98%; (n-hexane/AcOEt, 9:1). ^1^H-NMR (300 MHz, DMSO-*d*_6_) *δ*_H_ 7.75–7.74 (m, H-2, -5, -6, methyl benzoate), 3.85 (s, 3H, CH_3_, methyl benzoate), 2.51 (s, 3H, CH_3_, methyl acetate), 1.31 (s, 12H, CH_3_, boronic ester) ppm.

F. General procedure of bromination synthesis **42** [27]

In a 25.0 mL flask, (0.54 mmol, 1.0 equiv.) of compound **41**, (0.54 mmol, 1.5 equiv.) of *N*-bromosuccinimide (NBS), and (0.09 mmol, 25 mol%) of 2,2′-azobis(2-methylpropionitrile) (AIBN) were added to 6.0 mL of anhydrous acetonitrile. The reaction was kept under stirring for 24 h at a temperature of 80 °C and a nitrogen atmosphere. After the reaction time, excess solvent was removed under reduced pressure, and the resulting product was solubilized in 50.0 mL of dichloromethane. The organic phase was washed with saturated NaHCO_3_ solution (3 × 50.0 mL), where the solvent was removed under reduced pressure, without the need to purify the final product [27].

*Methyl 3-(bromomethyl)-4-(4*,*4*,*5*,*5-tetramethyl-1*,*3*,*2-dioxaborolan-2-yl)benzoate* **42** colorless oil; yield 62%; ^1^H-NMR (300 MHz, DMSO-*d*_6_) *δ*_H_ 8.04–8.03 (d, *J* = 1.5 Hz, H-2, benzoate), 7.91–7.87 (dd, *J* = 7.8, 1.5 Hz, H-6, benzoate), 7.83–7.80 (d, *J* = 7.8 Hz, H-5, benzoate), 5.02 (s, 2H, CH_2_), 3.87 (s, 3H, CH_3_, methyl acetate), 1.34 (s, 12H, CH_3_, boronic ester).

G. General procedure for obtaining the benzoxaborole intermediate **43**

To a THF (2 mL)/NaOH aq (5 mL distilled water, 3.60 mmol, 10 equiv. NaOH) solution and under vigorous stirring, compound 42 (0.32 mmol, 1.0 equiv.) was added and the reaction mixture was heated at 50 °C for 2 h. Next, in the same conditions, a 6.0 M hydrochloric acid solution was added dropwise, until pH~2.0; stirring was continued for an additional 2 h at 50 °C. The reaction mixture as a solution was then cooled to room temperature and extracted with ethyl acetate (3 × 50 mL). The combined organic layers were dried over anhyd Na_2_SO_4_, filtered off, and evaporated under reduced pressure to dryness. The residue was purified by column chromatography in gradient mode, starting the mobile phase n-hexane/AcOEt (1:4 *v*/*v*), up to 95% EtOAc and 5% glacial acetic acid. [27].

*1-Hydroxy-1,3-dihydrobenzo[c][1,2]oxaborole-5-carboxylic acid* **43** white solid; yield 65%; (n-hexane/AcOEt (20:80), up to 95% EtOAc and 5% glacial acetic acid); IR (KBr) ν_max_ 3331 (O–H carboxylic acid), 3068 (B–OH benzoxaborole), 1693 (C=O carboxylic acid), 1489 (C=C aromatic), 1421 (C–H methylene), 1367 (B–O benzoxaborole) cm^−1^. ^1^H-NMR (600 MHz, DMSO-*d*_6_) *δ*_H_ 13.03 (s, 1H, OH, carboxylic acid), 9.39 (s, 1H, OH, oxaborole), 7.96 (m, H-4, benzoxaborole), 7.93–7.90 (d, *J* = 7.6 Hz, H-6, benzoxaborole), 7.84–7.81 (d, *J* = 7.6 Hz, H-7, benzoxaborole), 5.05 (s,H-3, oxaborole) ppm.

H. General procedure for obtaining benzoxaborole derivative **44** and **45**

For the synthesis of the benzoxaborole derivative **44** and **45**, (0.68 mmol, 1.0 equiv.) of the compound **43** and anhydrous *N’N*-diisopropylethylamine (DIPEA) in 5.0 mL of *N’N*-dimethylformamide were added to a 25.0 mL flask. (DMF) and stirred for 10 min at room temperature in a nitrogen atmosphere. After this time, (0.75 mmol, 1.1 equiv.) of the HATU coupling agent was added, and the reaction medium was stirred for another 2 h. Then, (0.68 mmol, 1.0 equiv.) of the compounds **19** and **22** was added, where the reaction remained under stirring for 24 h, at room temperature in a nitrogen atmosphere. The reaction was terminated with the addition of 50.0 mL of distilled water, in a separatory funnel, where it was extracted with ethyl acetate (3 × 50.0 mL); the organic phase was collected, dried with anhydrous sodium sulfate, and filtered, and the resulting solvent was removed under reduced pressure. The crude product was purified by column chromatography (eluent n-hexane/AcOEt 20:80) [50].

*N-{3-fluoro-4-[4-(pyridin-2-yl)piperazin-1-yl]phenyl}-1-hydroxy-1,3-dihydrobenzo[c][1,2]oxaborole-5-carboxamide* **44** light brown solid; yield 31%; mp: 211–216 °C (n-hexane/AcOEt 20:80) IR (KBr) ν_max_ 3327 (B–OH benzoxaborole), 1643 (C=O amide), 1593 (C=C aromatic), 1525 (N–H amide), 1421 (C=C aromatic), 1319 (C–F aryl fluoride) cm^−1^. ^1^H-NMR (600 MHz, DMSO-*d*_6_) *δ*_H_ 10.37 (s, 1H, NH), 9.39 (s, 1H, OH), 8.14–8.13 (dd, *J* = 4.8, 1.2 Hz, H-6, pyridine), 7.94 (m, H-4, benzoxaborole), 7.90–7.88 (d, *J* = 7.7 Hz, H-6, benzoxaborole), 7.86–7.85 (d, *J* = 7.7 Hz, H-7, benzoxaborole), 7.75–7.72 (dd, *J* = 14.9, 2.2 Hz, H-2, fluoropheyl), 7.57–7.54 (ddd, *J* = 8.9, 7.2, 2.0 Hz, H-4, pyridine), 7.51–7.49 (m, H-6, fluorophenyl), 7.11–7.07 (t, *J* = 9.3 Hz, H-5, fluorophenyl), 6.89–6.88 (d, *J* = 8.6 Hz, H-5, pyridine), 6.68–6.66 (dd, *J* = 7.0, 5.4 Hz, H-6, pyridine), 5.08 (s, H-3, benzoxaborole), 3.65–3.63 (m, H-2, -6, piperazine), 3.09–3.07 (m, H-3, -5, piperazine) ppm. ^13^C-NMR (150 MHz, DMSO-*d*_6_) *δ*_C_ 165.6 (1C, C=O), 159.0 (C-2, pyridine), 155.2 (d, ^1^*J*_C–F_ = 242.8 Hz, C-3, fluorophenyl), 154.0 (C-3a, benzoxaborole), 147.6 (C-6, pyridine), 137.6 (C-5, benzoxaborole), 136.9 (C-4, pyridine), 135.7 (d, ^2^*J*_C–F_ = 9.2 Hz, C-4, fluorophenyl), 134.3 (d, ^3^*J*_C–F_ = 10.9 Hz, C-1, fluorophenyl), 130.5 (C-7, benzoxaborole), 126.3 (C-6, benzoxaborole), 120.5 (C-4, benzoxaborole), 119.3 (d, ^3^*J*_C–F_ = 4.5 Hz, C-5, fluorophenyl), 116.3 (d, ^4^*J*_C–F_ = 3.3 Hz, C-6, fluorophenyl), 113.3 (C-5, pyridine), 108.4 (d, ^2^*J*_C–F_ = 25.7 Hz, C-2, fluorophenyl), 107.2 (C-3, pyridine), 69.9 (C-3, benzoxaborole), 50.2 (C-3, -5, piperazine), 44.8 (C-2, -6, piperazine) ppm. HRMS (ESI^+^, methanol) C_23_H_23_BF_4_O_3_ [M+H]^+^ = 433.1840.

*N-(3-fluoro-4-thiomorpholinophenyl)-1-hydroxy-1,3-dihydrobenzo[c][1,2]oxaborole-5-carboxamide* **45** brown oil; yield 15%; (n-hexane/AcOEt 20:80) IR (KBr) ν_max_ 3439 (B–OH benzoxaborole), 1647 (C=O amide), 1527 (N–H amide), 1423 (C=C aromatic), 1278 (C–F aryl fluoride) cm^−1^. ^1^H-NMR (600 MHz, DMSO-*d*_6_) *δ*_H_ 10.36 (s, 1H, NH), 9.37 (s, 1H, OH), 7.93 (m, H-4, benzoxaborole), 7.89–7.88 (d, *J* = 7.8 Hz, H-6, benzoxaborole), 7.86–7.85 (d, *J* = 7.8 Hz, H-7, benzoxaborole), 7.72–7.69 (dd, *J* = 14.6, 1.9 Hz, H-2, fluorophenyl), 7.48–7.46 (m, H-6, fluorophenyl), 7.09–7.06 (t, *J* = 9.3 Hz, H-5, fluorophenyl), 5.08 (s, H-2, benzoxaborole), 3.22–3.21 (m, H-3, -5, thiomorpholine), 2.76–2.74 (m, H-2, -6, thiomorpholine) ppm. ^13^C-NMR (150 MHz, DMSO-*d^6^*) *δ*_C_ 165.6 (1C, C=O), 155.3 (d, ^1^*J*_C–F_ = 242.9 Hz, C-3, fluorophenyl), 154.0 (C-3a, benzoxaborole), 136.9 (C-5, benzoxaborole), 136.6 (d, ^3^*J*_C–F_ = 9.6 Hz, C-1, fluorophenyl), 134.5 (d, ^2^*J*_C–F_ = 11.9 Hz, C-4, fluorophenyl), 130.5 (C-7, benzoxaborole), 126.3 (C-6, benzoxaborole), 120.4 (d, ^3^*J*_C–F_ = 4.0 Hz, C-5, fluorophenyl), 116.3 (d, ^4^*J*_C–F_ = 2.9 Hz, C-6, fluorophenyl), 108.5 (d, ^2^*J*_C–F_ = 26.5 Hz, C-2, fluorophenyl), 69.9 (C-3, benzoxaborole), 53.1 (C-3, -5, thiomorpholine), 27.3 (C-2, -6, thiomorpholine) ppm. HRMS (ESI^+^, methanol) C_23_H_23_BF_4_O_3_ [M+H]^+^ = 373.1199.

### 4.1. Biological Evaluation

#### 4.1.1. *Mycobacterium tuberculosis* Biological Assays

The biological tests were carried out in partnership with Prof. Dr. Fernando Rogério Pavan’s research group at the Prof. Dr. Hugo David Mycobacteria Laboratory, at the School of Pharmaceutical Sciences, São Paulo State University—Araraquara/Brazil

##### Determination of the Minimum Inhibitory Concentration (MIC_90_) Against Strains of *Mycobacterium tuberculosis* H_37_R_V_

The minimum inhibitory concentration (MIC_90_) was determined using the REMA (resazurin microtiter assay) methodology, in which stock solutions of the compounds were prepared in DMSO (10 mg/mL) and then diluted in Middlebrook 7H9 broth (Difco, New Jersey, USA) supplemented with OADC (0.09–25 μg/mL). Rifampicin and isoniazid (Sigma-Aldrich, St Louis, MO, USA) were used as standards, solubilized in DMSO and distilled water, respectively, at concentrations of 0.004–1.0 μg/mL. In the *M. tuberculosis* H_37_R_V_ strain suspension, 100 μL of inoculum was added to each well of the 96-well microplate, as well as 100 μL of the compounds at the concentrations described above. The plates were then incubated for seven days in an oven at 37 °C and 5% CO_2_. After this time, 30 μL of resazurin (Sigma-Aldrich, St Louis, MO, USA) was added. After the 24 h incubation period, the fluorescence of the wells was measured using Cytation 3 equipment Biotek^®^ (Agilent, Santa Clara, CA, USA). The MIC_90_ was determined as the lowest concentration of the compounds capable of inhibiting 90% of the bacillus’ growth [51].

##### Cytotoxicity Assay (IC_50_) and Selectivity Index (IS)

The cytotoxicity test was carried out on the most active compounds in the series, i.e., those with the lowest MIC_90_ values. For the test, MRC-5 (ATCC CCL-171) and J774A.1 (ATCC TIB-67) cells were used; they were cultured in a medium containing DNEM (Vitrocell^®^, Waldkirch, Germany) and RPMI (Vitrocell^®^, Waldkirch, Germany). Then, 10% fetal bovine serum, gentamicin sulfate (50 mg/mL) and amphotericin B (2 mg/L) were added. The cells were incubated in an oven at 37 °C with 5% CO_2_, and a specific amount of each cell type (2.5 × 10^5^ cells/mL for MRC-5 and 1.0 × 106 for J774A.1) was distributed in a 96-well microplate, with a total volume of 100 μL. They were then incubated for 24 h at 37 °C in an atmosphere containing 5% CO_2_. The compounds were then added to the wells, at dilutions of 0.39–100 μg/mL, and incubated again for a further 24 h. After this interval, 50 μL of 0.01% resazurin solution was added, and after 3 h, the fluorescence of the wells was measured using a Synergy H1 reader Biotek^®^ (Agilent, Santa Clara, CA, USA). To determine the IC_50_ values, those concentrations capable of maintaining the viability of 50% of the cells were characterized. The selectivity index (SI) was determined by the ratio between the IC_50_ and MIC_90_ values (SI = IC_50_/MIC_90_). Compounds with an IS greater than or equal to 10 are considered selective, meaning that the compound is 10 times more toxic to the *M. tuberculosis* strain than to eukaryotic cells [52,53].

#### 4.1.2. Antifungal Assays

##### Microorganisms and Culture Conditions

This research used strains of *Candida albicans* ATCC 90028, *T. rubrum* ATCC 28189, and *T. mentagrophytes* 11481 clinical isolates. The ATCC strains were obtained from the library of the Mycology Laboratory of the Clinical Analysis Department of the School of Pharmaceutical Sciences, UNESP, Brazil.

*Candida albicans* were maintained on Sabouraud agar for 24 h at 35 °C, according to the norms stipulated by the Clinical and Laboratory Standards Institute (CLSI) M27-A3 protocol. *T. rubrum* and T mentagrophytes were maintained on Sabouraud agar and incubated at 28 °C for 7 to 15 days. Then, the strain was cultivated on malt extract agar [malt extract (Kasvi) 2%, peptone from animal tissue (Sigma-Aldrich, St Louis, MO, USA) 2%, glucose (Synth, São Paulo, Brazil) 2%, and agar (Kasvi, Paraná, Brazil) 2%], pH 5.7, and kept at 25 °C for 7 days or until sporulation [54,55].

##### Determination of Minimum Inhibitory Concentration (MIC)

The susceptibility tests were carried out according to the Clinical and Laboratory Standards Institute (CLSI) M27-A3 for *C. albicans* and to the document M38-A2 for *T. rubrum* and *T. mentagrophytes* [56,57].

Briefly, the compounds were solubilized in 100% dimethyl sulfoxide (DMSO) at a stock concentration of 30,000 mg/L and stored at −80 °C. The working solution of the compounds was prepared in Roswell Park Memorial Institute (RPMI-1640) with L-glucose, without sodium bicarbonate, and with phenol red as the pH indicator (Gibco, New York, USA) and buffered with 4-Morpholinepropanesulfonic acid hemisodium salt (MOPS) (Sigma-Aldrich, St Louis, MO, USA), pH = 7, at concentrations ranging from 0.24 to 125 mg/L. Terbinafine (Sigma-Aldrich, St Louis, MO, USA) (0.001 to 0.5 mg/L) and fluconazole) (0.12 to 64 mg/L) were used for quality control.

For the establishment of the MIC, an initial inoculum of *C. albicans* was prepared in saline 0.85% NaCl containing 1 to 5 × 10^6^ cells/mL; from this inoculum, two dilutions of 1:100 and 1:20 were subsequently made in RPMI 1640, so that the final concentration of cells was from 5 × 10^2^ to 2.5 × 10^3^ cells/mL. For dermatophytes, fungal suspensions were prepared in 0.85% NaCl and conidia, adjusted to a final concentration of 2.5 × 10^4^ cells/mL using a hemocytometer, and then added to microdilution plates. The volume of 100 µL of the dilutions of the compounds and antifungal drugs was added to a 96-well microplate following 100 µL of the inoculum. The MIC was stipulated using visual and colorimetric readings by adding 30 µL of 0.03% resazurin [56,57,58].

##### Determination of the Minimum Fungicide Concentration (MFC)

Briefly, aliquots of 100 µL from each well from the MIC assay were added to Sabouraud dextrose agar (BD Difco TM, New Jersey, USA) in Petri dishes and incubated for 96 h at 28 °C. The MFC was defined as the lowest concentration of the compound capable of no growth of fungal colonies [56].

##### Cytotoxicity Assay

For the cytotoxicity assay, the HaCat cell line, characterized as immortalized normal human keratinocytes, was used. The cells were maintained in (DMEM) supplemented with 10% of bovine fetal serum (BFS) (Sigma-Aldrich, St Louis, MO, USA) in cell culture bottles with 5% CO_2_ at 37 °C. After a confluence of 75%, the cells were treated with trypsin + EDTA 0.25%, centrifugated at 1500 rpm for 10 min, and 5 × 10^4^ cells/well were seeded in 96-well plates for incubation for 24 h for cell adhesion before treatment. After adhesion, the cells were treated with 100 µL of the compounds **34** and **40**, with fungi activity in concentrations between 3.90 and 125 µg/mL. Regarding the controls, DMSO in the concentration range of (1–4%) was used as a positive control for cytotoxicity; the beyond negative control comprised HaCaT cells only in DMEM medium (Sigma-Aldrich, St Louis, MO, USA). After 24 h of treatment, 30 µL of resazurin 50 µM was added to each well for 4 h, and the plates were read at 560 and 600 nm using microplate reader BioTek (Agilent, Santa Clara, CA, USA), Gen 5 software (version 2.09). The minimum inhibitory concentration (IC_50_) for each compound was determined based on resazurin reduction. Three independent assays were performed [59].

#### 4.1.3. Anticancer Assays

The SCC-25 was purchased from Rio de Janeiro Cell Bank, and NOK-si was kindly provided by Prof. Carlos Rossa from the School of Dentistry—UNESP. The cell lines were initially thawed and cultured in 25 cm^2^ cell culture flasks. Cell growth was monitored using a microscope, and the culture medium was changed periodically. Upon reaching confluency, cell dissociation was achieved by adding 5 mL of trypsin (Sigma-Aldrich, St Louis, MO, USA) and incubating the bottles in a CO_2_ incubator for 10 min. Subsequently, the cells were transferred to 15 mL Falcon tubes and centrifuged at 2000 rpm for 10 min. Cell counting was performed using a Countess II FL automated cell counter (ThermoFischer, Waltham, MA, USA). The cells were seeded at a density of 30,000 cells per well in 96-well plates. The plates were then incubated at 37 °C in 5% CO_2_ for 24 h. Following this, test compounds were incubated for another 24 h at 10 different concentrations: 50, 25, 12.5, 6.25, 3.12, 1.56, 0.78, 0.39, 0.19, and 0.097 µg/mL. After the specified time, MTT reagent (3 mg/mL) (Sigma-Aldrich, St Louis, MO, USA) dissolved in phenol-free RPMI 1640 was added and incubated for 4 h. The formed MTT crystals were solubilized using 2-propanol P.A (Sigma-Aldrich, St Louis, MO, USA) with agitation. Absorbance was read at 562 nm using an EZ Read 400 microplate reader (Biochrom, Cambridge, UK) and ADAP 2.0 Biochrom software (version 2.0). IC_50_ values and graphs were calculated by Dr. Paula Aboud Barbugli using Excel and Prism software (version 10.4.1). The MTT assay results were evaluated for normality using the Shapiro–Wilk test, followed by one-way ANOVA with Tukey’s post hoc test at a significance level of 5%.

## 5. Conclusions

The objective of this study was to develop and characterize novel boronic acid and benzoboroxole compounds due to their potential as antimycobacterial, antifungal, and antitumor agents. Fourteen new derivatives were synthesized and evaluated for biological activity. While none of the compounds exhibited significant antimycobacterial properties against *Mycobacterium tuberculosis* (H_37_R_v_), one derivative, **34**, demonstrated moderate antifungal activity against dermatophyte fungi, *T. rubrum*, and *T. mentagrophytes* (MIC_90_ = 42.0 μM and 21.0 μM, respectively). Furthermore, two compounds, **31** and **35**, exhibited promising antitumor activity against head and neck cancer cell lines, demonstrating selectivity with IC_50_ values of 59.07 μM and 45.61 μM, respectively. Based on these findings, these compounds may represent promising candidates for further exploration as potential therapeutic agents for head and neck cancer. However, additional research is necessary to elucidate their mechanism of action and to evaluate their efficacy in clinical trials.

## Figures and Tables

**Table 1:** Antimicrobial and cLogD *.

Compounds	*T. rubrum* ATCC 28189 MIC_90_ (μM)	*T. mentagrophytes* ATCC 28189 MIC_90_ (μM)	*C. albicans* ATCC 90028 MIC_90_ (μM)	*M. tuberculosis* H_37_R_v_ MIC_90_ (μM)	cLogD *
**29**	297	297	297	>25	4.44
**30**	297	297	297	>25	3.60
**31**	286	286	286	>25	5.19
**32**	347	347	347	>25	3.34
**33**	363	363	363	>25	2.76
**34**	42.0	21.0	351	>25	4.22
**35**	297	297	297	>25	4.44
**36**	297	297	297	>25	3.60
**37**	286	286	286	>25	5.19
**38**	347	347	347	>25	3.34
**39**	363	363	363	>25	2.76
**40**	88.0	42.0	351	>25	4.22
**44**	289	146	289	>25	4.90
**45**	336	336	336	>25	3.80
Terbinafine	0.10 nM	0.025 nM	–	–	–
Itraconazole	–	–	0.34 nM	–	–
Isoniazide	–	–	–	0.11	–

* cLogD calculated using ChemAxon’s calculator.

**Table 2:** Anticancer activity.

Compounds	SCC-25 ** IC_50_ (μM)	NOK-si *** IC_50_ (μM)	SI
**29**	117.4	110.0	<1
**30**	N/D *	N/D *	<1
**31**	59.07	220.7	3.73
**32**	N/D *	N/D *	<1
**33**	N/D *	N/D *	<1
**34**	67.57	129.3	1.91
**35**	45.61	153.7	3.37
**36**	133.4	133.5	1.00
**37**	79.06	123.0	1.55
**38**	N/D *	149.0	<1
**39**	74.09	77.09	1.04
**40**	85.09	127.2	1.49
**44**	N/D *	N/D *	<1
**45**	82.98	65.77	<1
5-fluorouracil	1800	–	–

* N/D: not determined. ** SCC-25: squamous cell carcinoma. *** NOK-si: normal oral keratinocytes–spontaneously immortalized.

## Data Availability

Data are contained within the article and Supplementary Materials.

## Supplementary Materials

The following supporting information can be downloaded at: https://www.mdpi.com/article/10.3390/molecules30051117/s1, Figures S1–S12: Spectra of compounds **13**–**15** and **19**–**21** (^1^H, ^13^C NMR), Figures S13–S100: Spectra of compounds **29**–**45** (^1^H NMR, ^13^C NMR, HRMS/ESI, IR). Detailed ^1^H NMR spectral data for compounds **16**–**18** and **22**–**24** is reported in reference [19].
