# Supplementary material for: Synthesis and Evaluation of Boron-Containing Heterocyclic Compounds with Antimicrobial and Anticancer Activities
Source: Molecules. 2025 Feb 28;30(5):1117. doi: 10.3390/molecules30051117 (PMC11901438; doi:10.3390/molecules30051117)
Supplement: Supplementary file 1 [file molecules-30-01117-s001.zip › molecules-3450689-supplementary.pdf]

Synthesis and Evaluation of Boron-Containing Heterocyclic Compounds with Antimicrobial and Anticancer Activities

João Lucas Bruno Prates [1,2,*], Samanta de Matos Silva [1], Kaila Petrolina Medina-Alarcón [1], Kelvin Sousa dos Santos [1], Jenyffie Araujo Belizario [1], Juliana Romano Lopes [1], Freddy Humberto Marin-Dett [1], Debora Leite Campos [1], Maria José Soares Mendes Giannini [1], Ana Marisa Fusco-Almeida [1], Paula Aboud Barbugli [1,3], Fernando Rogério Pavan [1] and Jean Leandro Dos Santos [1,2,*]

[1] School of Pharmaceutical Sciences, São Paulo State University (UNESP), Araraquara 14800-903, SP, Brazil; samanta.matos@unesp.br (S.d.M.S.); kaila.medina@unesp.br (K.P.M.-A.); k.santos@unesp.br (K.S.d.S.); jenyffie.belizario@unesp.br (J.A.B.); romano.lopes@unesp.br (J.R.L.); leite.debora26@gmail.com (D.L.C.); maria.giannini@unesp.br (M.J.S.M.G.); ana.marisa@unesp.br (A.M.F.-A.), fernando.pavan@unesp.br (F.R.P.)
[2] Institute of Chemistry, São Paulo State University (UNESP), Araraquara 14800-900, SP, Brazil
[3] School of Dentistry, São Paulo State University (UNESP), Araraquara 14801-385, SP, Brazil; freddy.m.dett@unesp.br (F.H.M.-D.); paula.barbugli@unesp.br (P.A.B.)
* Correspondence: joao.prates@unesp.br (J.L.B.P.); jean.santos@unesp.br (J.L.D.S.); Tel.: +55-16-3301-6962 (J.L.D.S.)

Table of contents	Figure	Page
^{1}H, ^{13}C NMR compound **13**	Figure S1-S2	3
^{1}H, ^{13}C NMR compound **14**	Figure S3-S4	4
^{1}H, ^{13}C NMR compound **15**	Figure S5-S6	5
^{1}H, ^{13}C NMR compound **19**	Figure S7-S8	6
^{1}H, ^{13}C NMR compound **20**	Figure S9-S10	7
^{1}H, ^{13}C NMR compound **21**	Figure S11-S12	8
IR, HRMS compound **29**	Figure S13-S14	9
^{1}H, ^{13}C NMR compound **29**	Figure S15-S16	10
HSQC, HMBC NMR compound **29**	Figure S17-S18	11
IR, HRMS compound **30**	Figure S19-S20	12
^{1}H, ^{13}C NMR compound **30**	Figure S21-S22	13
HSQC, HMBC NMR compound **30**	Figure S23-S24	14
IR, HRMS compound **31**	Figure S25-S26	15
^{1}H, ^{13}C NMR compound **31**	Figure S27-S28	16
HSQC, HMBC NMR compound **31**	Figure S29-S30	17
IR, HRMS compound **32**	Figure S31-S32	18
^{1}H, ^{13}C NMR compound **32**	Figure S33-S34	19
HSQC, HMBC NMR compound **32**	Figure S35-S36	20
IR, HRMS compound **33**	Figure S37-S38	21
^{1}H, ^{13}C NMR compound **33**	Figure S39-S40	22

HSQC, HMBC NMR compound **33**	Figure S41-S42	23
IR, HRMS compound **34**	Figure S43-S44	24
^{1}H, ^{13}C NMR compound **34**	Figure S45-S46	25
HSQC, HMBC NMR compound **34**	Figure S47-S48	26
IR, HRMS compound **35**	Figure S49-S50	27
^{1}H, ^{13}C NMR compound **35**	Figure S51-S52	28
HSQC, HMBC NMR compound **35**	Figure S53-S54	29
IR, HRMS compound **36**	Figure S55-S56	30
^{1}H, ^{13}C NMR compound **36**	Figure S57-S58	31
HSQC, HMBC NMR compound **36**	Figure S59-S60	32
IR, HRMS compound **37**	Figure S61-S62	33
^{1}H, ^{13}C NMR compound **37**	Figure S63-S64	34
HSQC, HMBC NMR compound **37**	Figure S65-S66	35
IR, HRMS compound **38**	Figure S67-S68	36
^{1}H, ^{13}C NMR compound **38**	Figure S69-S70	37
HSQC, HMBC NMR compound **38**	Figure S71-S72	38
IR, HRMS compound **39**	Figure S73-S74	39
^{1}H, ^{13}C NMR compound **39**	Figure S75-S76	40
HSQC, HMBC NMR compound **39**	Figure S77-S78	41
IR, HRMS compound **40**	Figure S79-S80	42
^{1}H, ^{13}C NMR compound **40**	Figure S81-S82	43
HSQC, HMBC NMR compound **40**	Figure S83-S84	44
^{1}H NMR compounds **41** and **42**	Figure S85-S86	45
IR, ^{1}H NMR compound **43**	Figure S87-S88	46
IR, HRMS compound **44**	Figure S89-S90	47
^{1}H, ^{13}C NMR compound **44**	Figure S91-S92	48
HSQC, HMBC NMR compound **44**	Figure S93-S94	49
IR, HRMS compound **45**	Figure S95-S96	50
^{1}H, ^{13}C NMR compound **45**	Figure S97-S98	51
HSQC, HMBC NMR compound **45**	Figure S99-S100	52

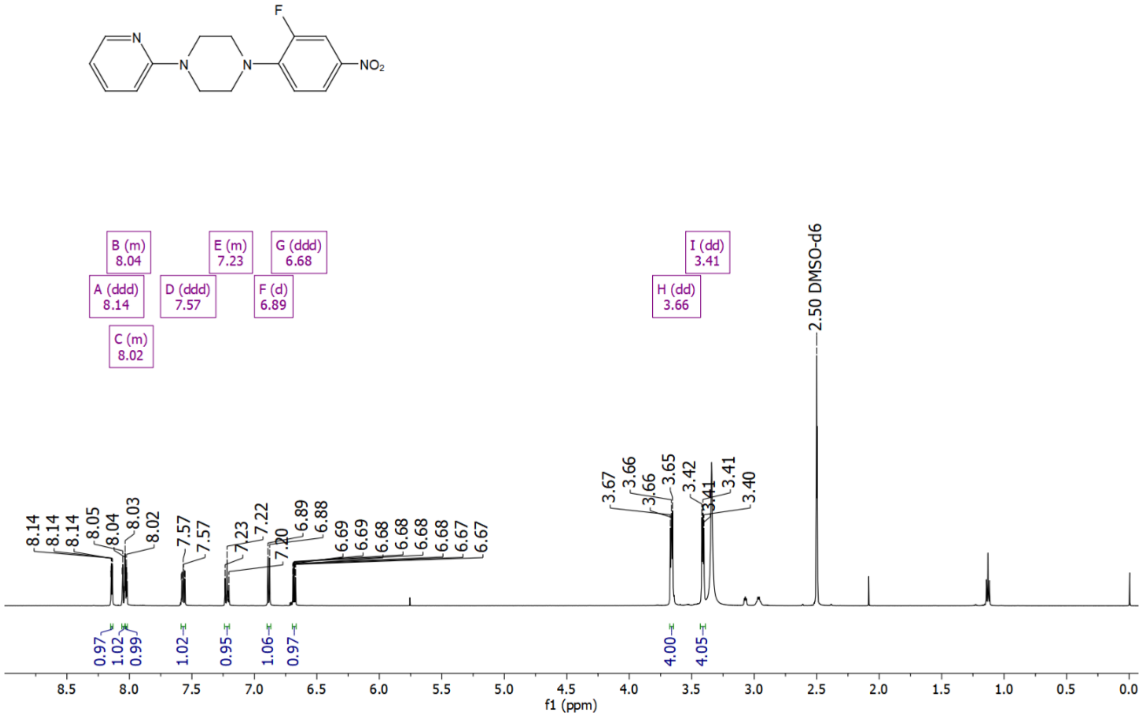

Figure S1: ^{1}H-NMR Spectrum of compound **13** (600 MHz DMSO-*d*$_6$).

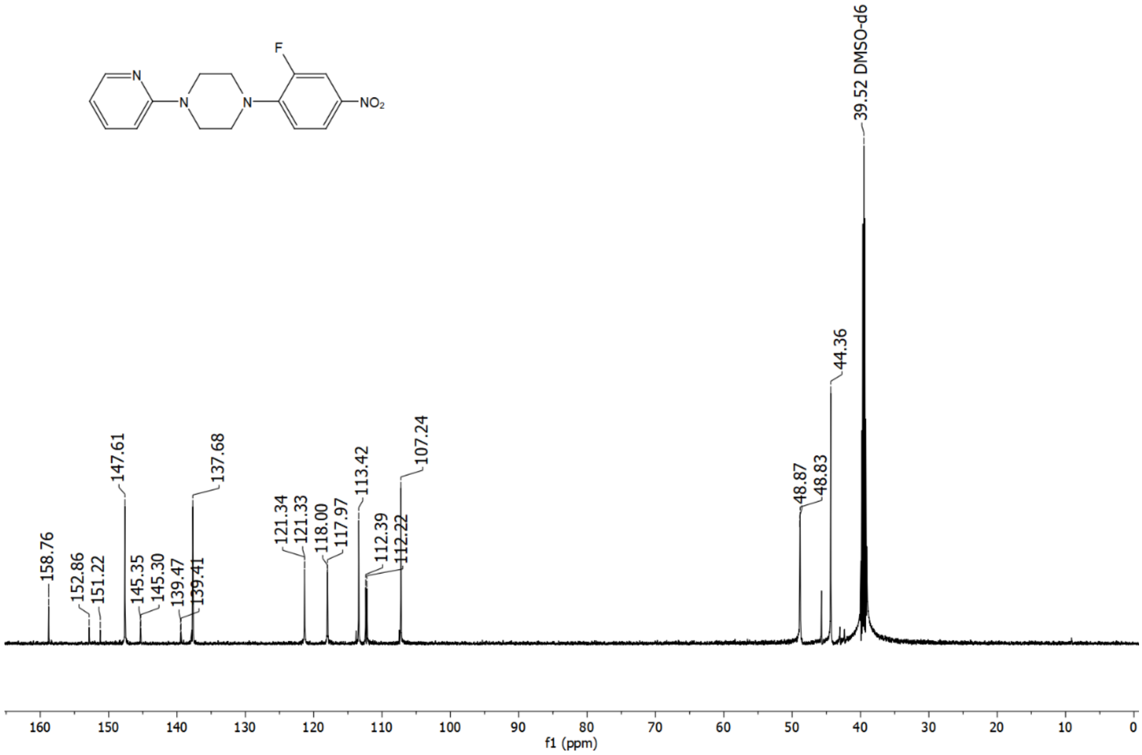

Figure S2: ^{13}C-NMR Spectrum of compound **13** (150 MHz DMSO-*d*$_6$).

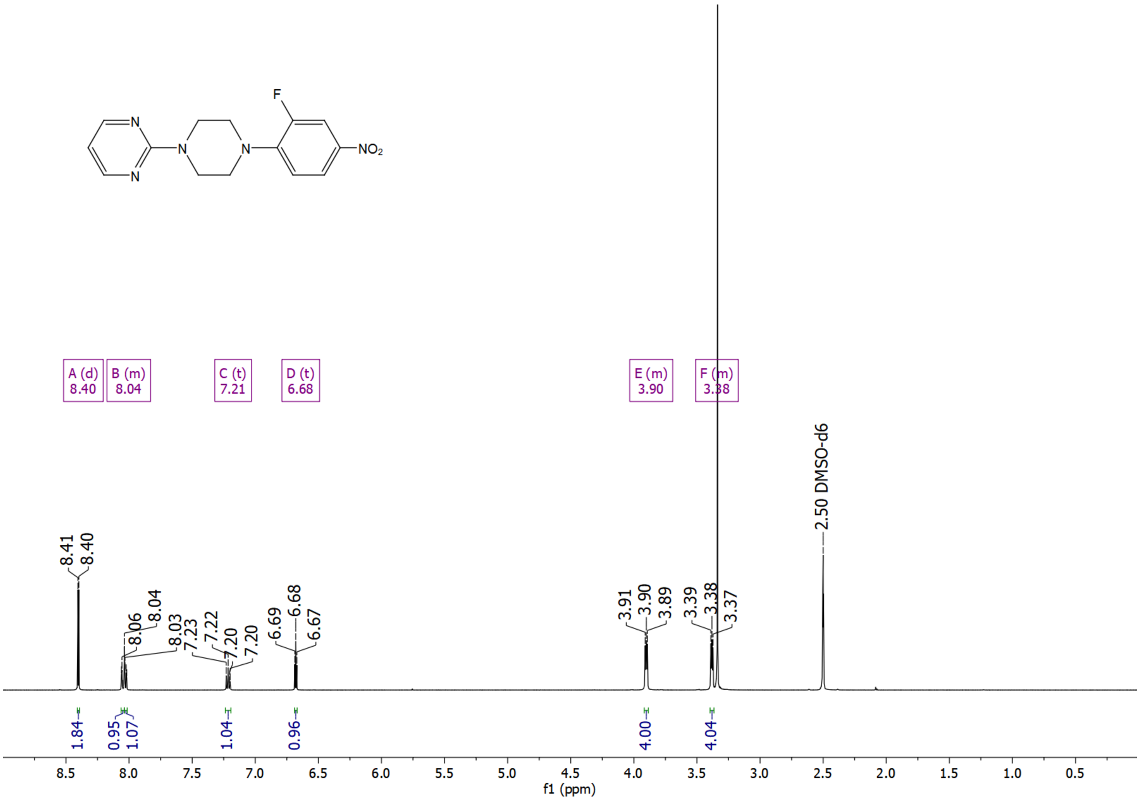

Figure S3: ^{1}H-NMR Spectrum of compound **14** (600 MHz DMSO-d_6).

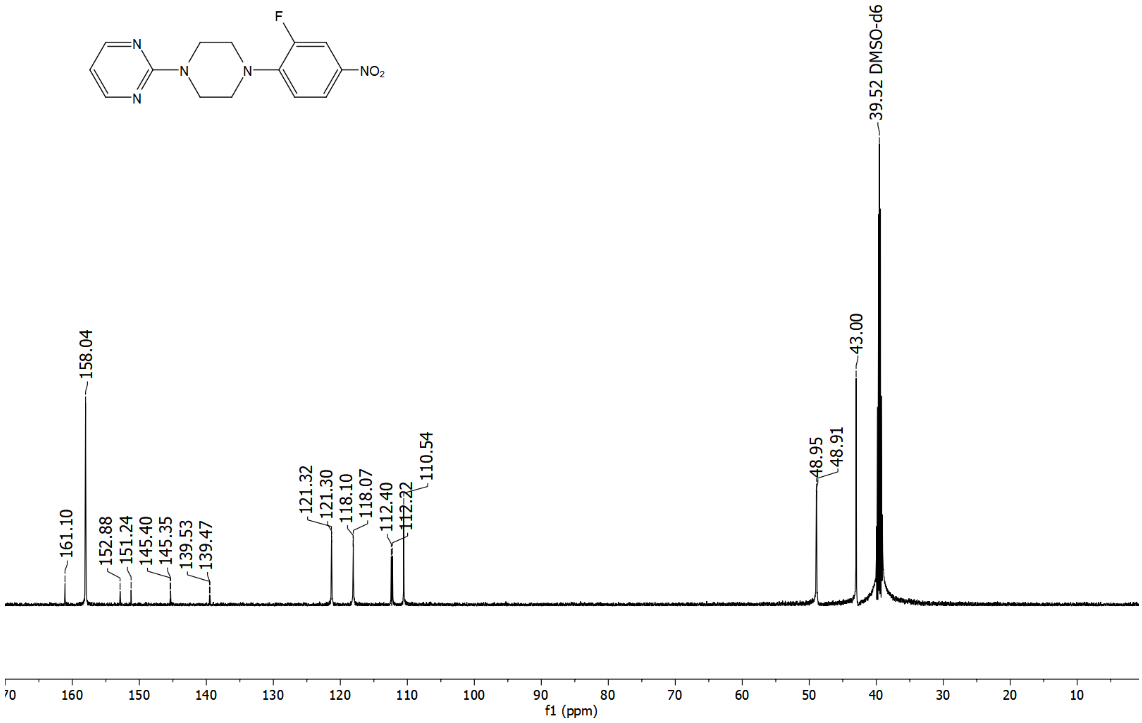

Figure S4: ^{13}C-NMR Spectrum of compound **14** (150 MHz DMSO-d_6).

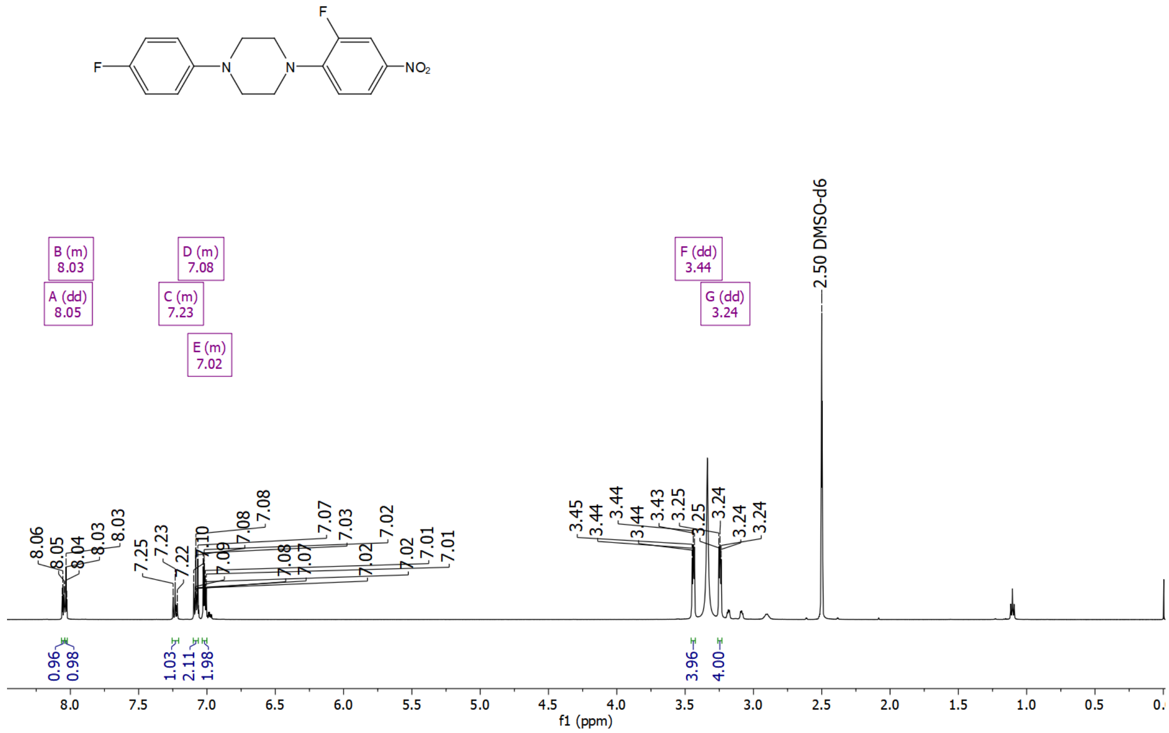

Figure S5: ^{1}H-NMR Spectrum of compound **15** (600 MHz DMSO-d_6).

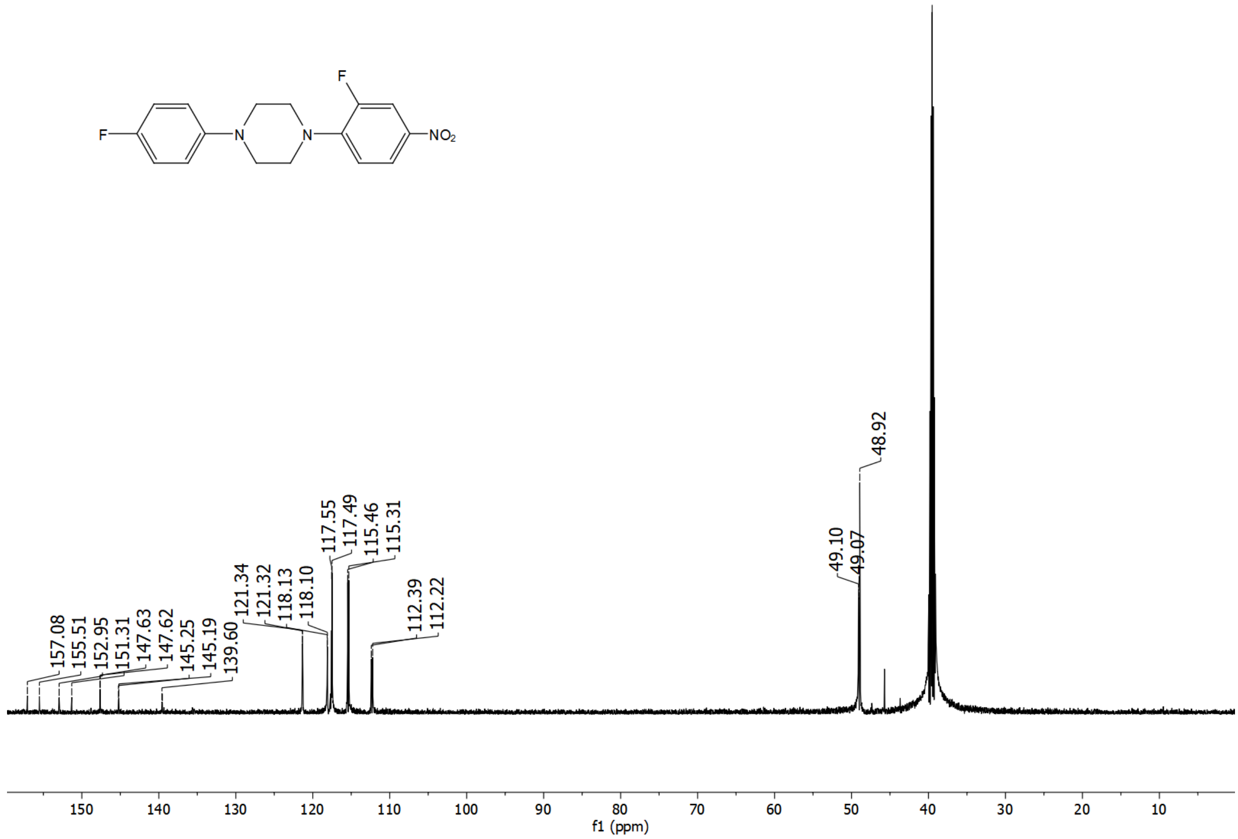

Figure S6: ^{13}C-NMR Spectrum of compound **15** (150 MHz DMSO-d_6).

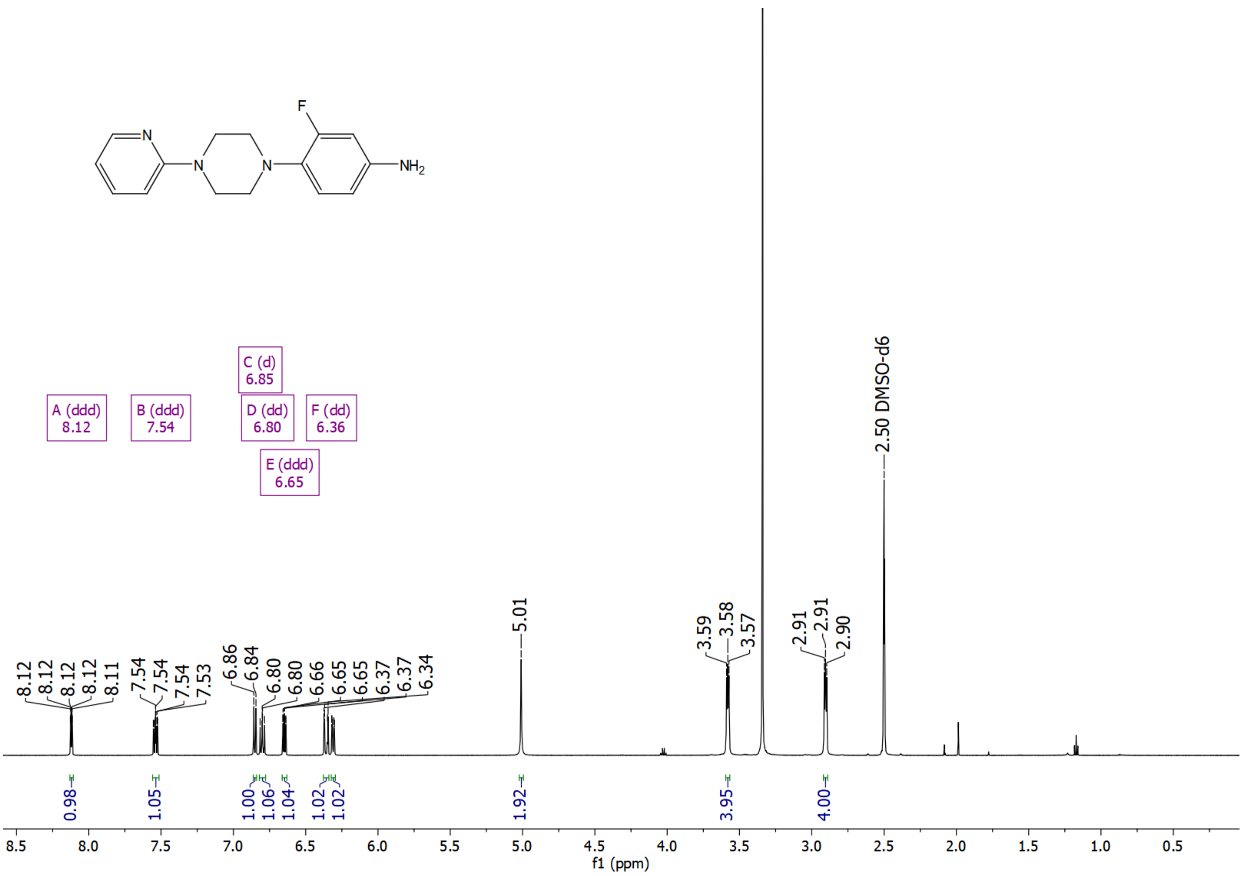

Figure S7: ^{1}H-NMR Spectrum of compound **19** (600 MHz DMSO-d_6).

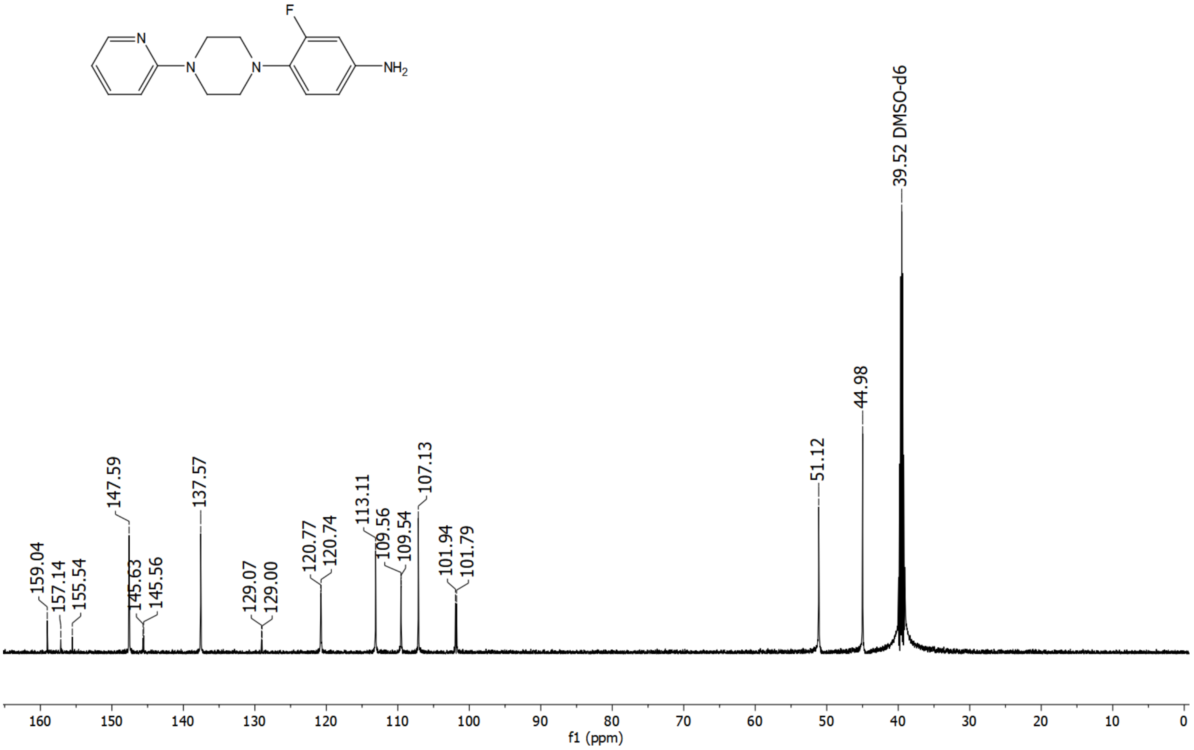

Figure S8: ^{13}C-NMR Spectrum of compound **19** (150 MHz DMSO-d_6).

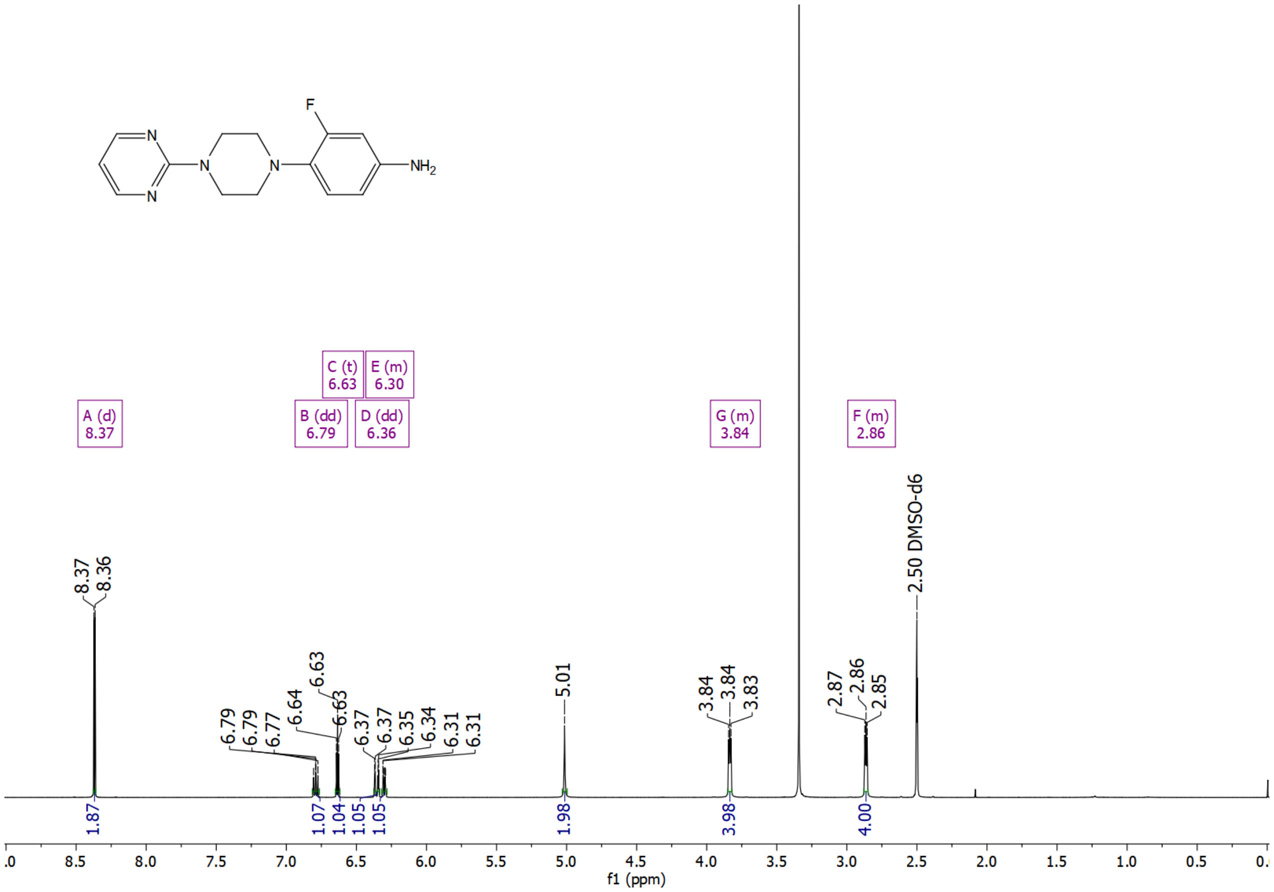

Figure S9: ^{1}H-NMR Spectrum of compound **20** (600 MHz DMSO-d_6).

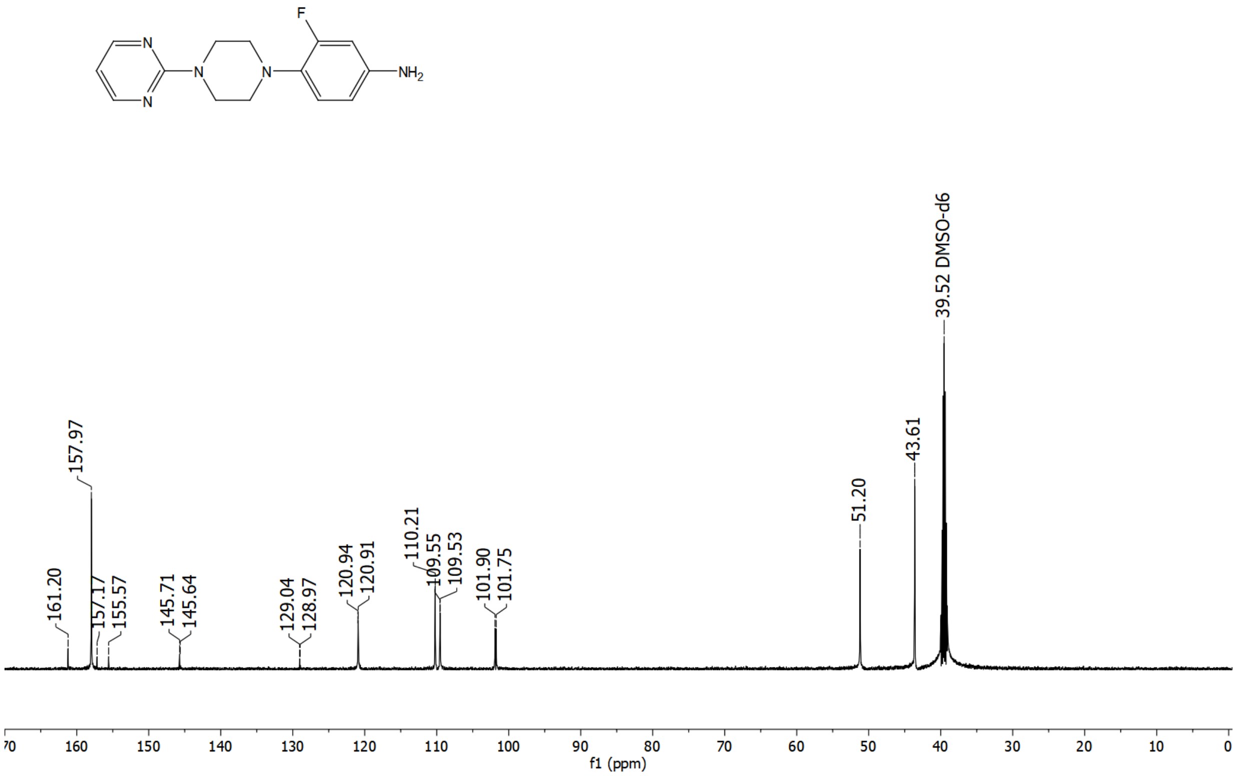

Figure S10: ^{13}C-NMR Spectrum of compound **20** (150 MHz DMSO-d_6).

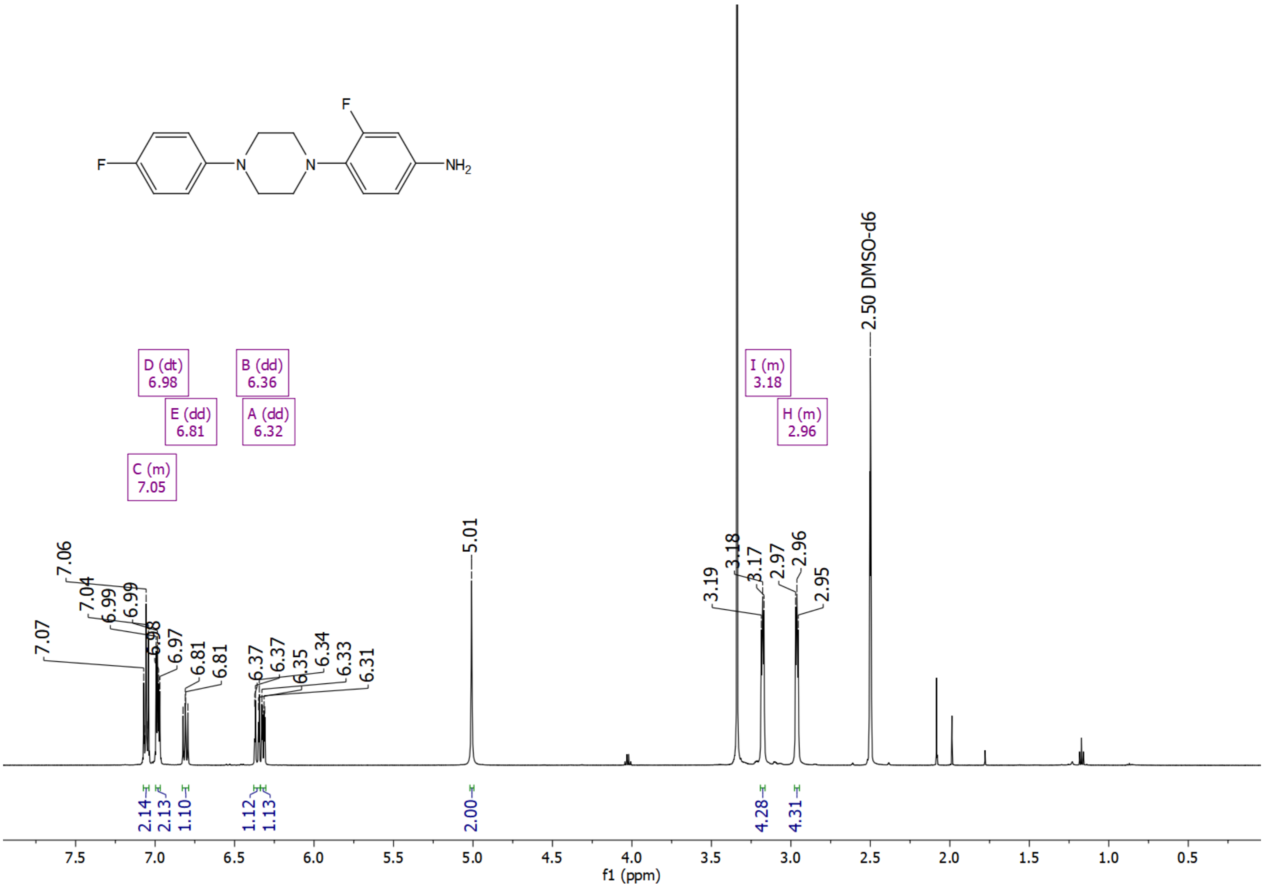

Figure S11: ^{1}H-NMR Spectrum of compound **21** (600 MHz DMSO-d_6).

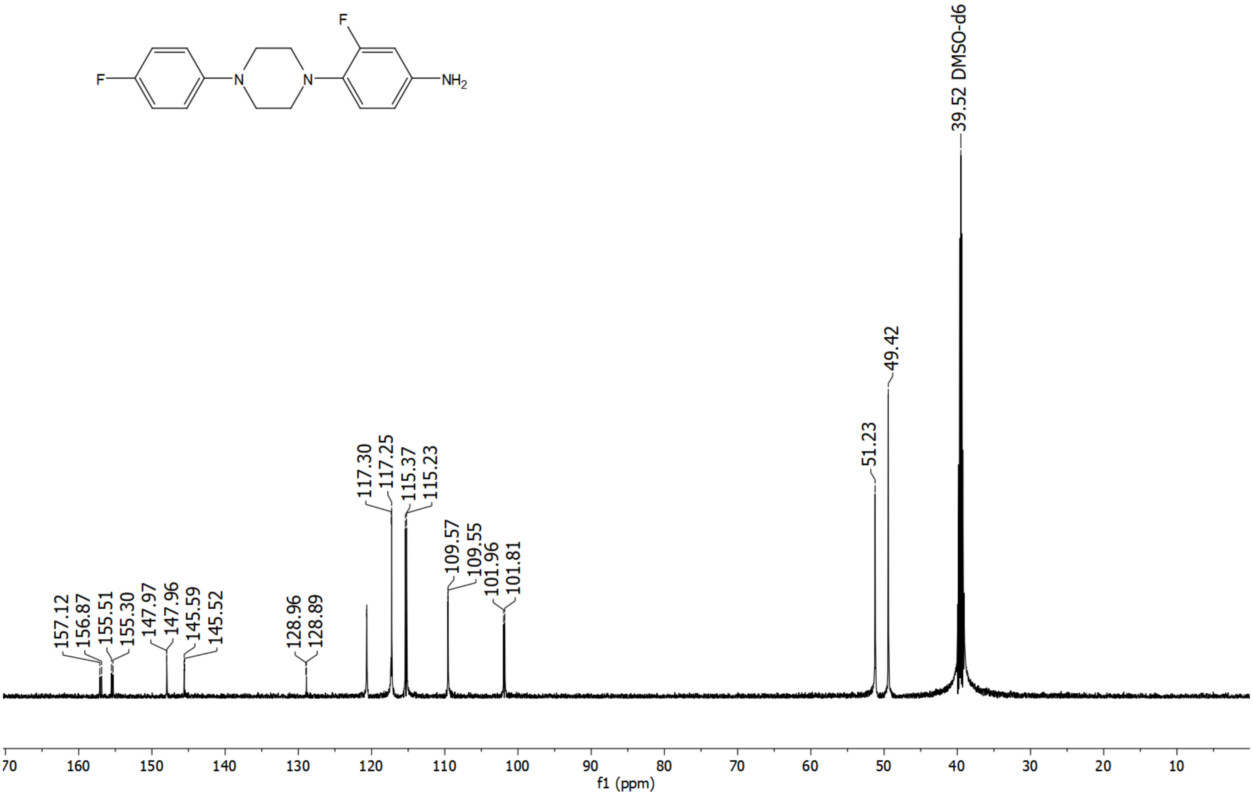

Figure S12: ^{13}C-NMR Spectrum of compound **21** (150 MHz DMSO-d_6).

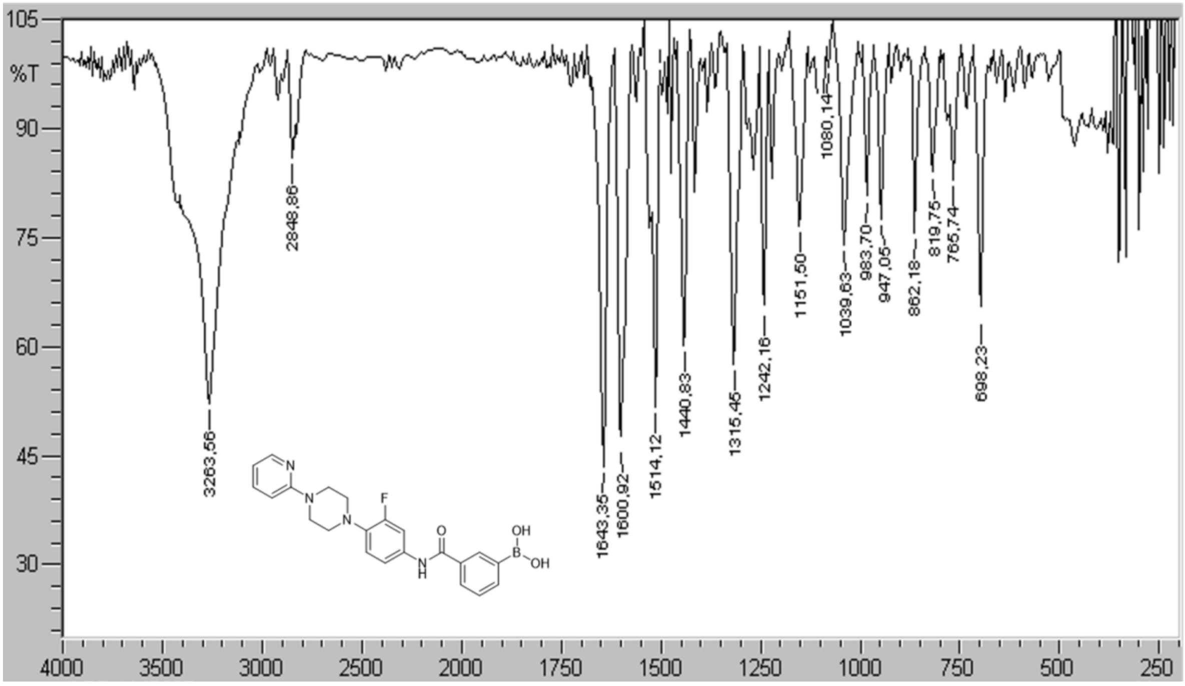

Figure S13: IR Spectrum of compound **29**.

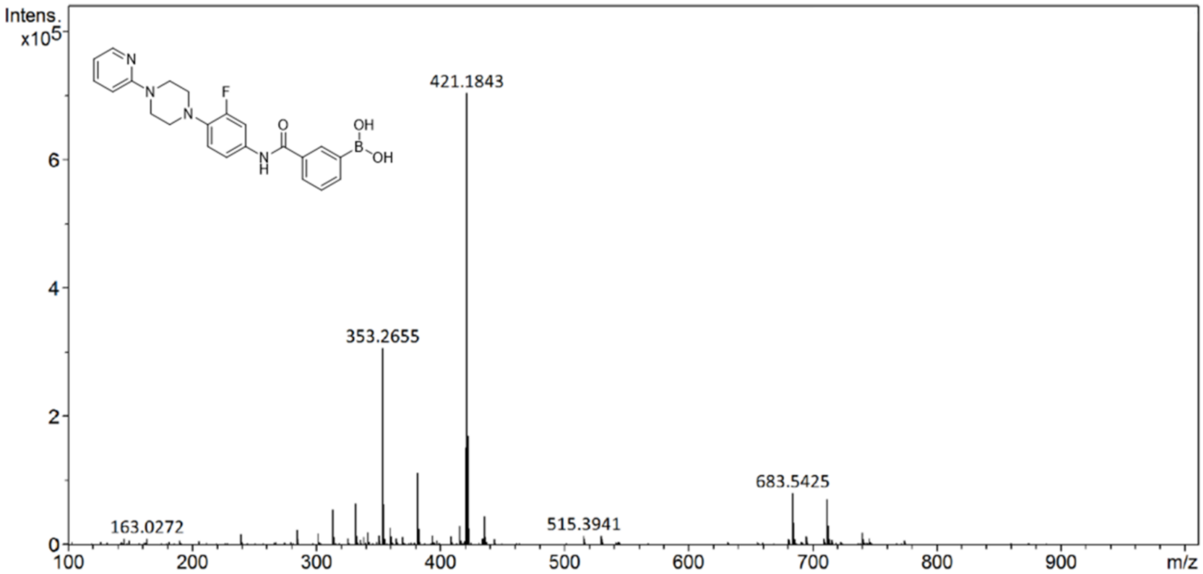

Figure S14: HRMS Spectrum of compound **29** (ESI^{+}).

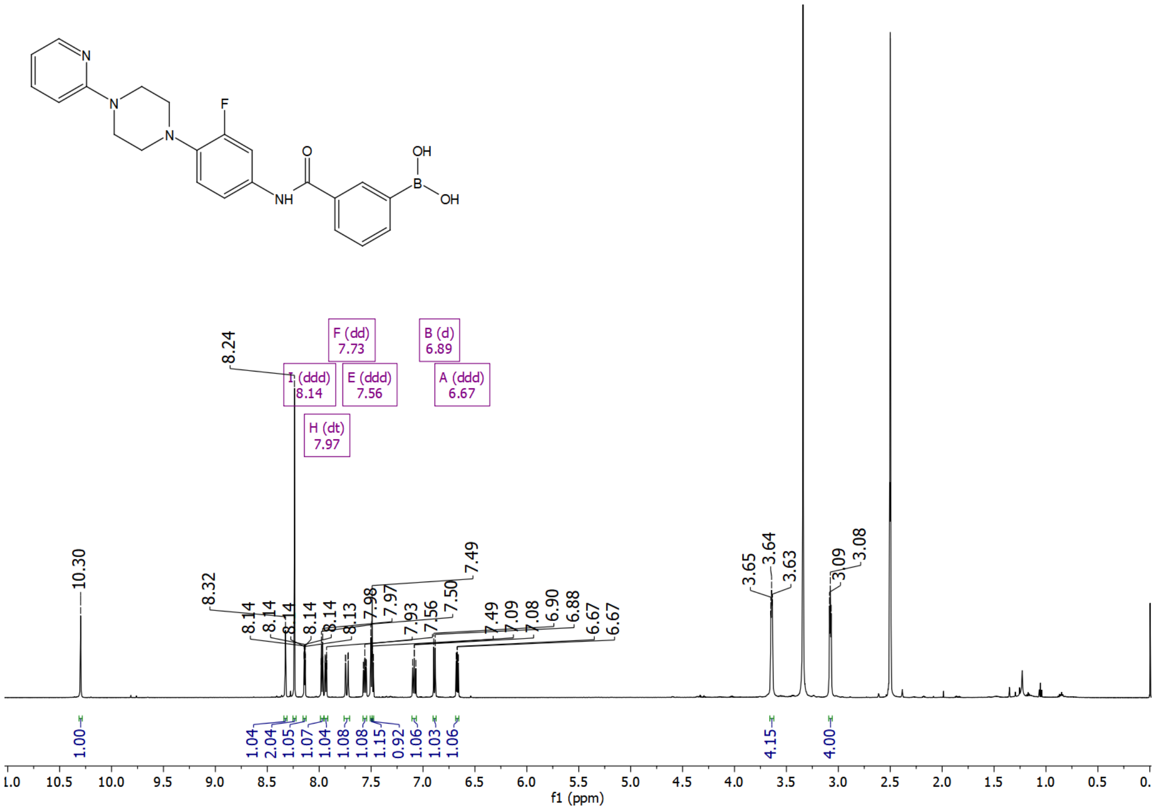

Figure S15: ^{1}H-NMR Spectrum of compound **29** (600 MHz DMSO-d_6).

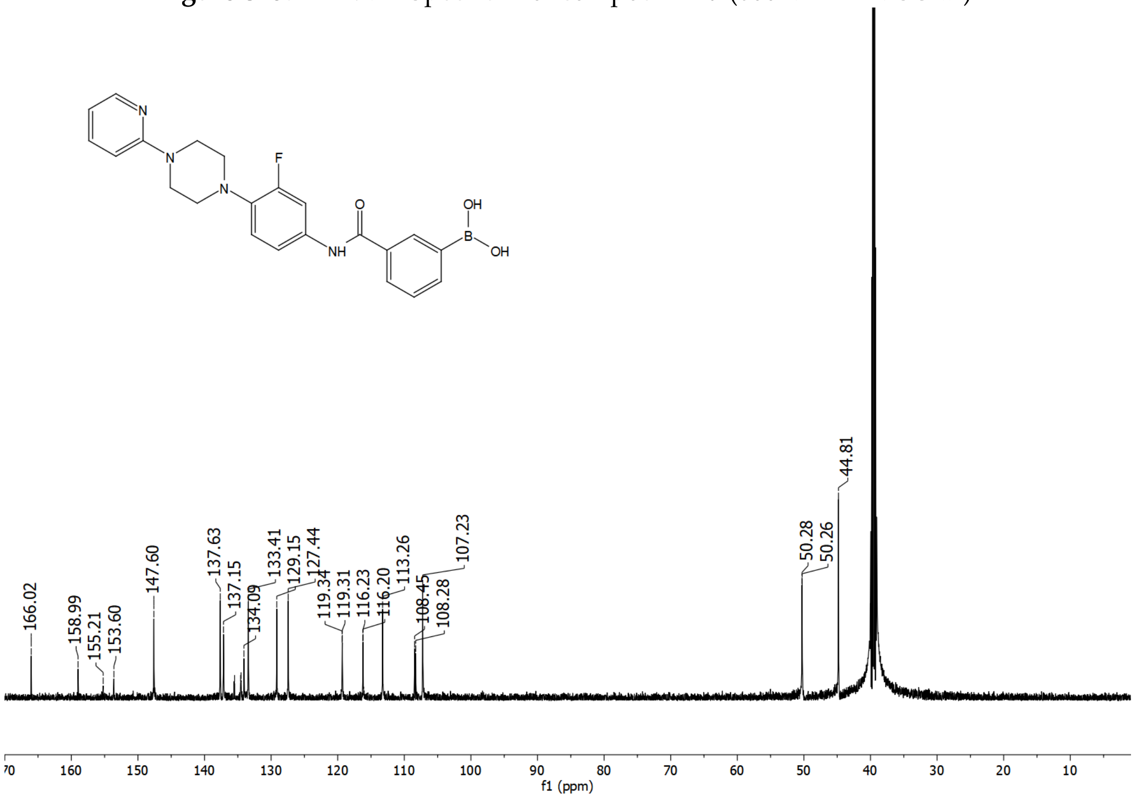

Figure S16: ^{13}C-NMR Spectrum of compound **29** (150 MHz DMSO-d_6).

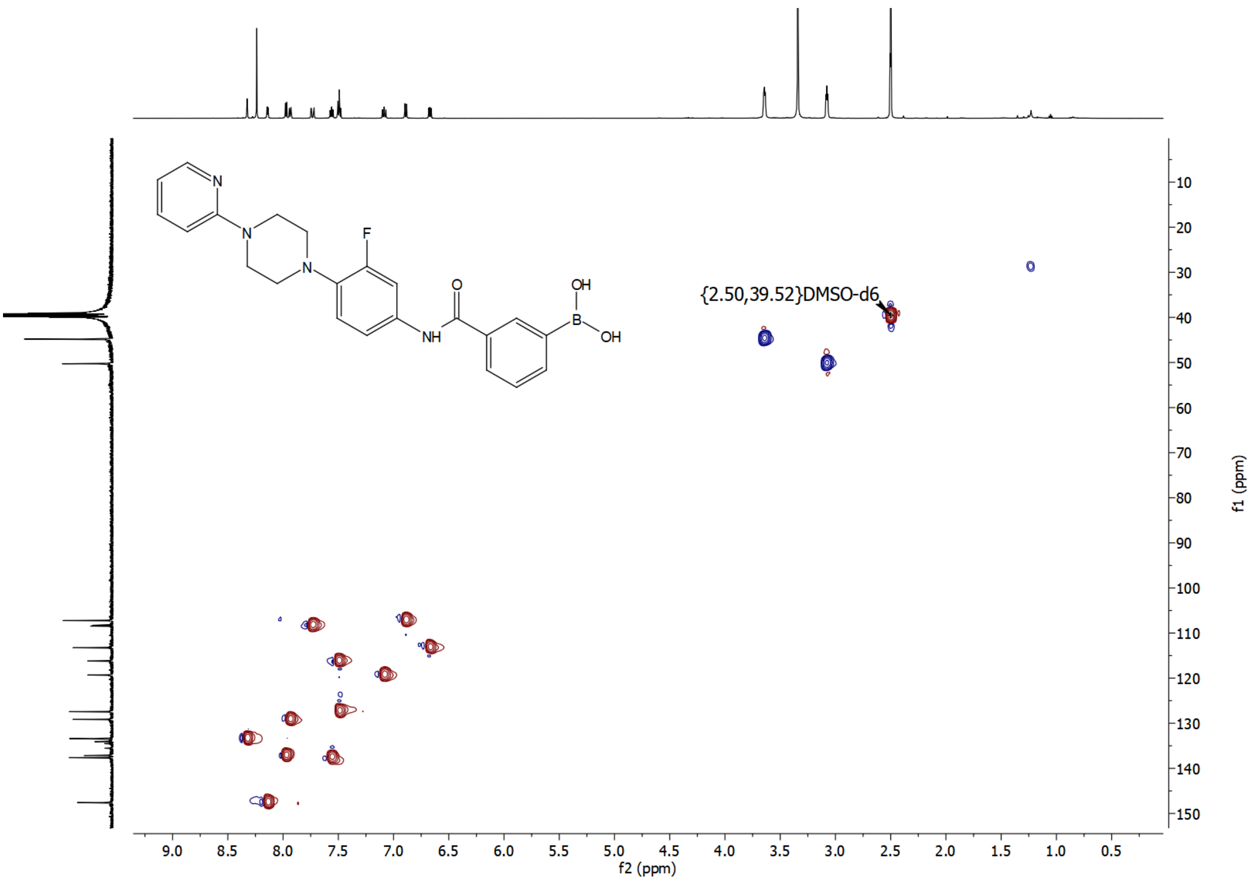

Figure S17: HMQC-NMR Spectrum of compound **29** (600 MHz DMSO-*d*6).

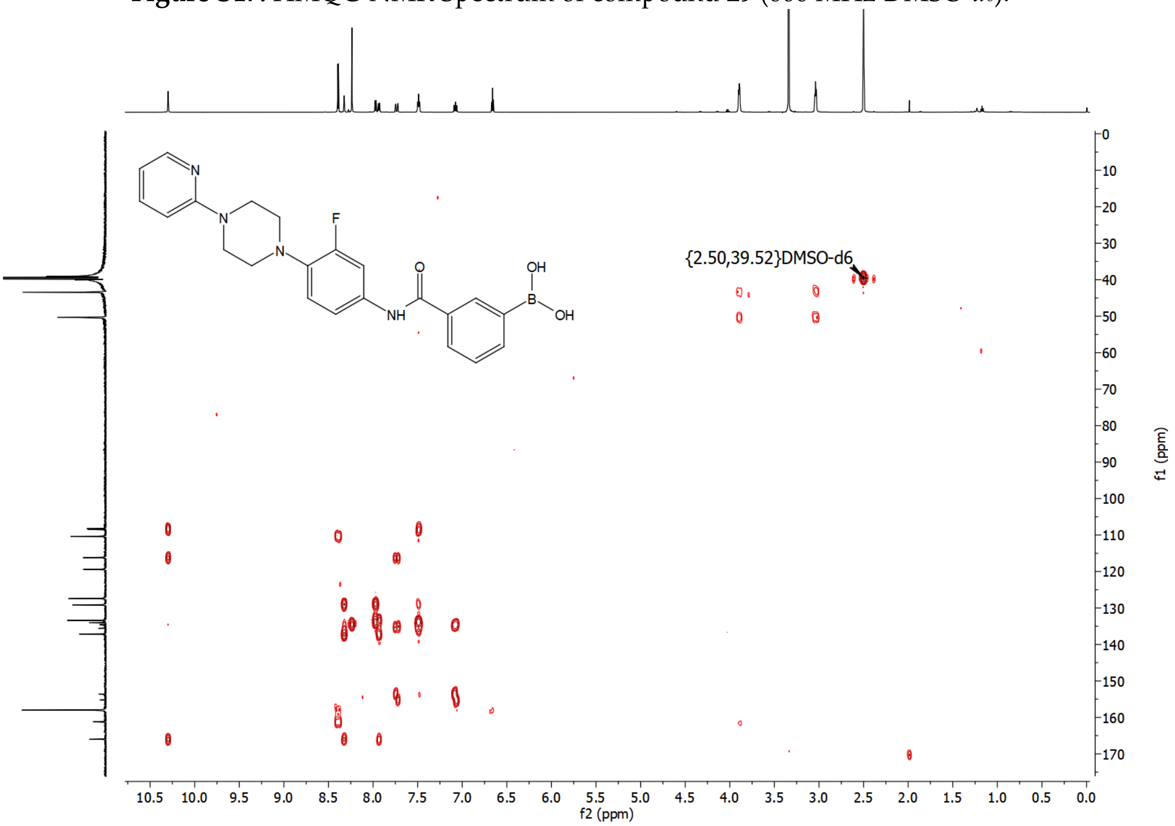

Figure S18: HMBC-NMR Spectrum of compound **29** (600 MHz DMSO-*d*6).

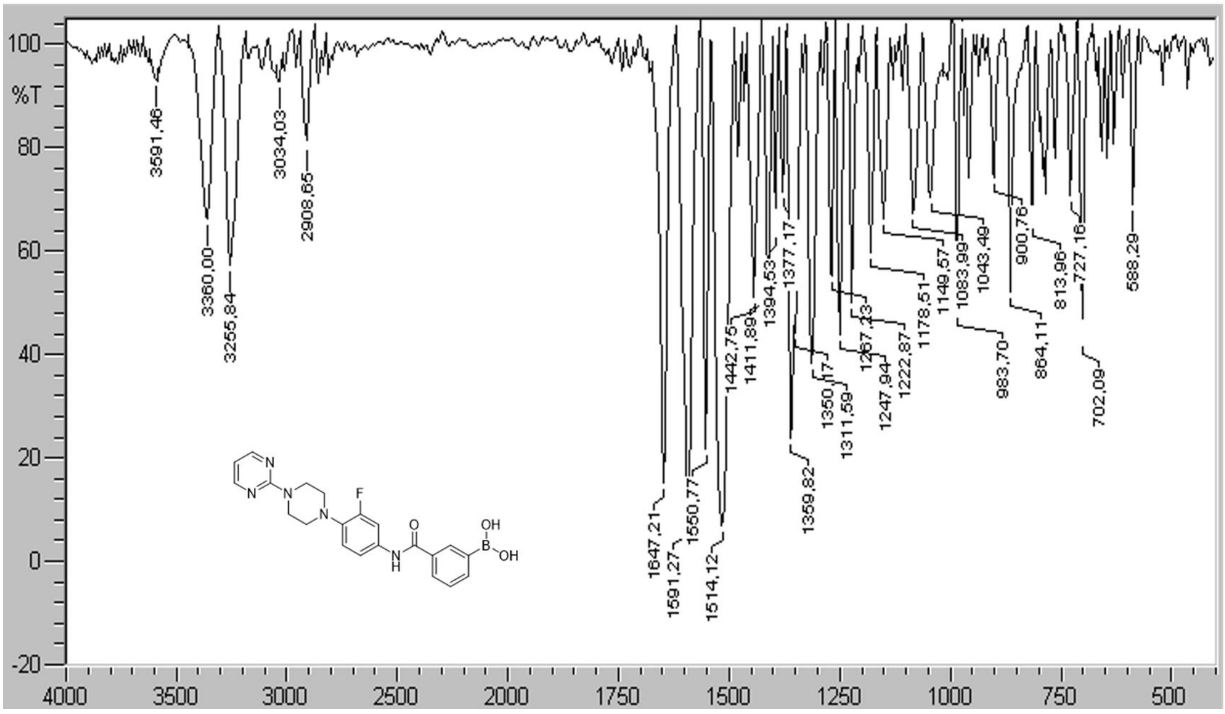

Figure S19: IR Spectrum of compound **30**.

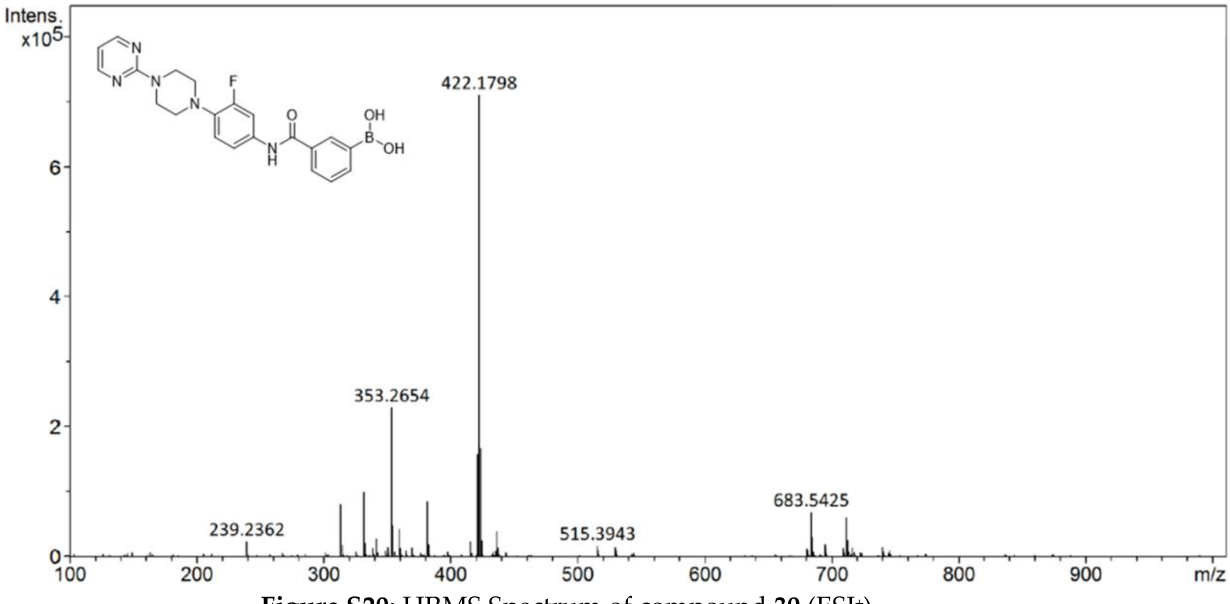

Figure S20: HRMS Spectrum of compound **30** (ESI+).

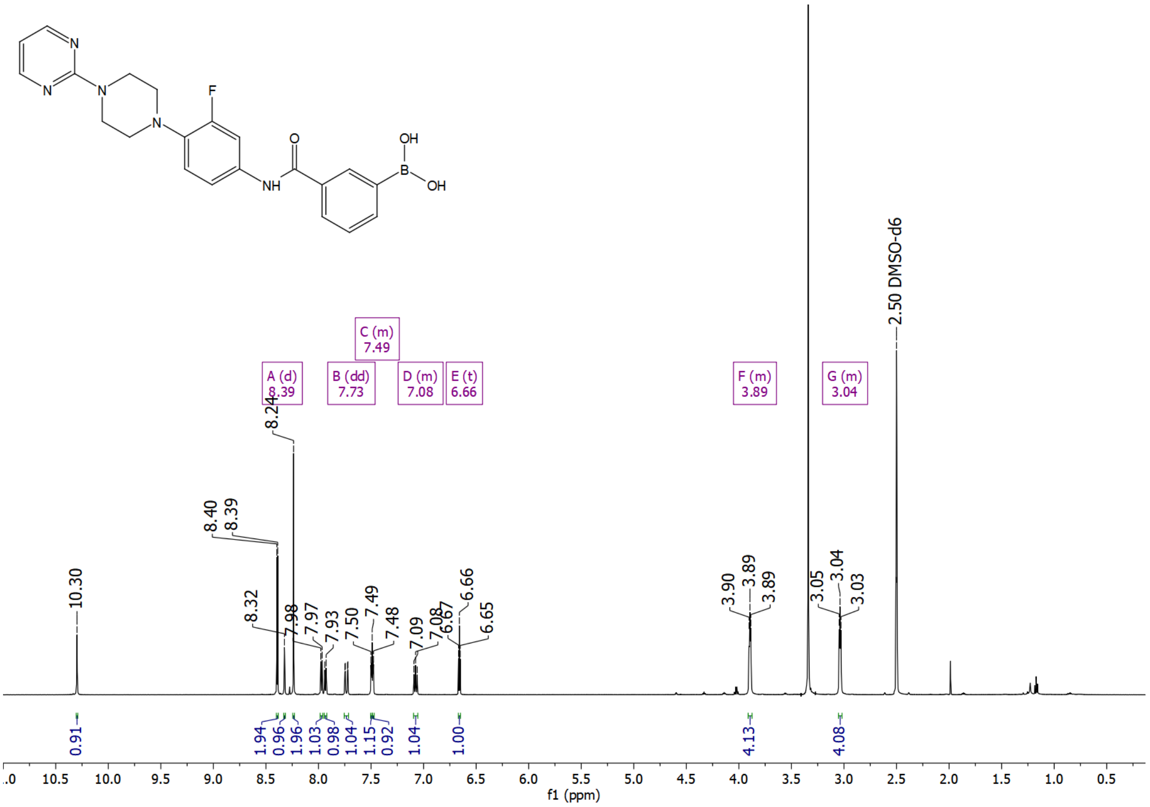

Figure S21: ^{1}H-NMR Spectrum of compound **30** (600 MHz DMSO-d_6).

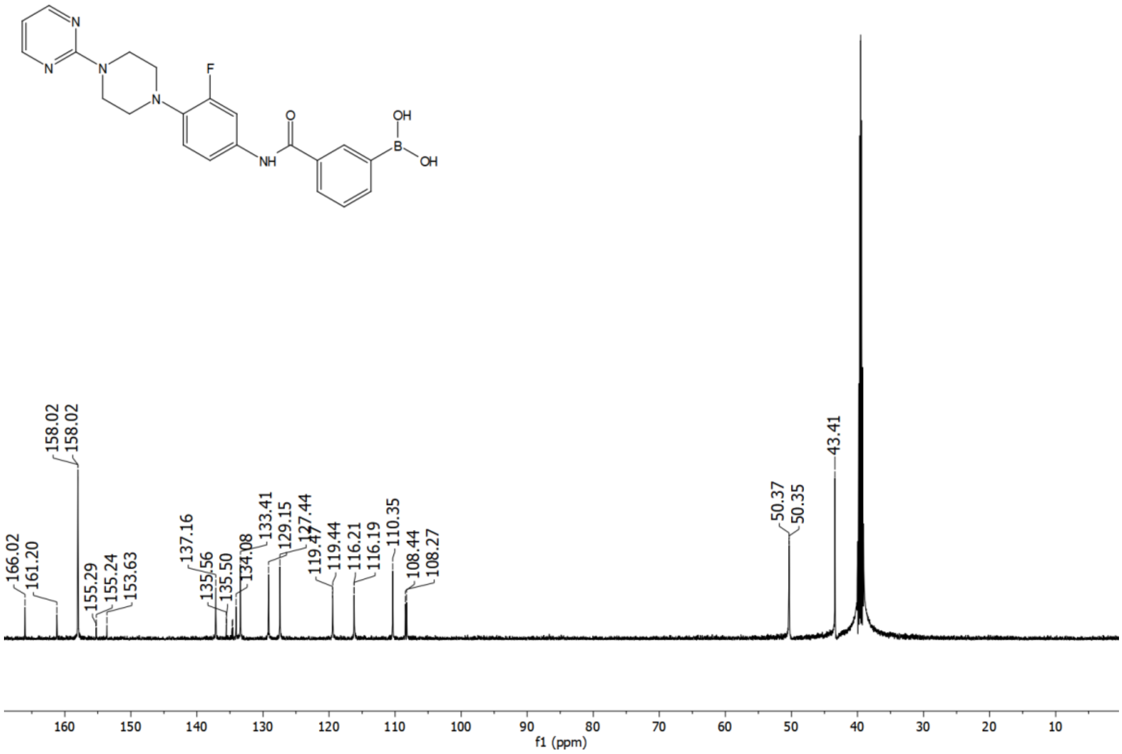

Figure S22: ^{13}C-NMR Spectrum of compound **30** (150 MHz DMSO-d_6).

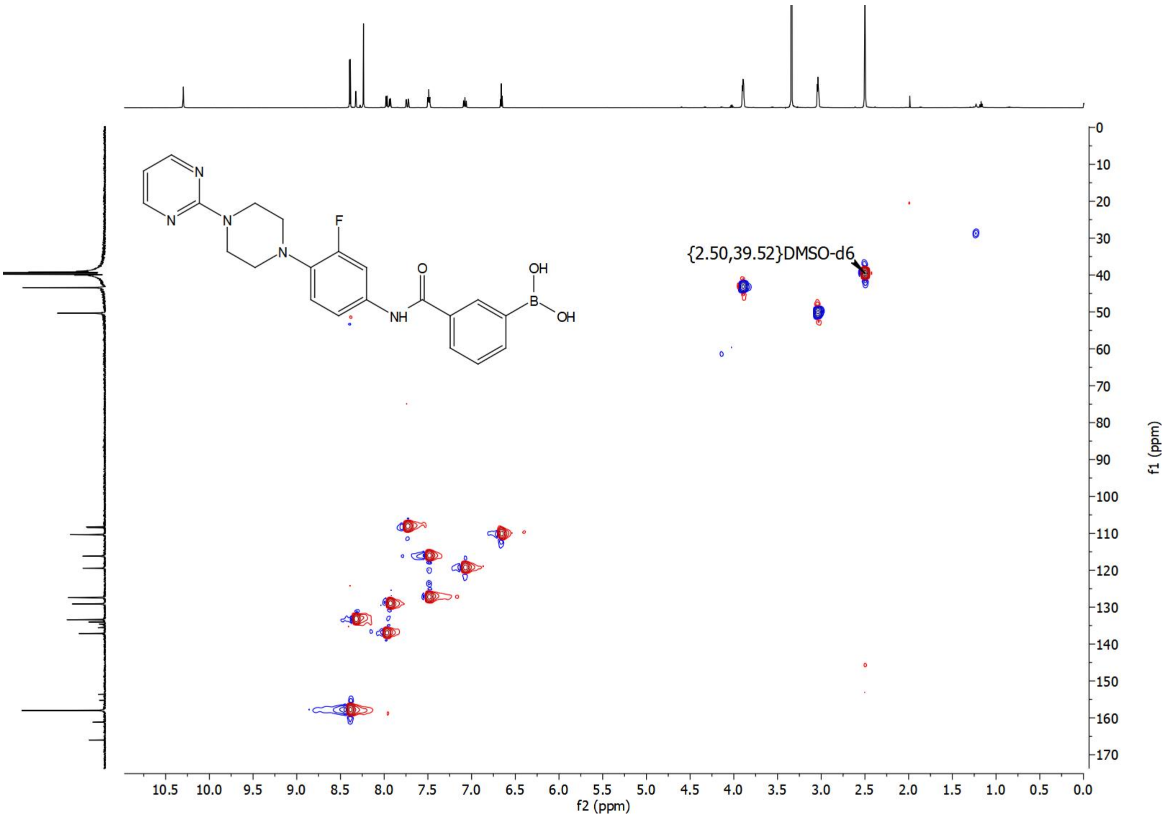

Figure S23: HSQC-NMR Spectrum of compound **30** (600 MHz DMSO-d_6).

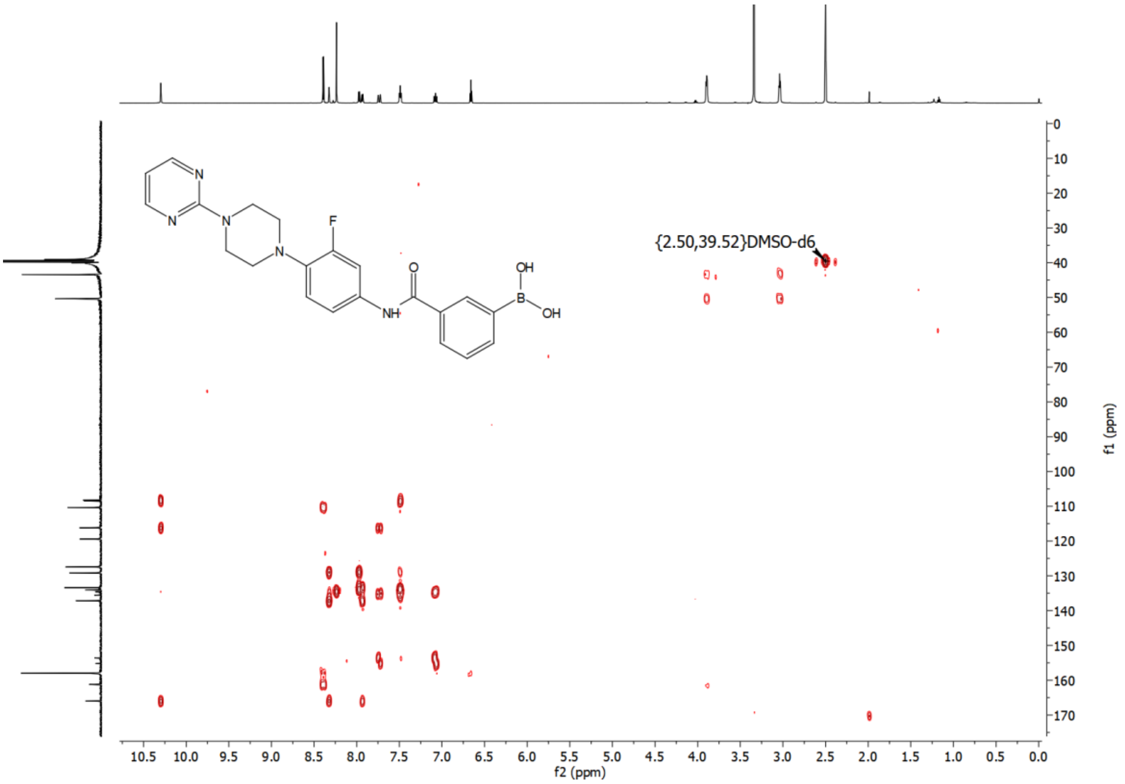

Figure S24: HMBC-NMR Spectrum of compound **30** (600 MHz DMSO-d_6).

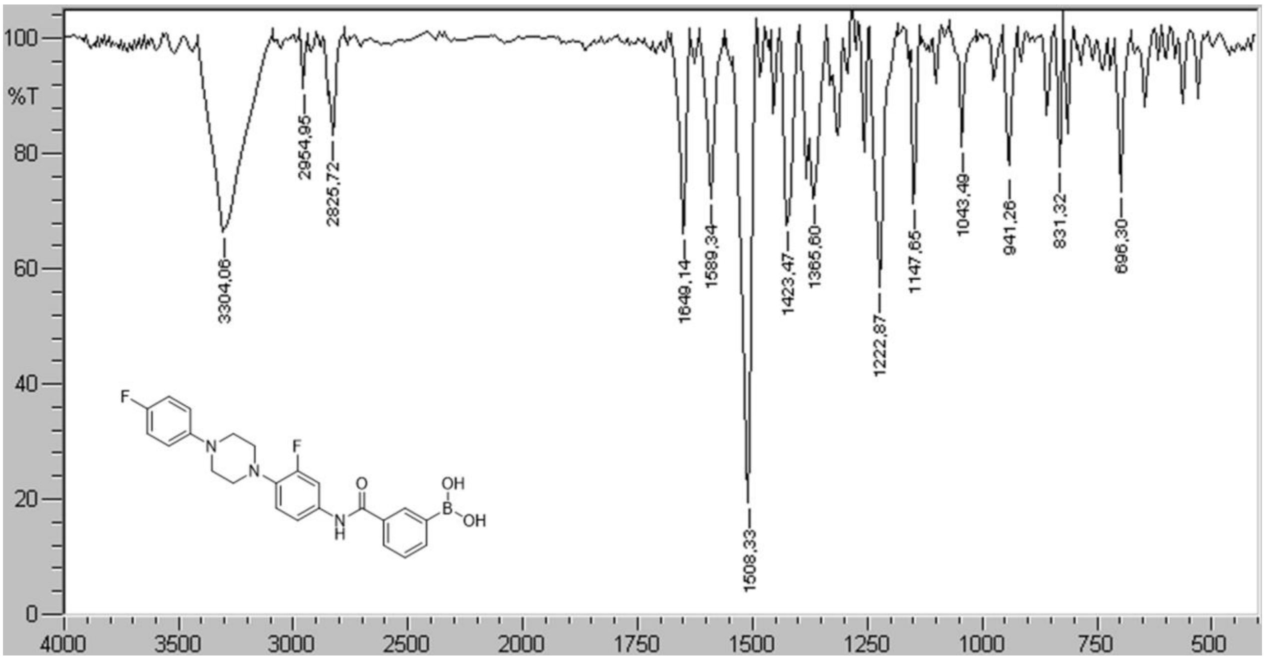

Figure S25: IR Spectrum of compound **31**.

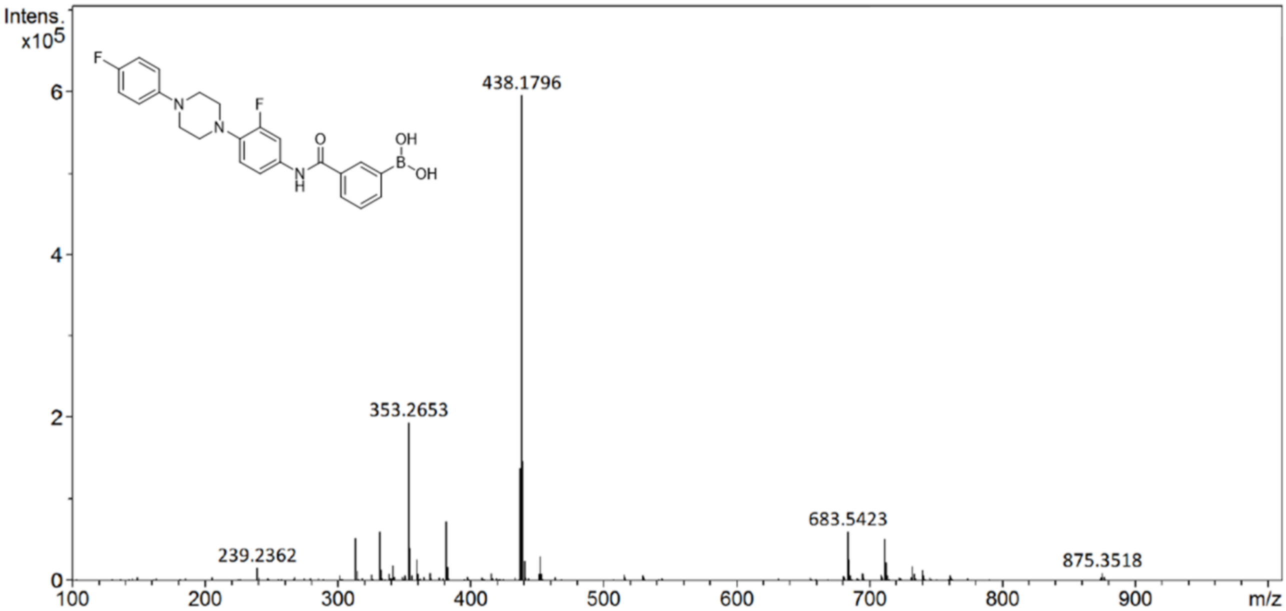

Figure S26: HRMS Spectrum of compound **31** (ESI+).

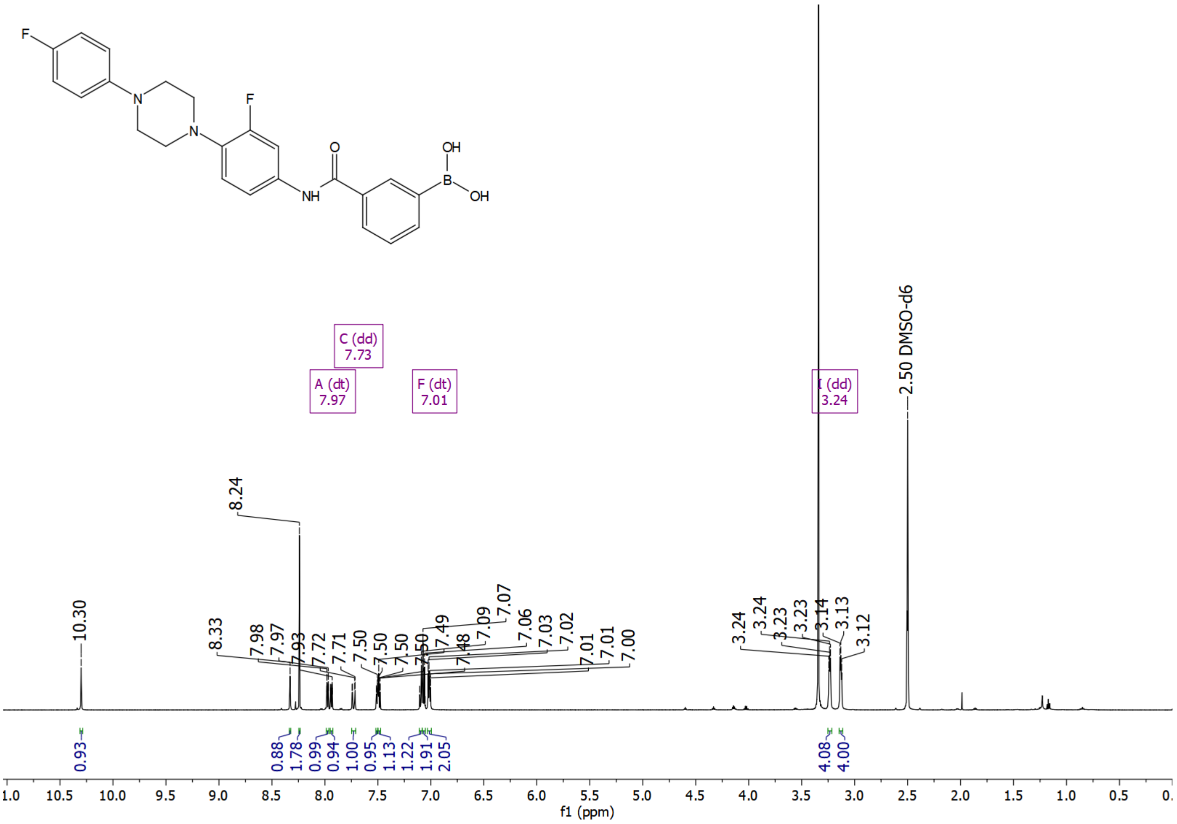

Figure S27: ^{1}H-NMR Spectrum of compound **31** (600 MHz DMSO-d_6).

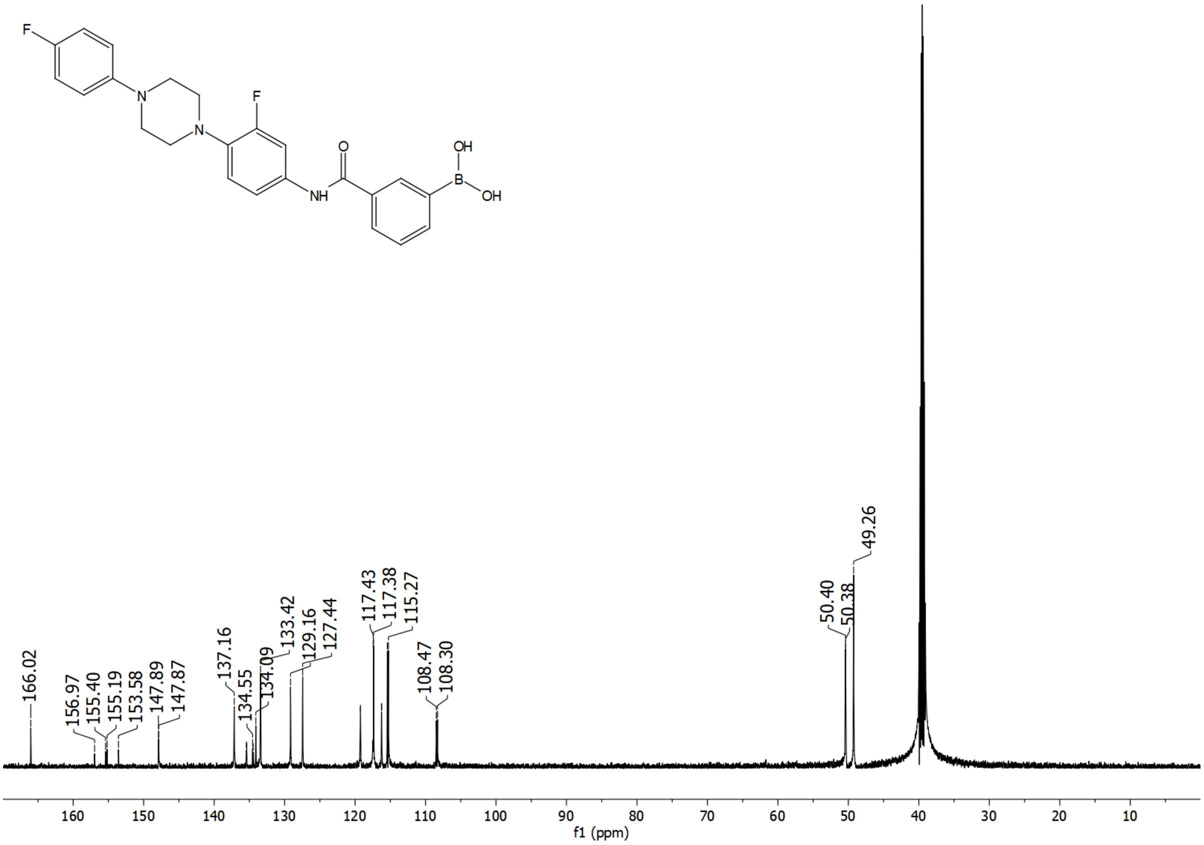

Figure S28: ^{13}C-NMR Spectrum of compound **31** (150 MHz DMSO-d_6).

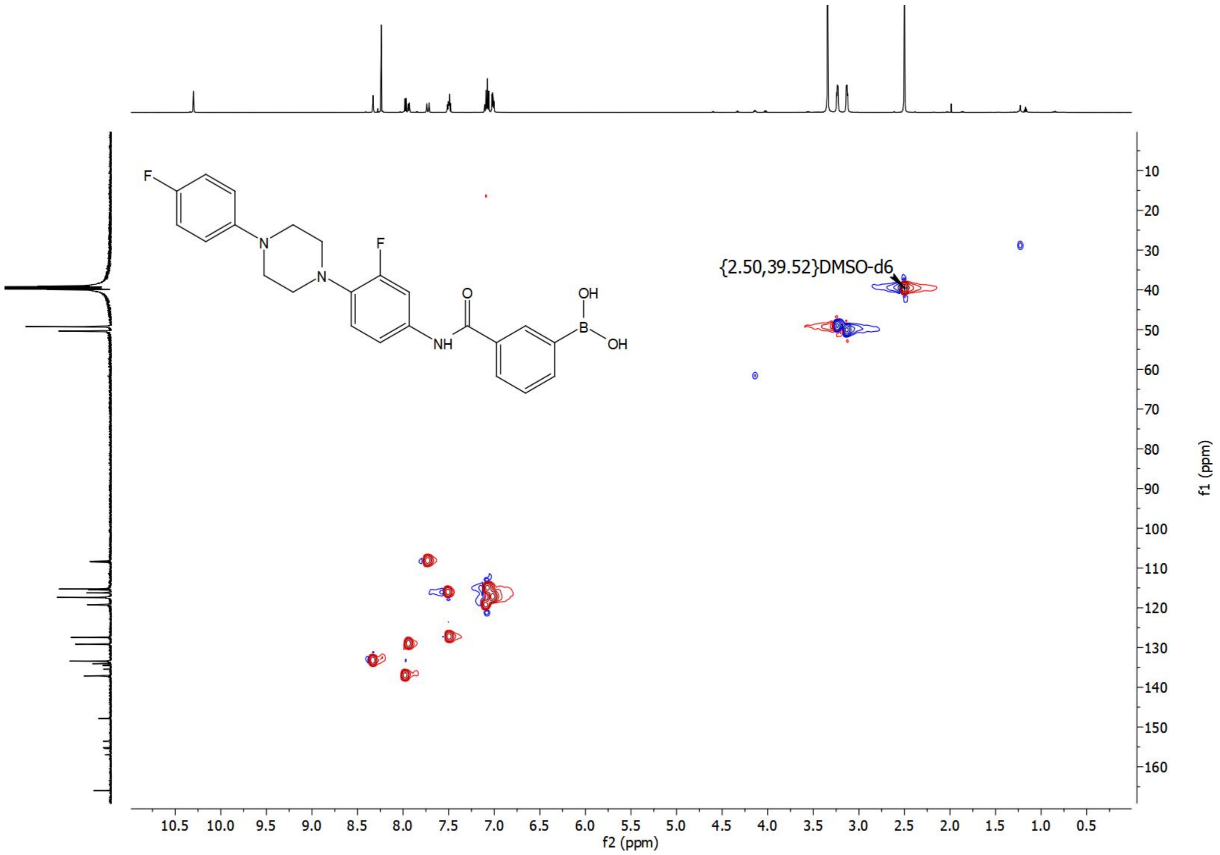

Figure S29: HSQC-NMR Spectrum of compound **31** (600 MHz DMSO-d_6).

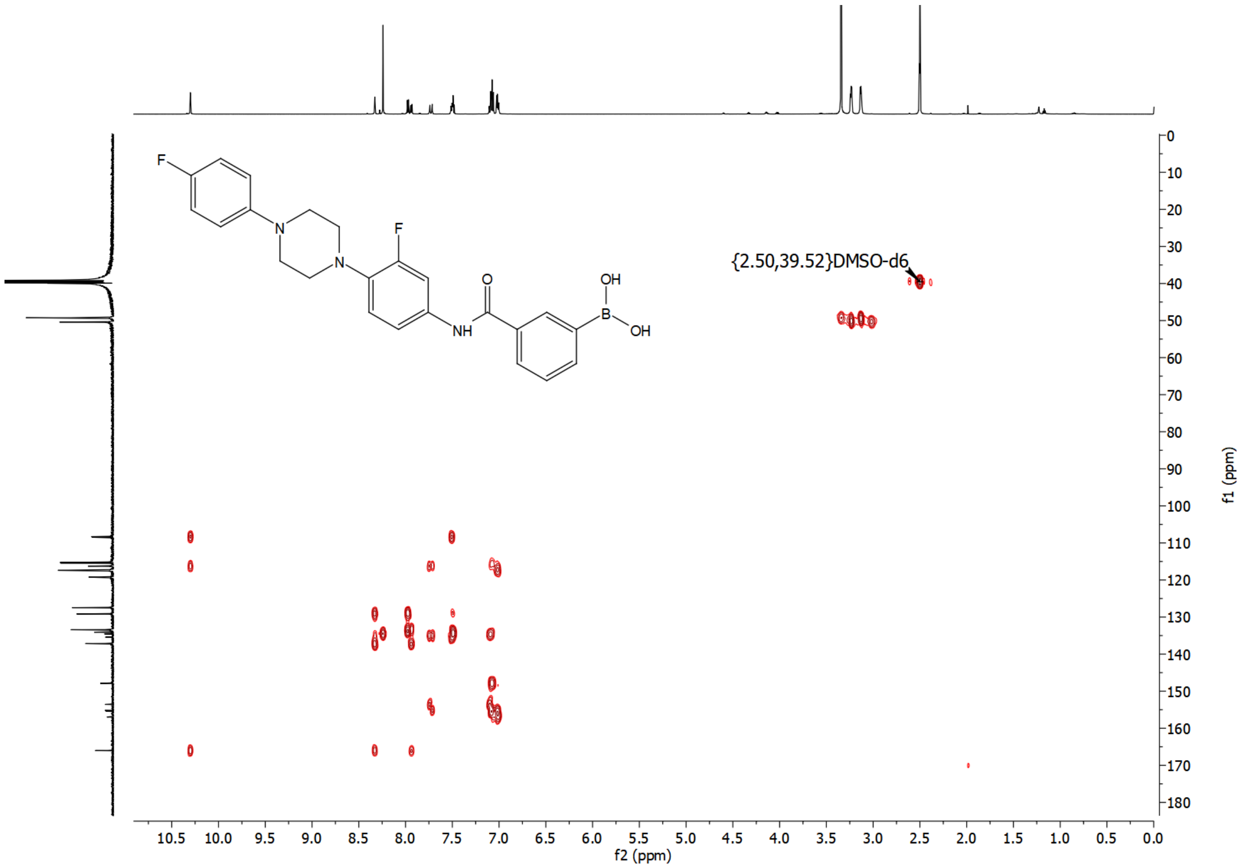

Figure S30: HMBC-NMR Spectrum of compound **31** (600 MHz DMSO-d_6).

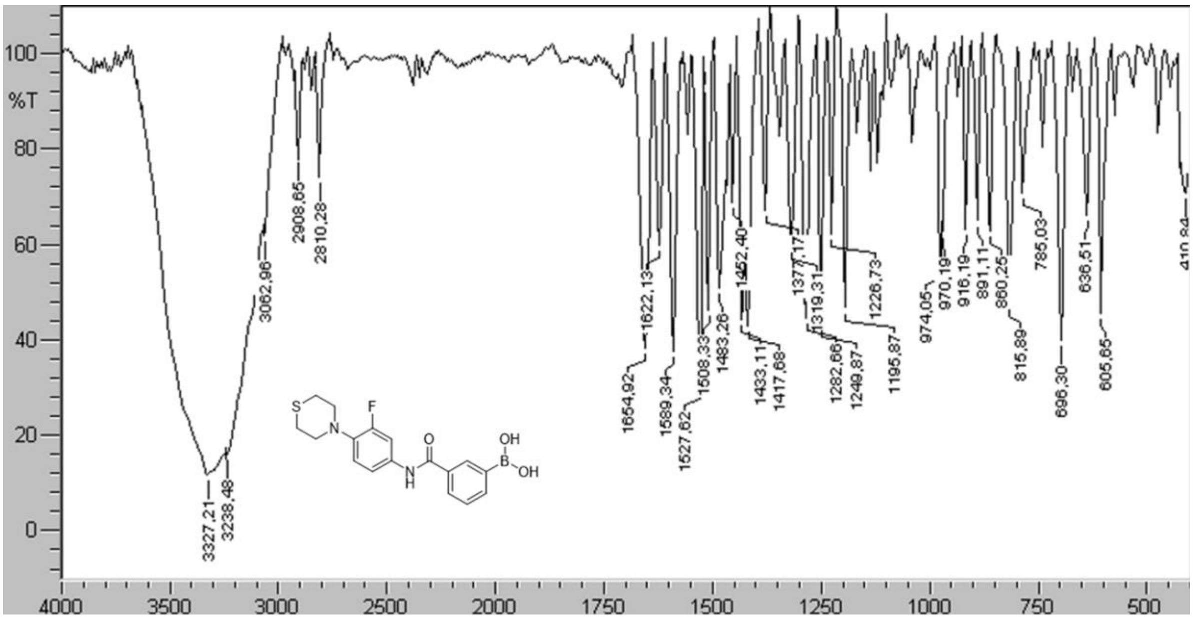

Figure S31: IR Spectrum of compound **32**.

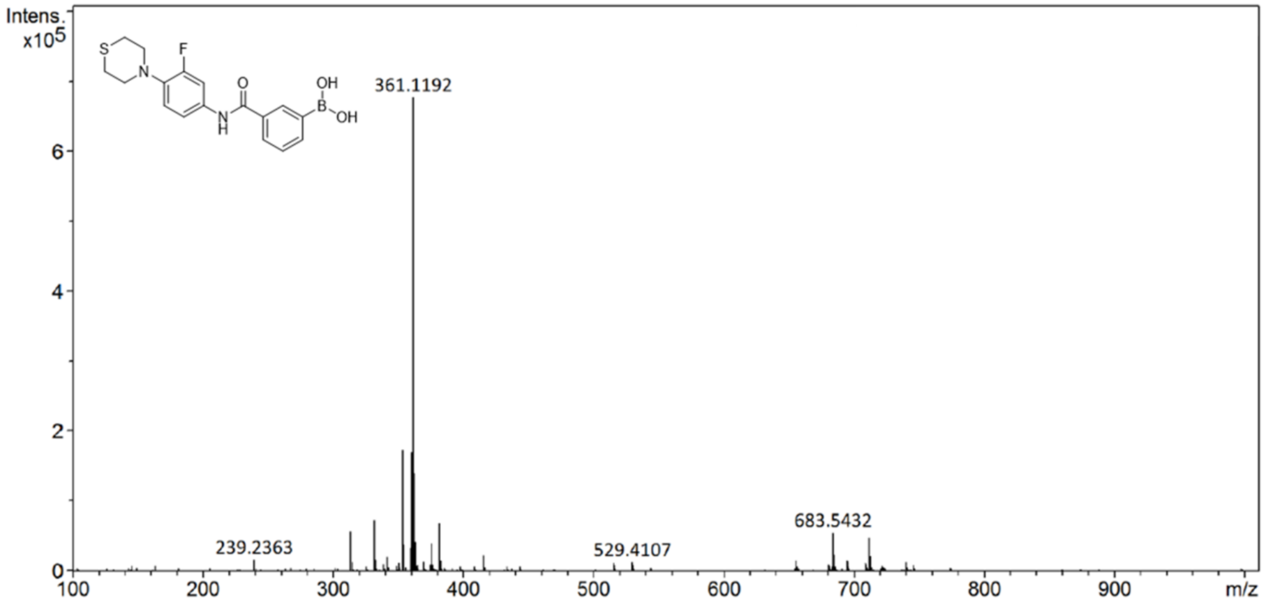

Figure S32: HRMS Spectrum of compound **32** (ESI+).

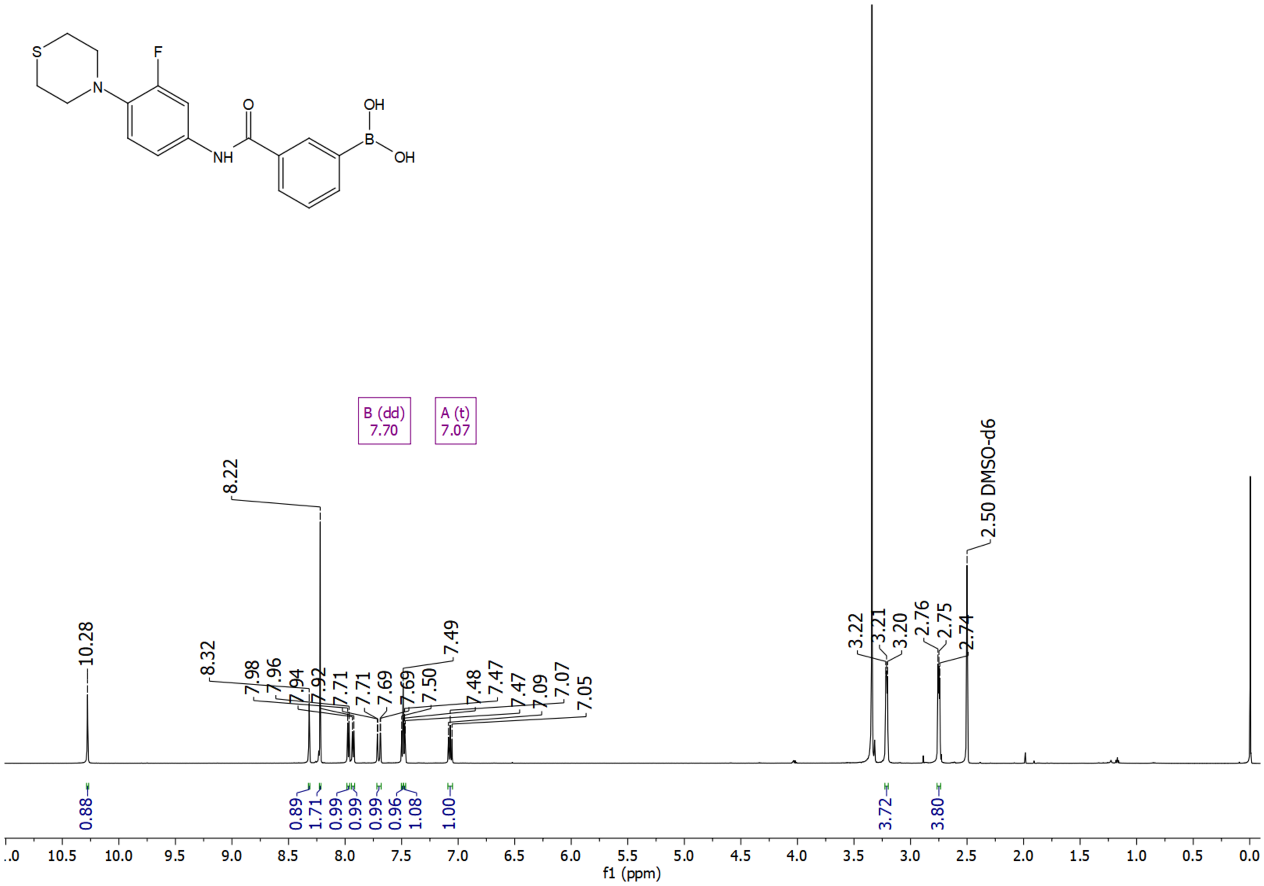

Figure S33: ^{1}H NMR Spectrum of compound **32**.

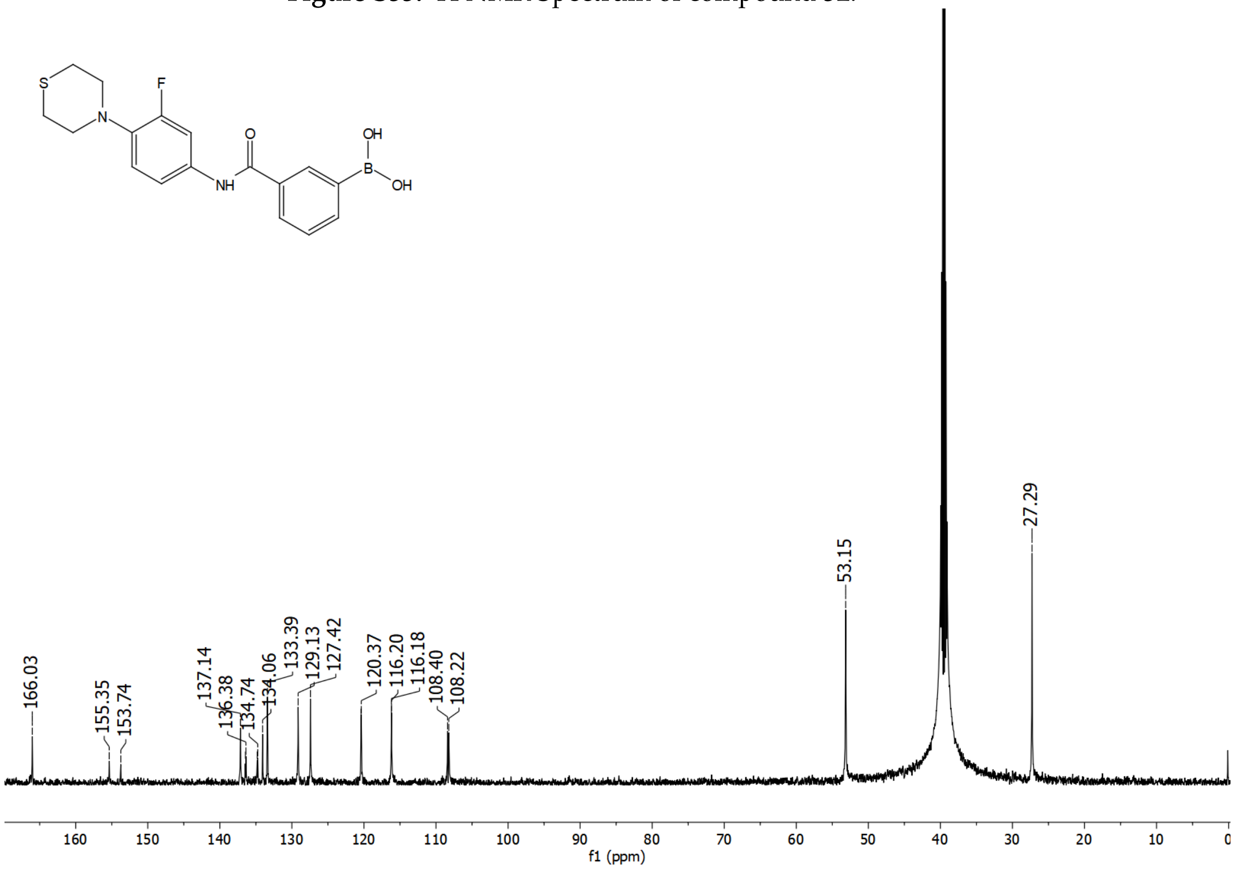

Figure S34: ^{13}C NMR Spectrum of compound **32**.

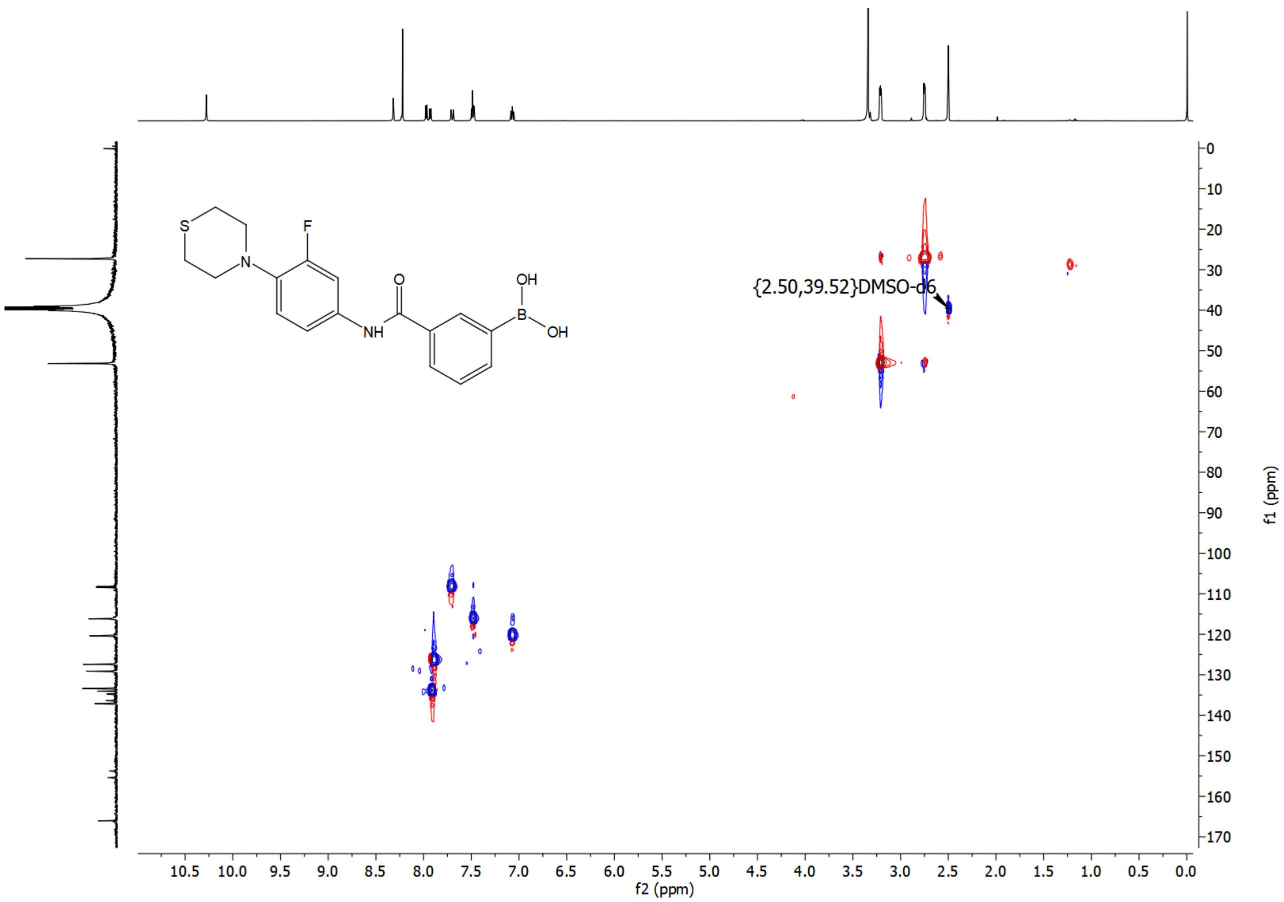

Figure S35: HSC NMR Spectrum of compound **32**.

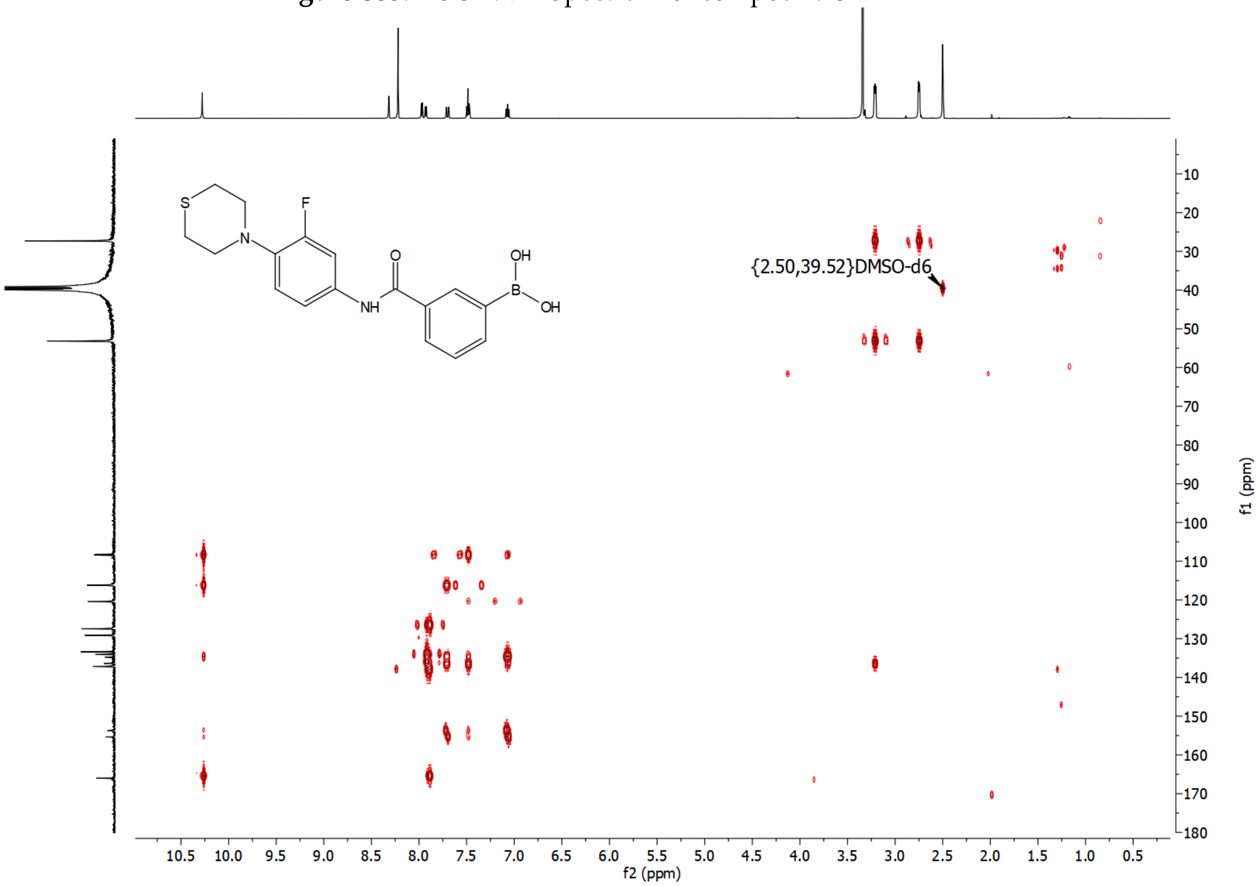

Figure S36: HMBC NMR Spectrum of compound **32**.

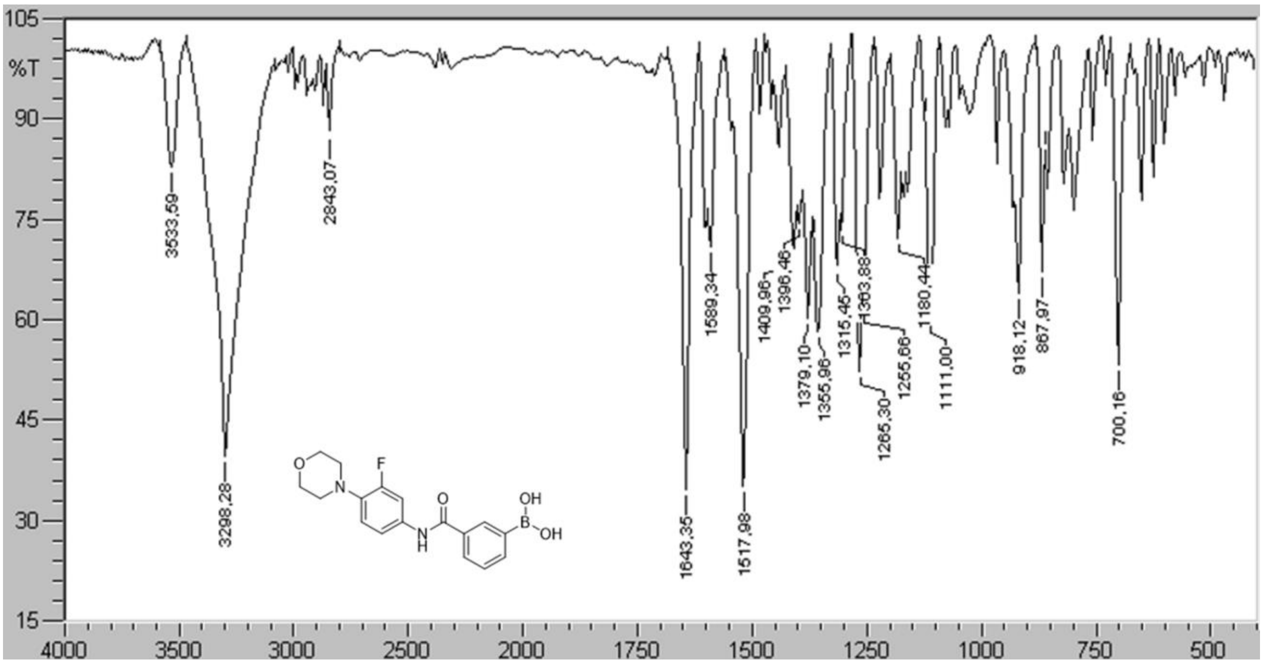

Figure S37: IR Spectrum of compound **33**.

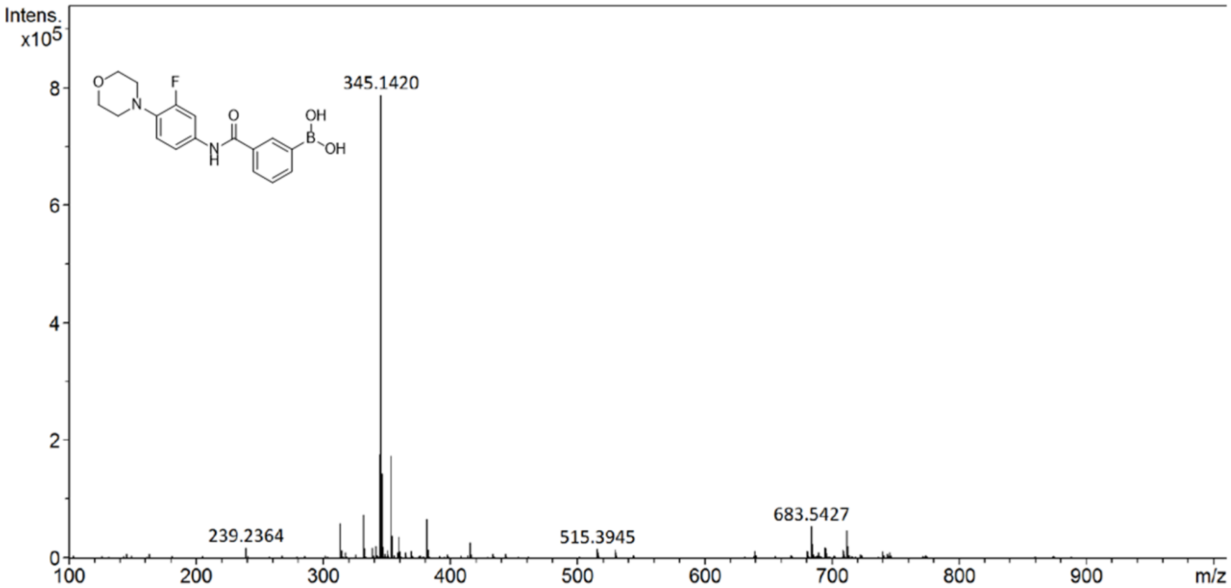

Figure S38: HRMS Spectrum of compound **33** (ESI+).

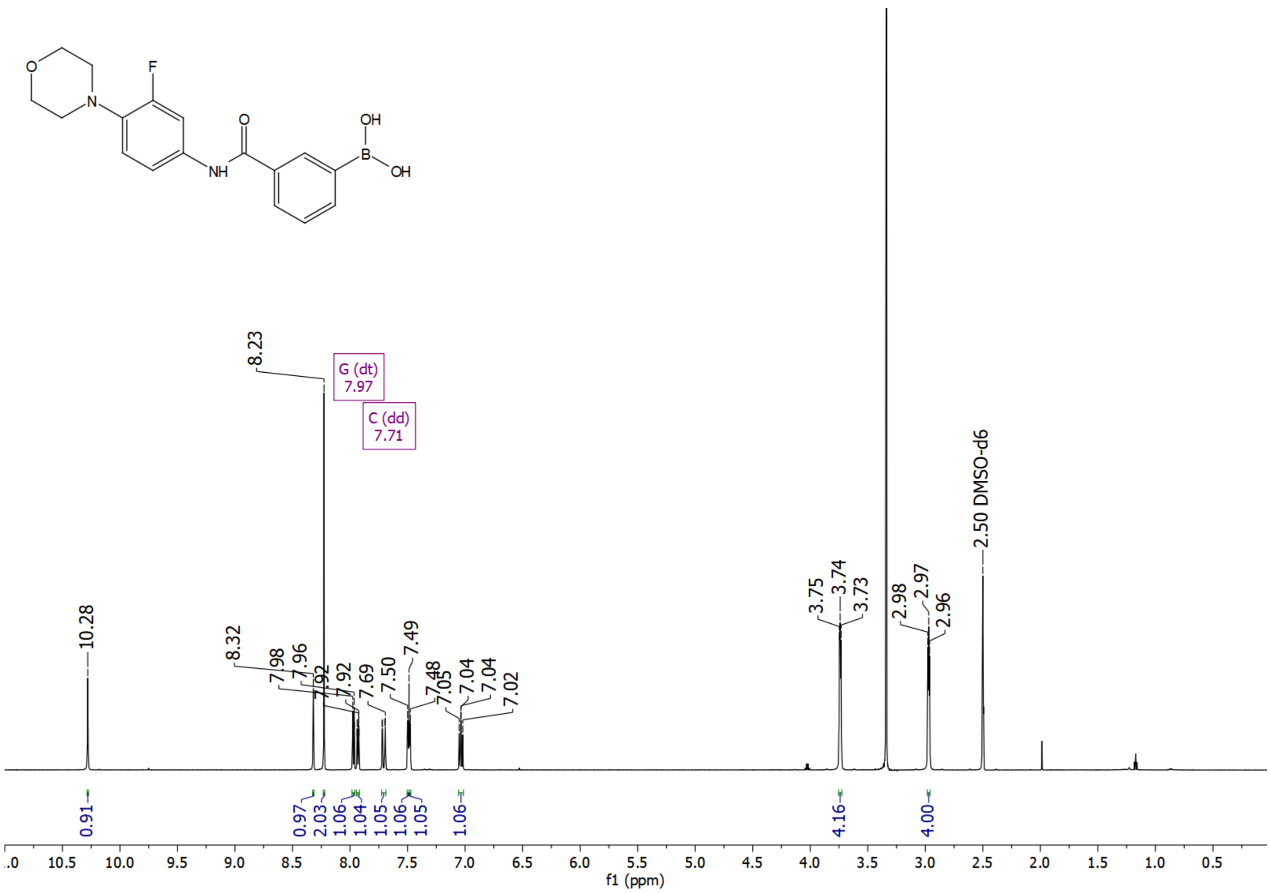

Figure S39: ^{1}H-NMR Spectrum of compound **33** (600 MHz DMSO-d_6).

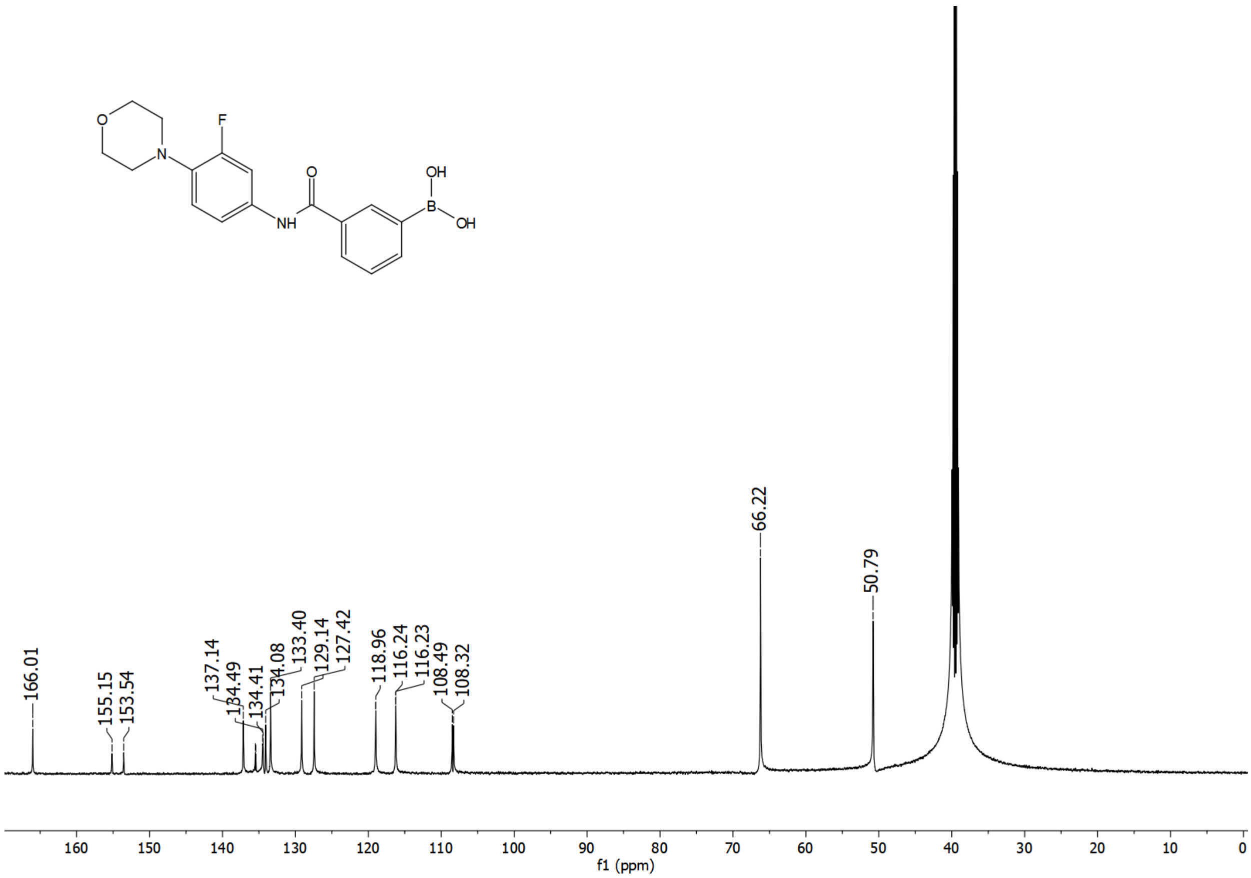

Figure S40: ^{13}C-NMR Spectrum of compound **33** (150 MHz DMSO-d_6).

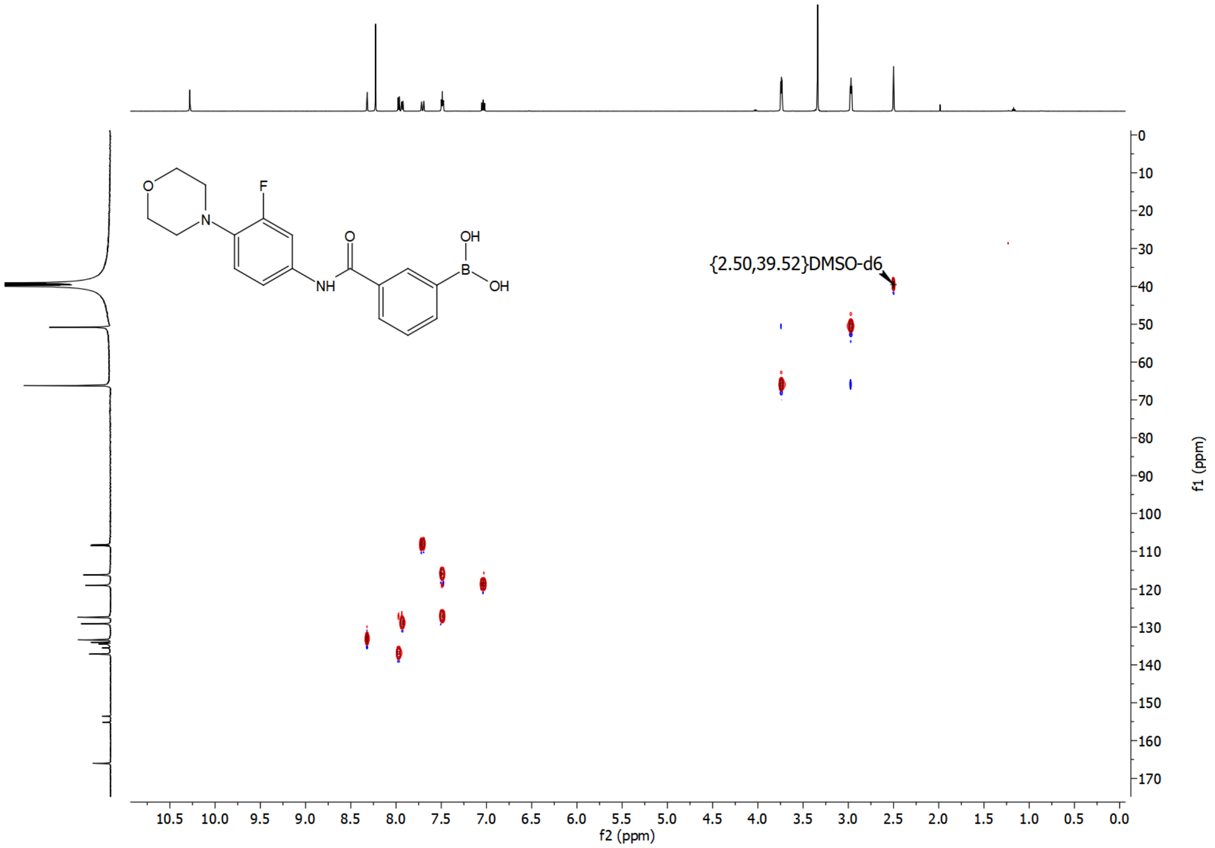

Figure S41: HSQC-NMR Spectrum of compound **33** (600 MHz DMSO-d_6).

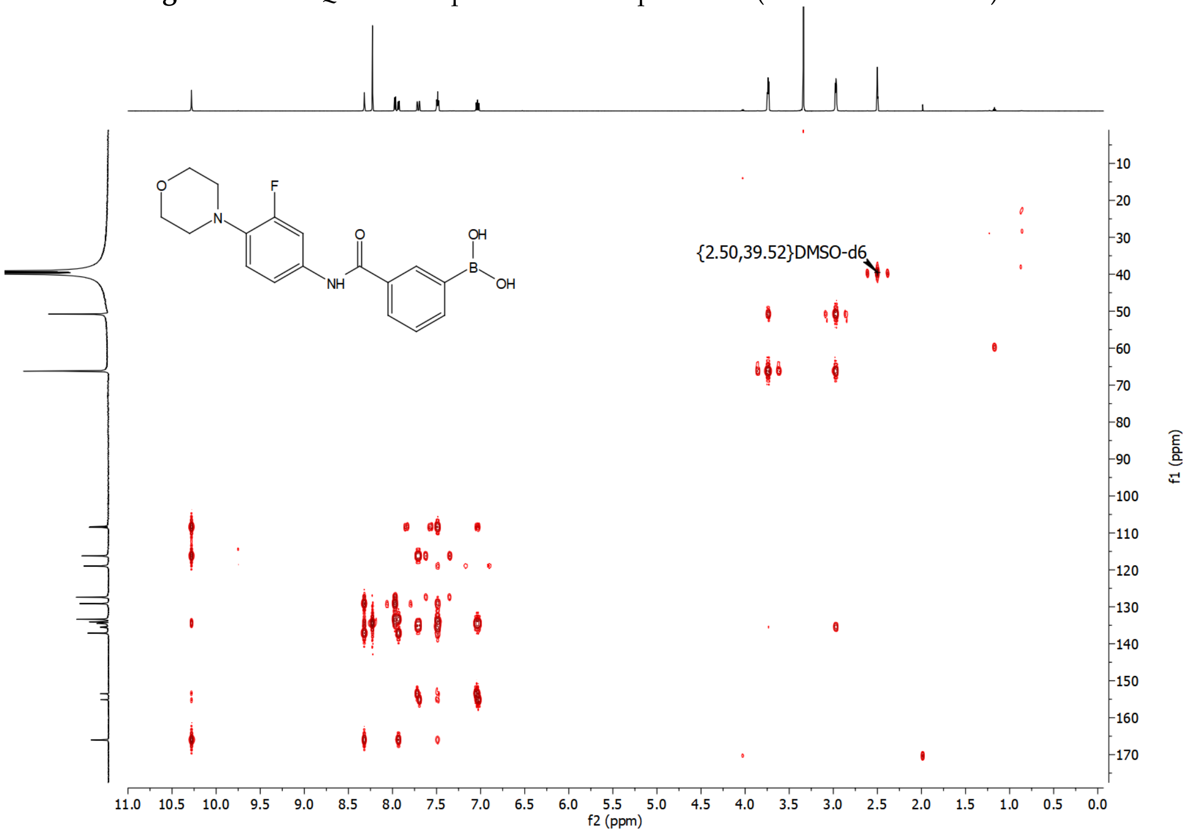

Figure S42: HMBC-NMR Spectrum of compound **33** (600 MHz DMSO-d_6).

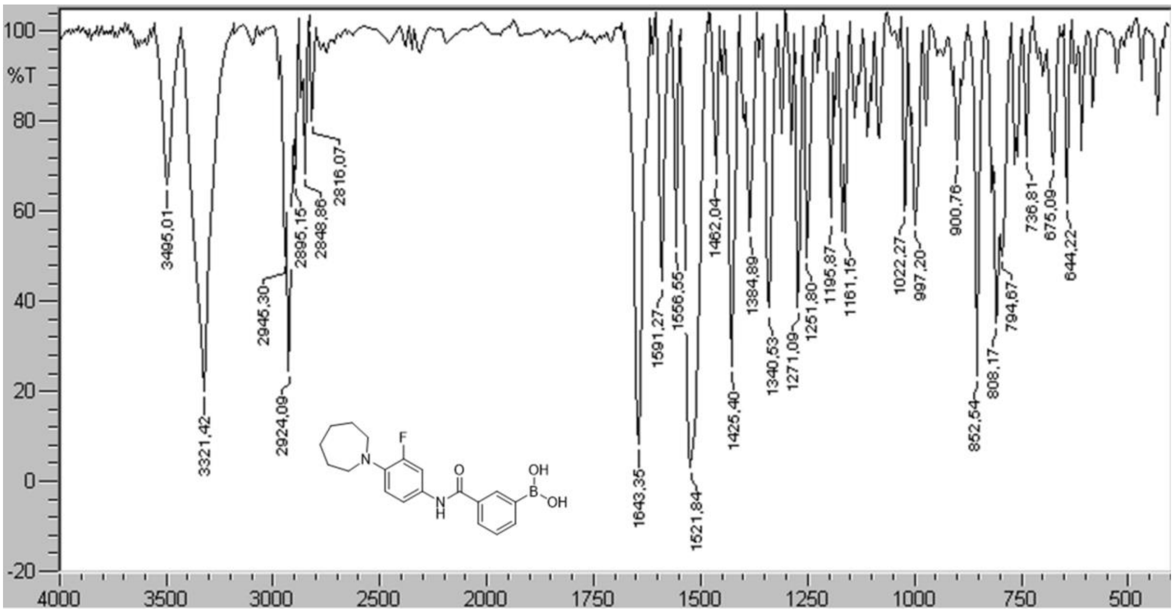

Figure S43: IR Spectrum of compound **34**.

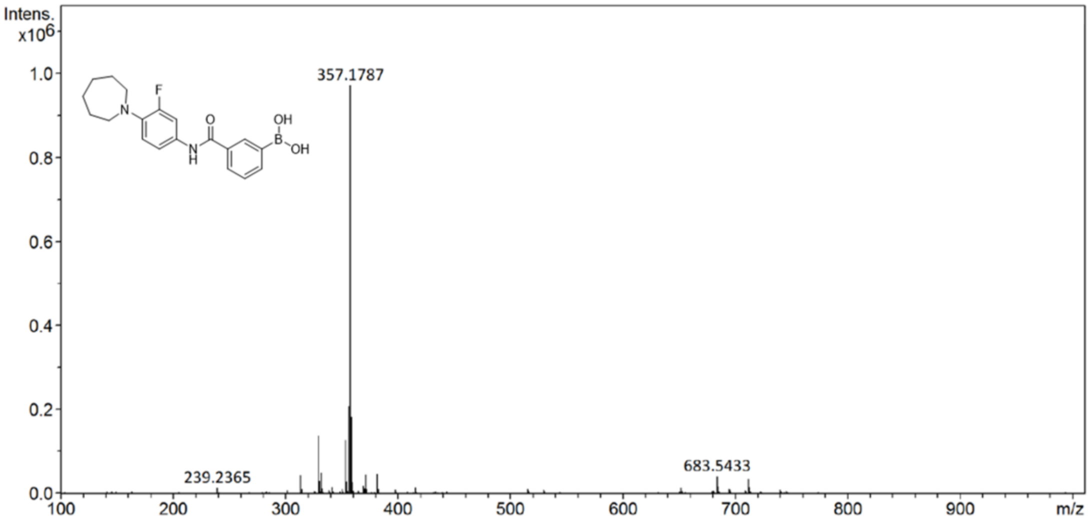

Figure S44: HRMS Spectrum of compound **34** (ESI+).

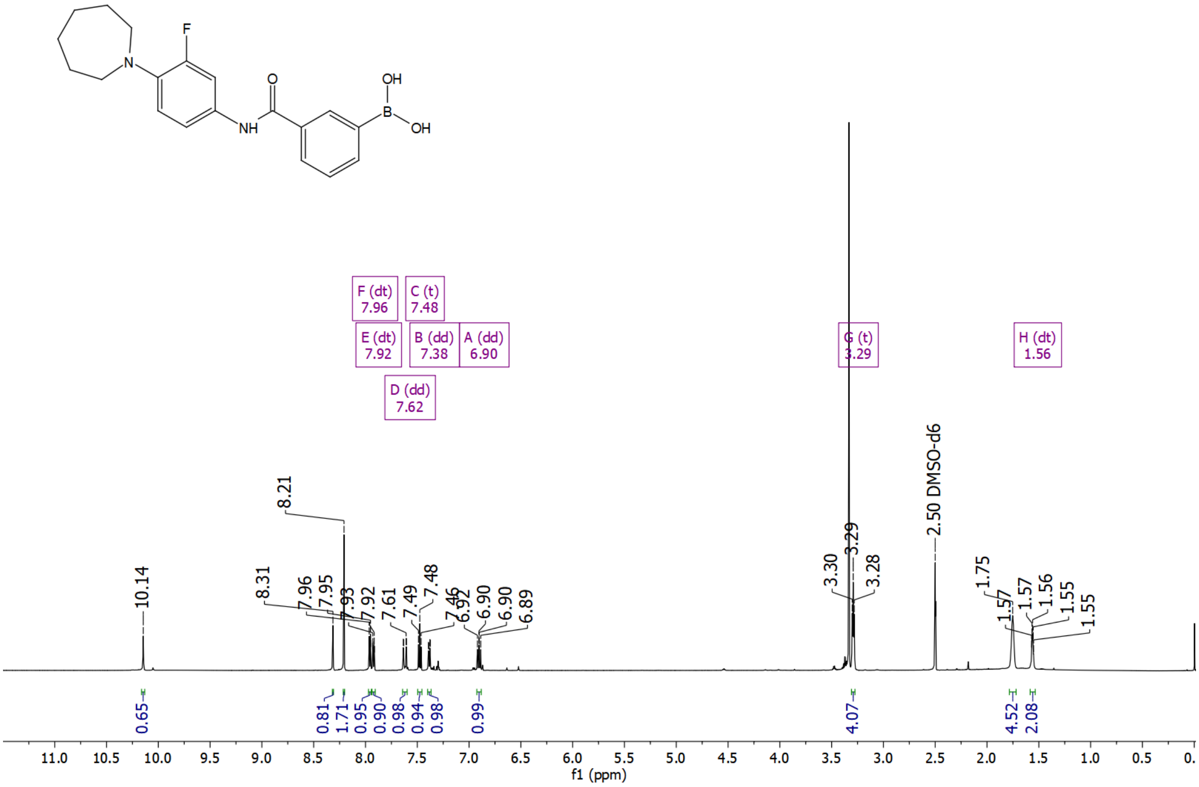

Figure S45: ^{1}H-NMR Spectrum of compound **34** (600 MHz DMSO-d_6).

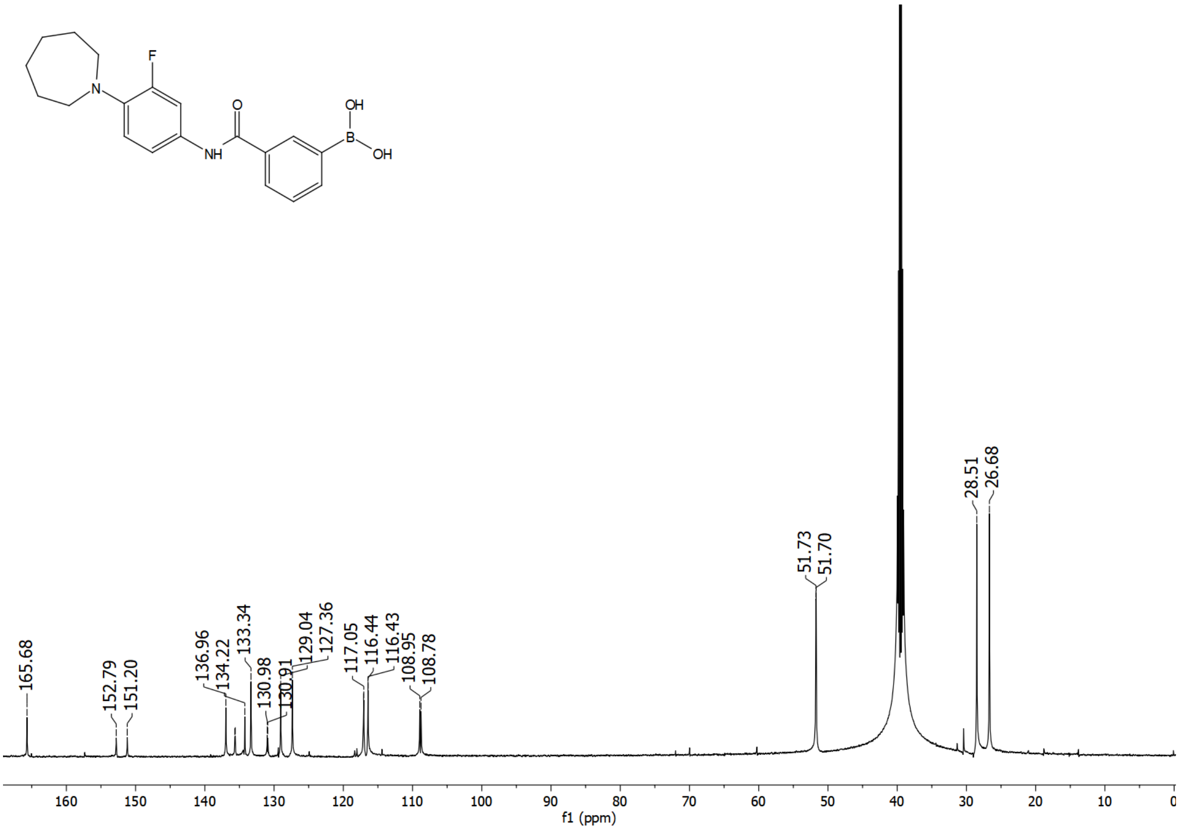

Figure S46: ^{13}C-NMR Spectrum of compound **34** (150 MHz DMSO-d_6).

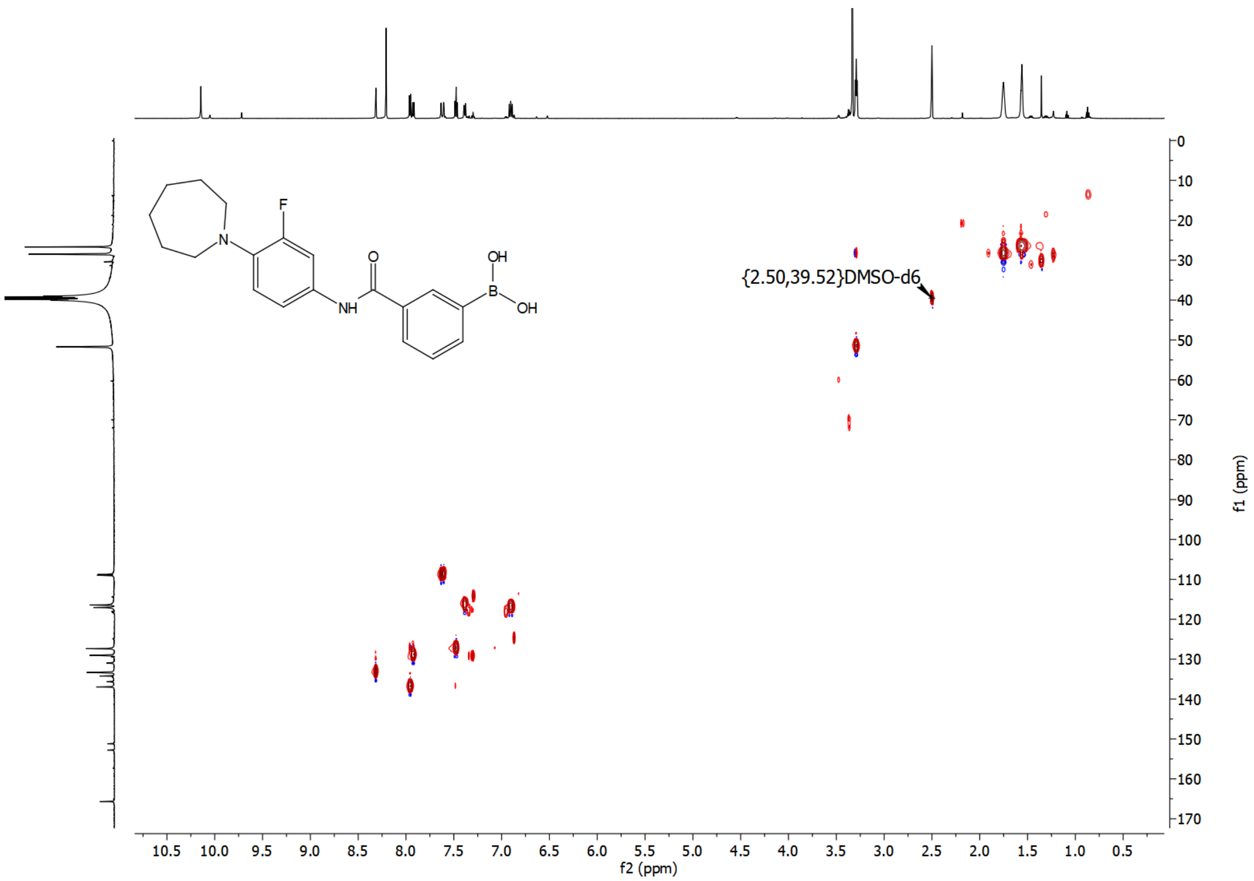

Figure S47: HSQC-NMR Spectrum of compound **34** (600 MHz DMSO-*d*6).

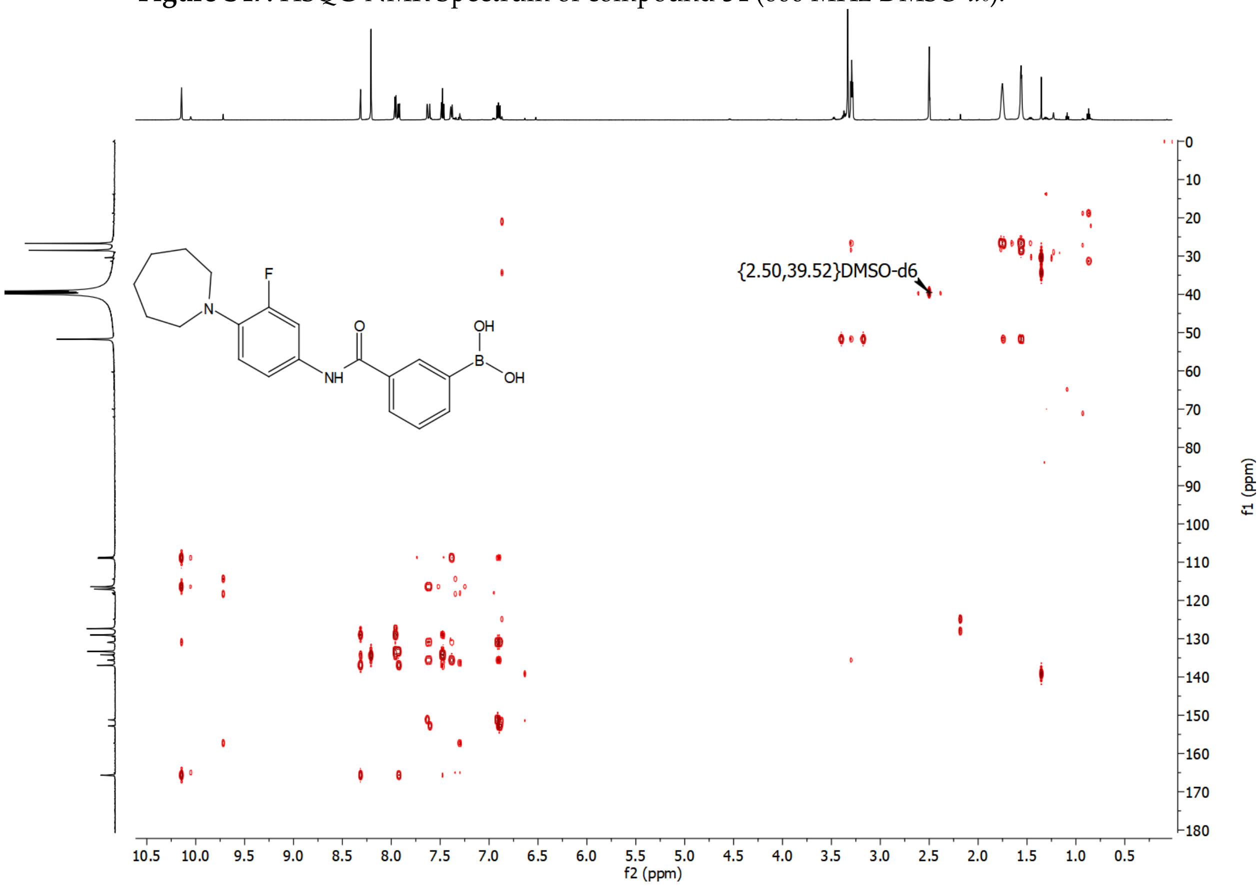

Figure S48: HMBC-NMR Spectrum of compound **34** (600 MHz DMSO-*d*6).

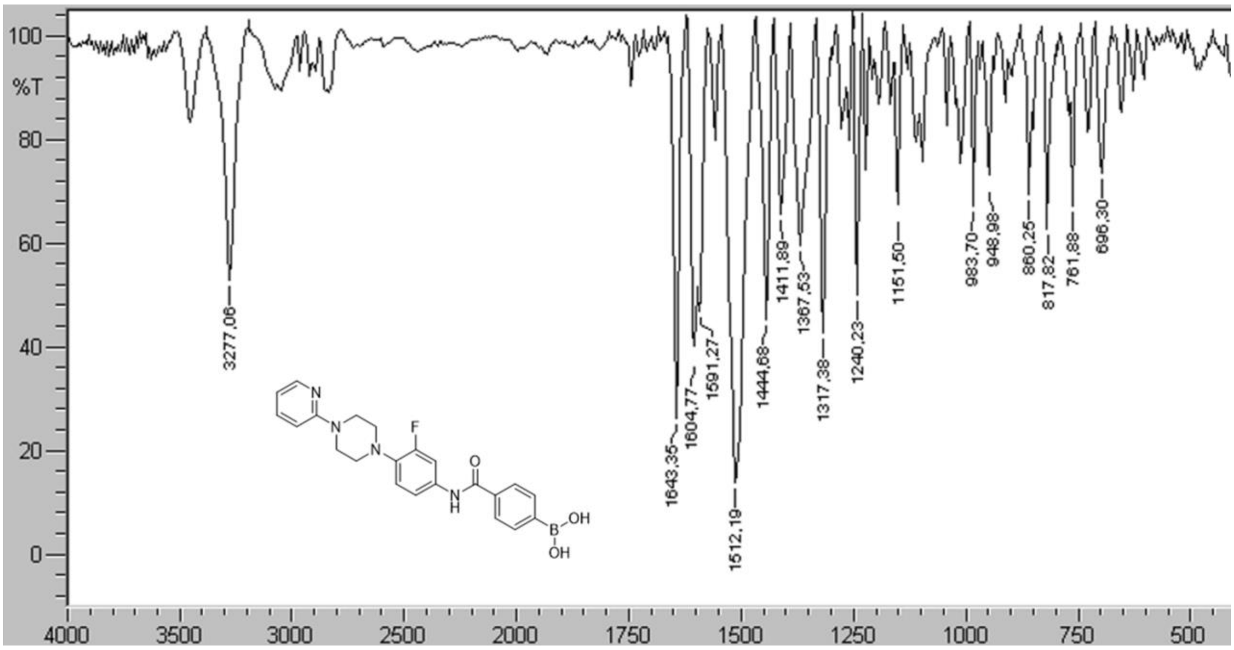

Figure S49: IR Spectrum of compound **35**.

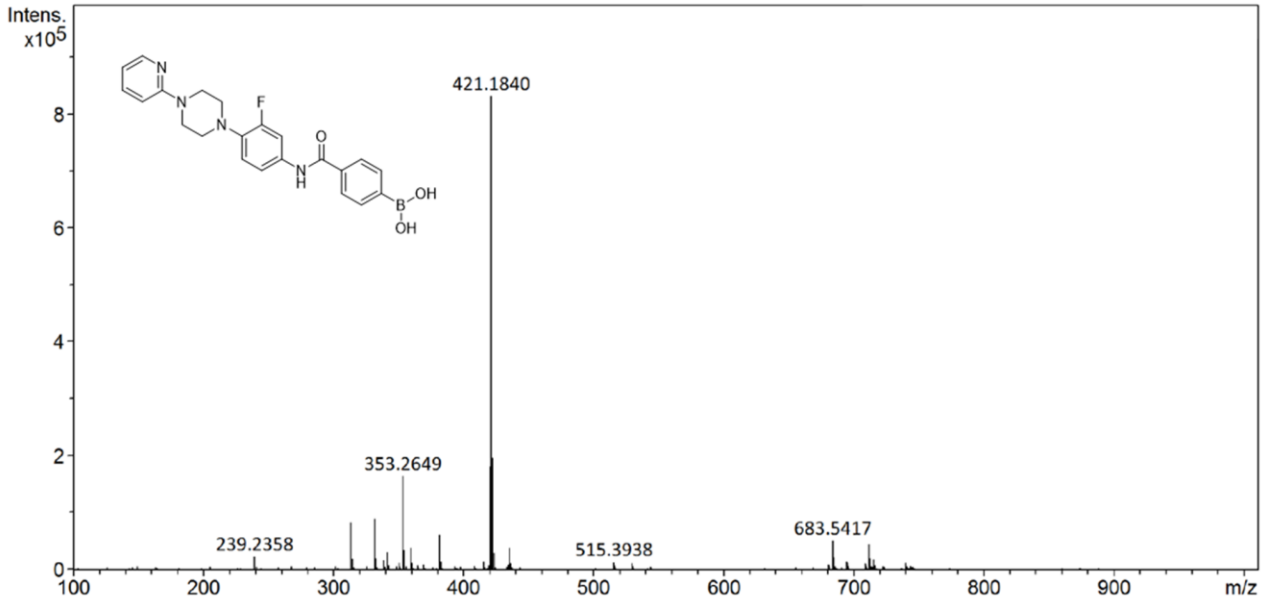

Figure S50: HRMS Spectrum of compound **35** (ESI+).

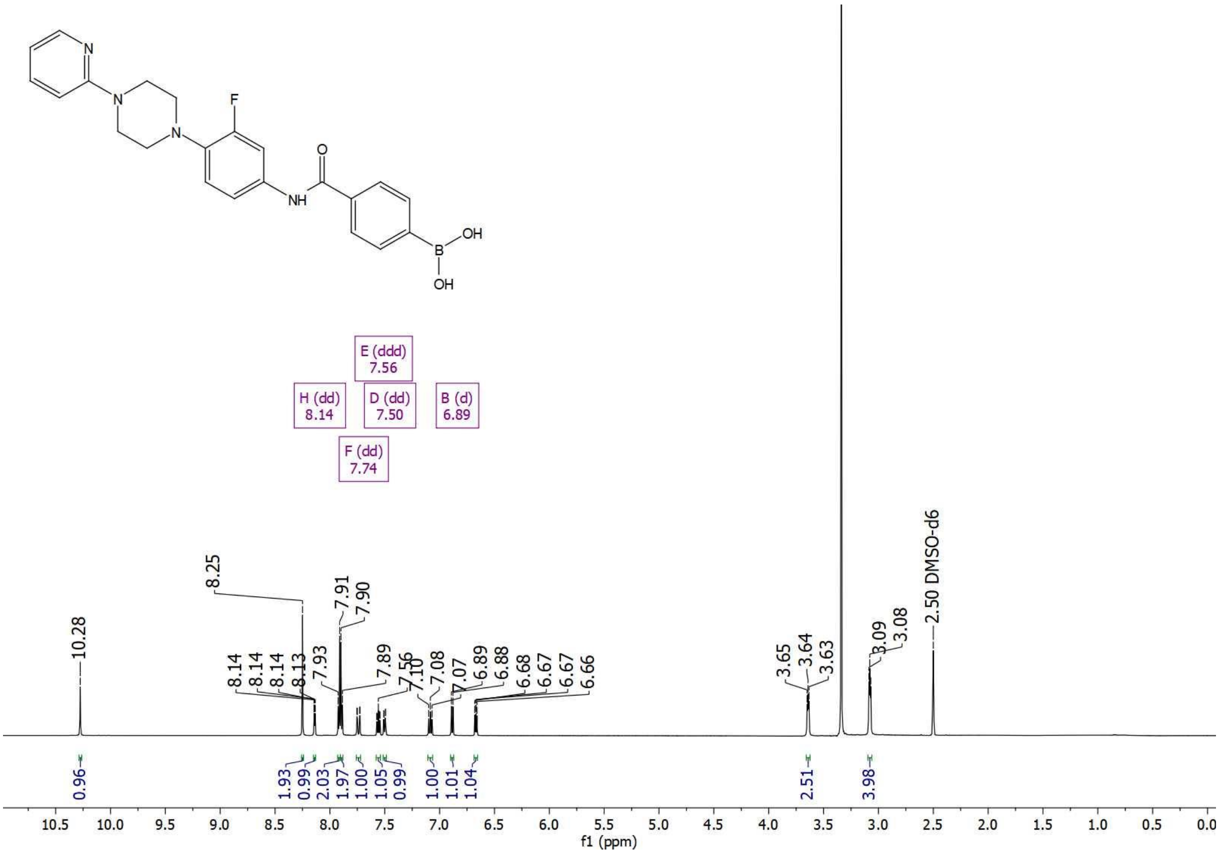

Figure S51: ^{1}H-NMR Spectrum of compound **35** (600 MHz DMSO-d_6).

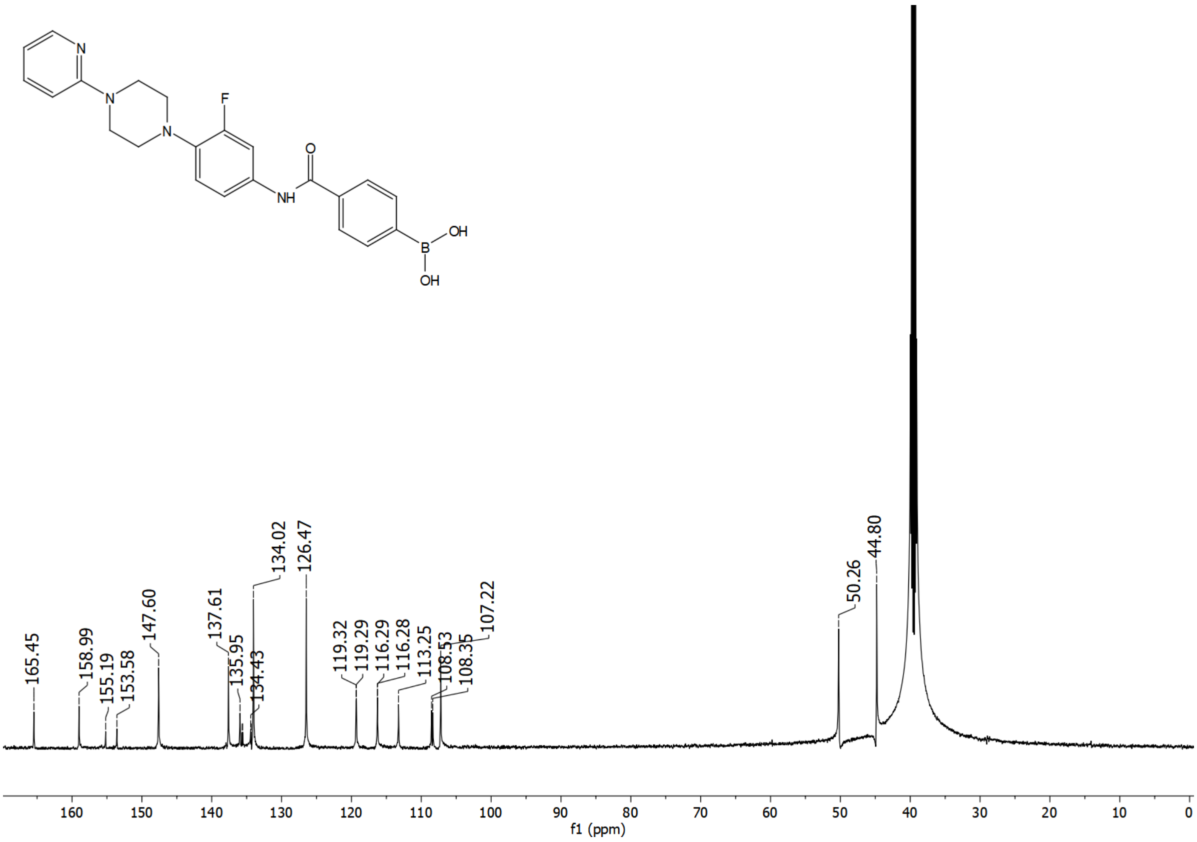

Figure S52: ^{13}C-NMR Spectrum of compound **35** (150 MHz DMSO-d_6).

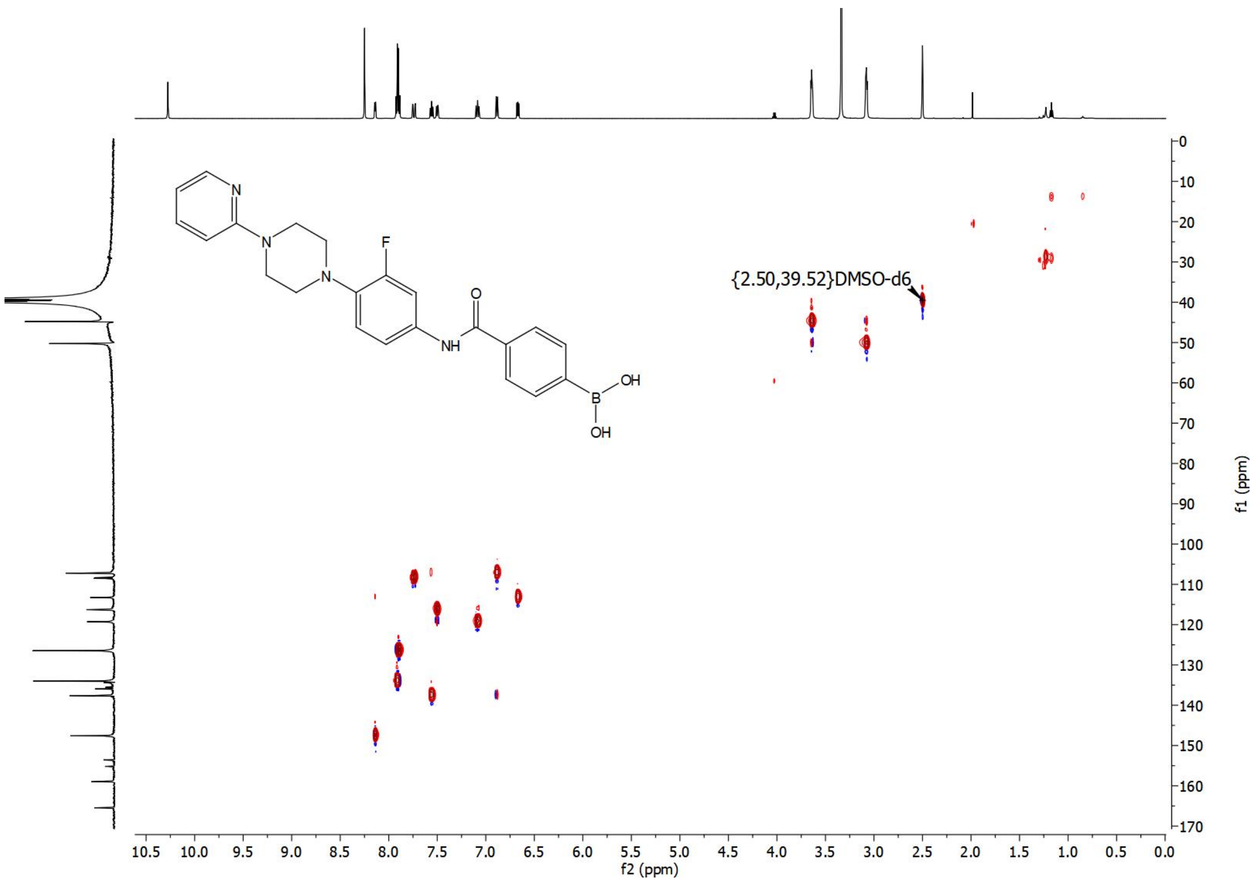

Figure S53: HSQC-NMR Spectrum of compound **35** (600 MHz DMSO-d_6).

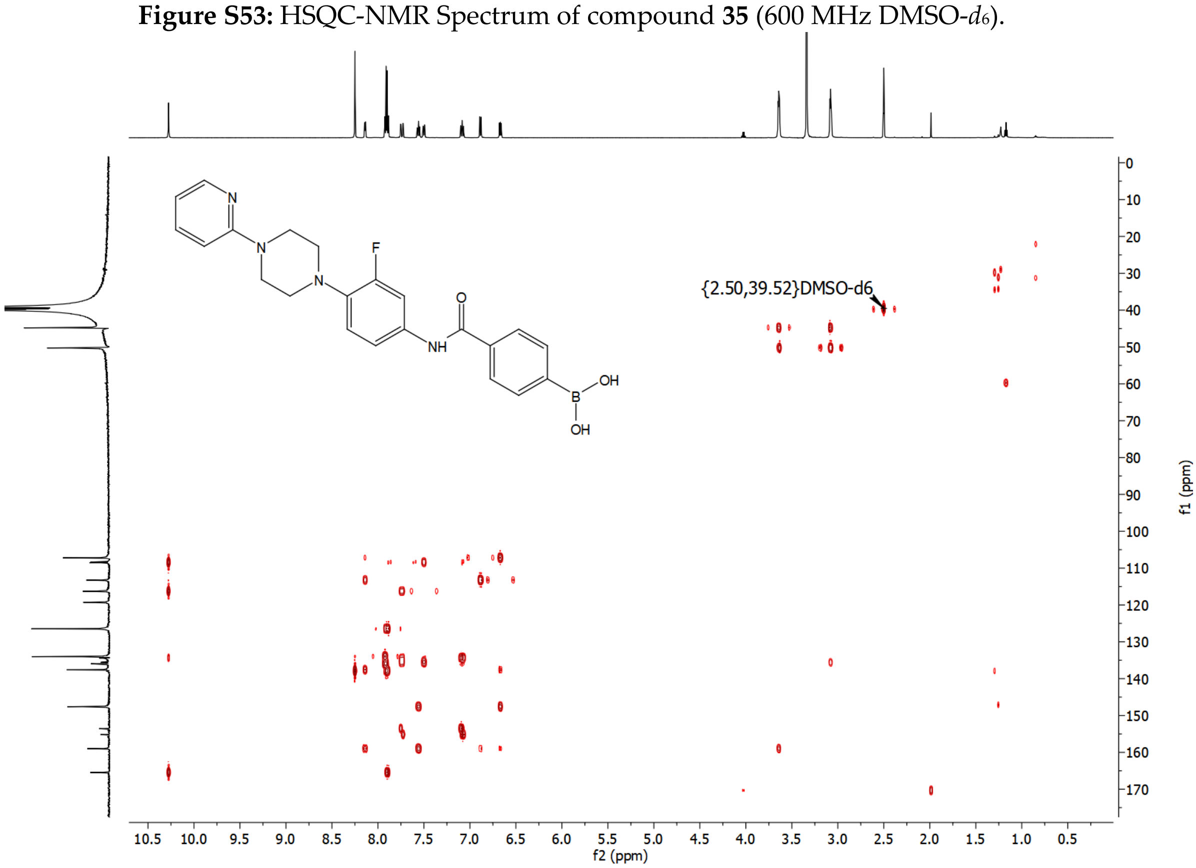

Figure S54: HMBC-NMR Spectrum of compound **35** (600 MHz DMSO-d_6).

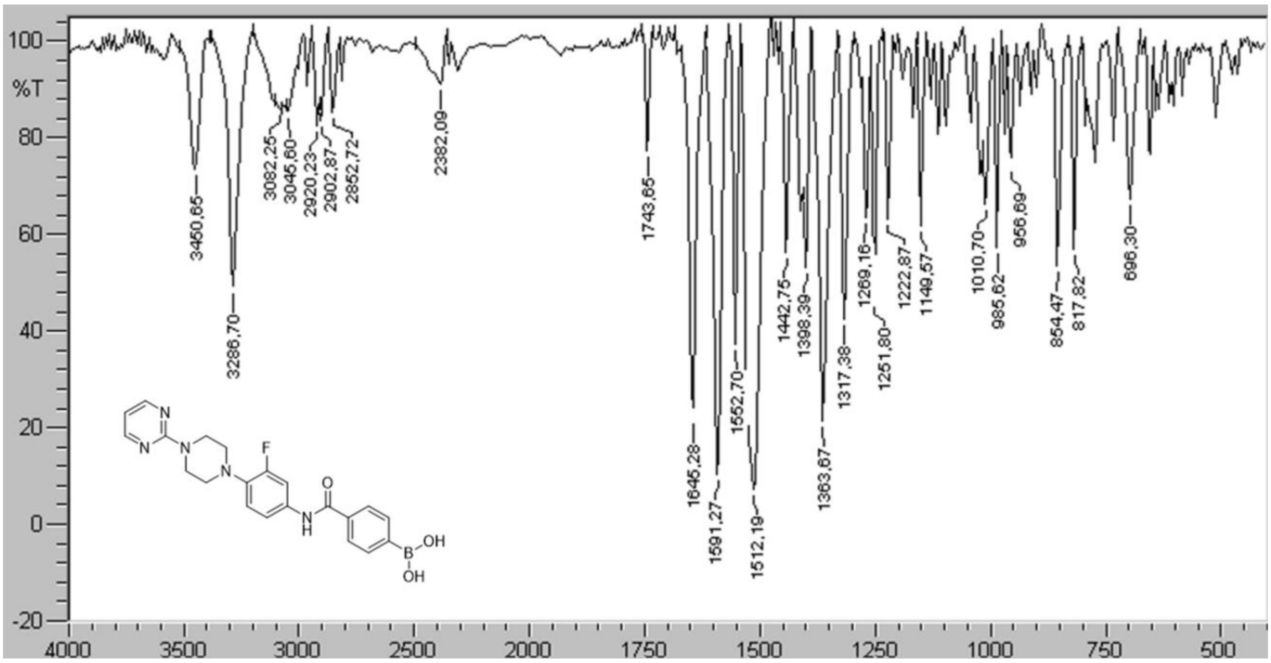

Figure S55: IR Spectrum of compound **36**.

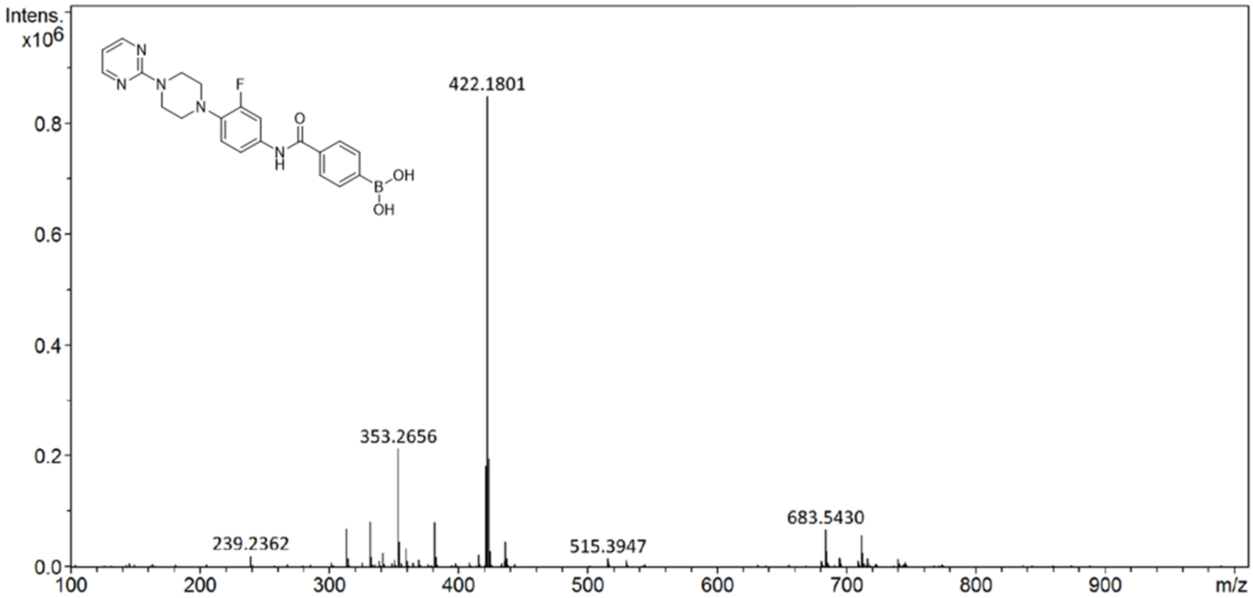

Figure S56: HRMS Spectrum of compound **36** (ESI$^+$).

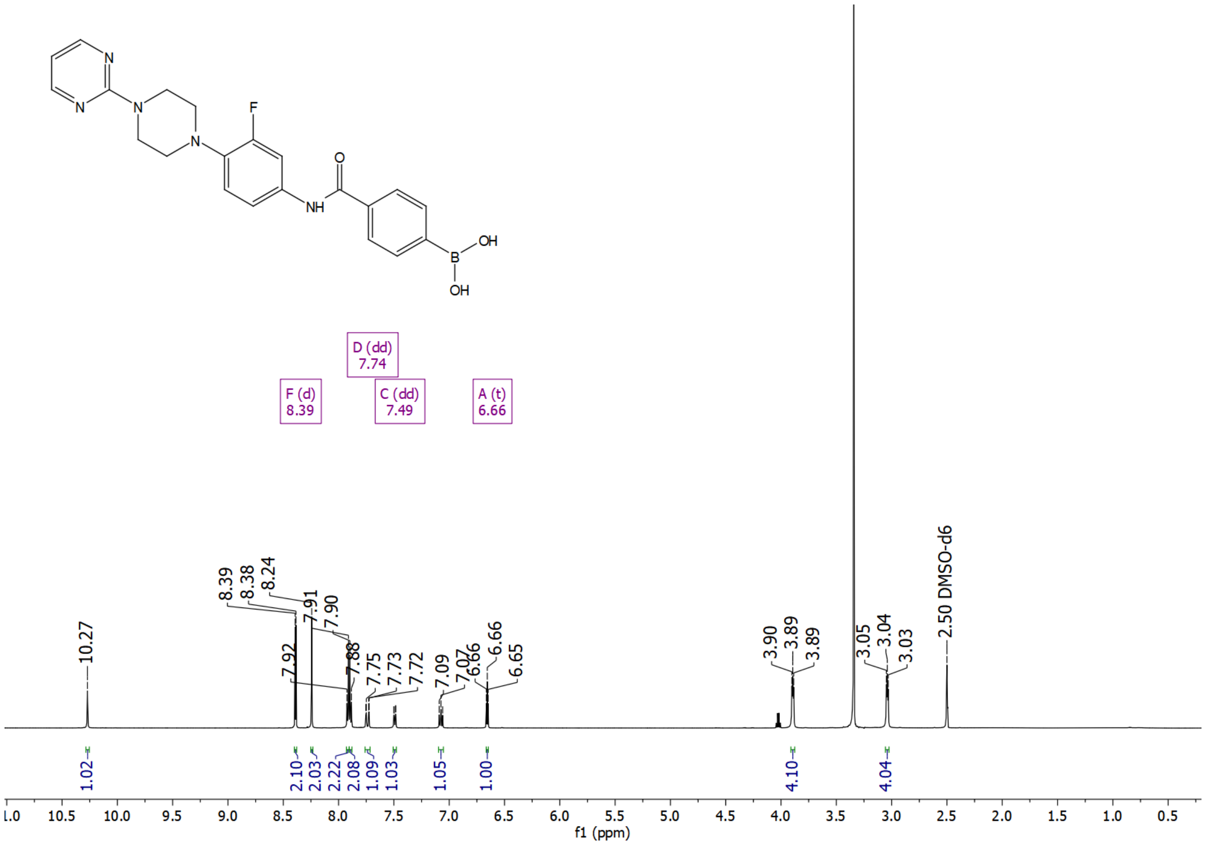

Figure S57: ^{1}H-NMR Spectrum of compound **36** (600 MHz DMSO-d_6).

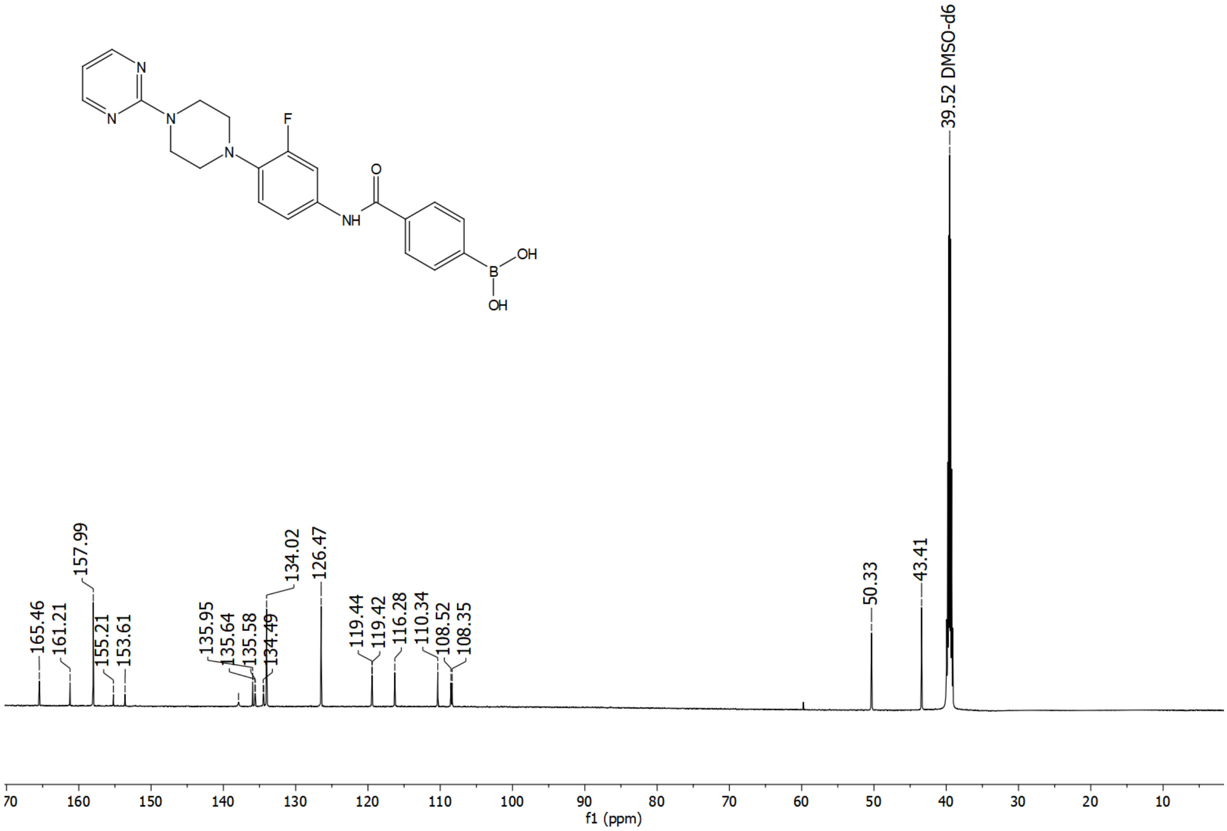

Figure S58: ^{13}C-NMR Spectrum of compound **36** (150 MHz DMSO-d_6).

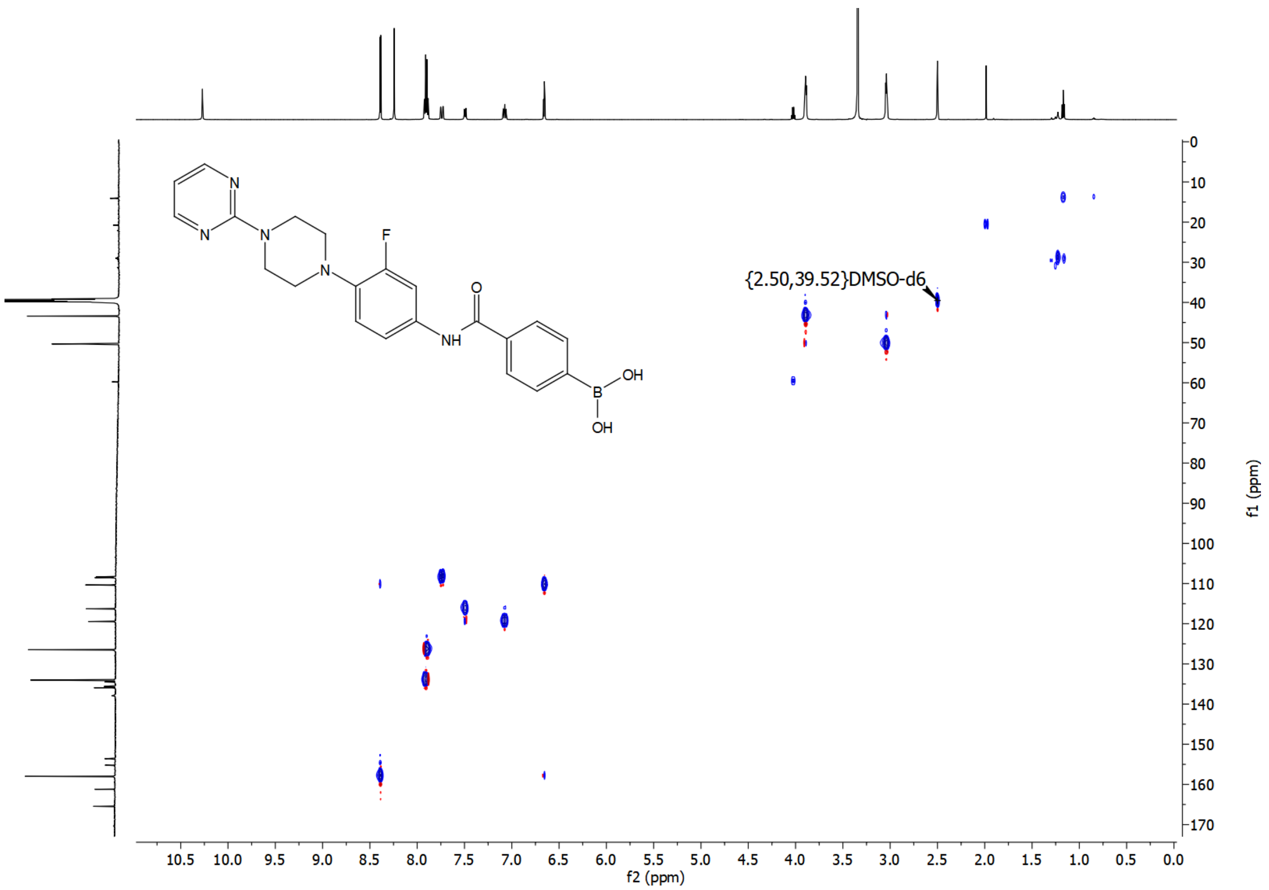

Figure S59: HSQC-NMR Spectrum of compound **36** (600 MHz DMSO-*d*6).

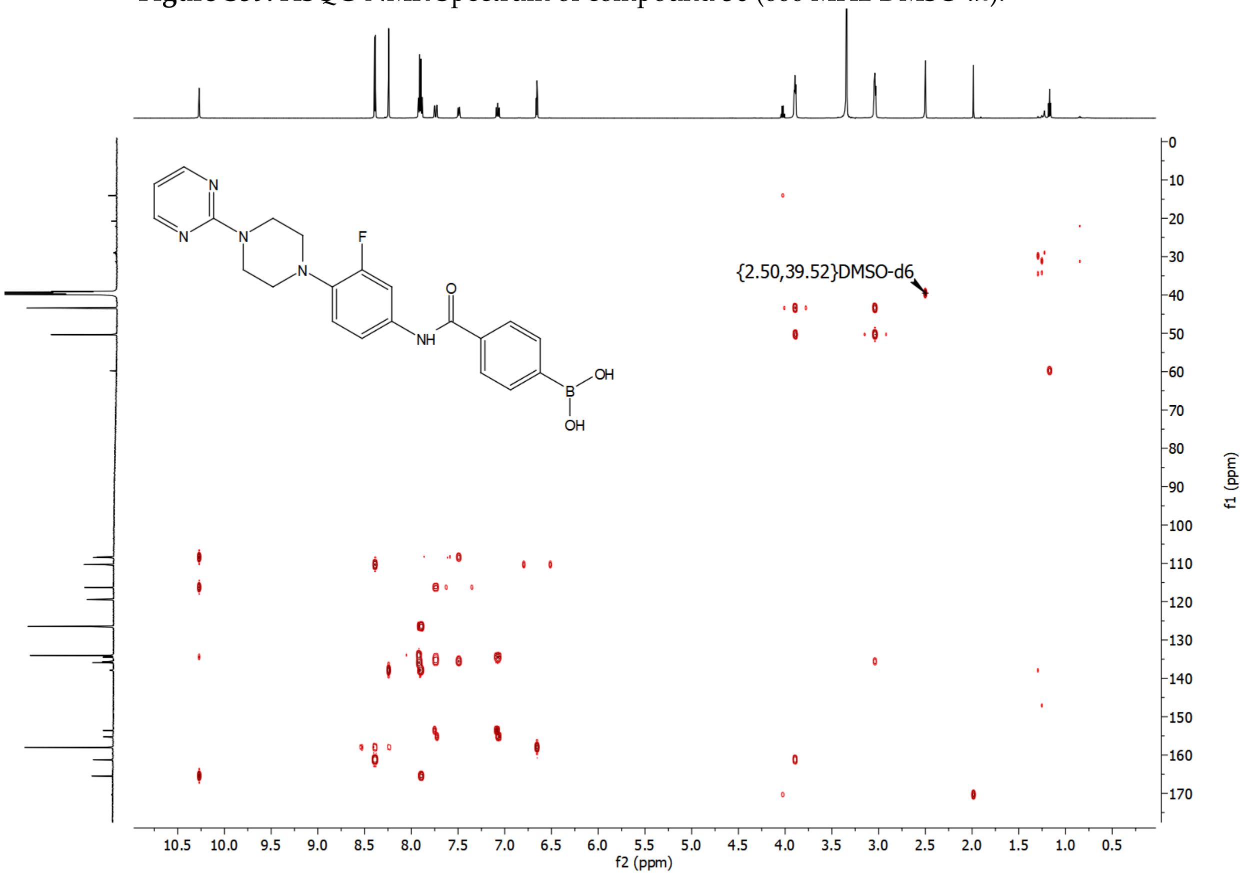

Figure S60: HMBC-NMR Spectrum of compound **36** (600 MHz DMSO-*d*6).

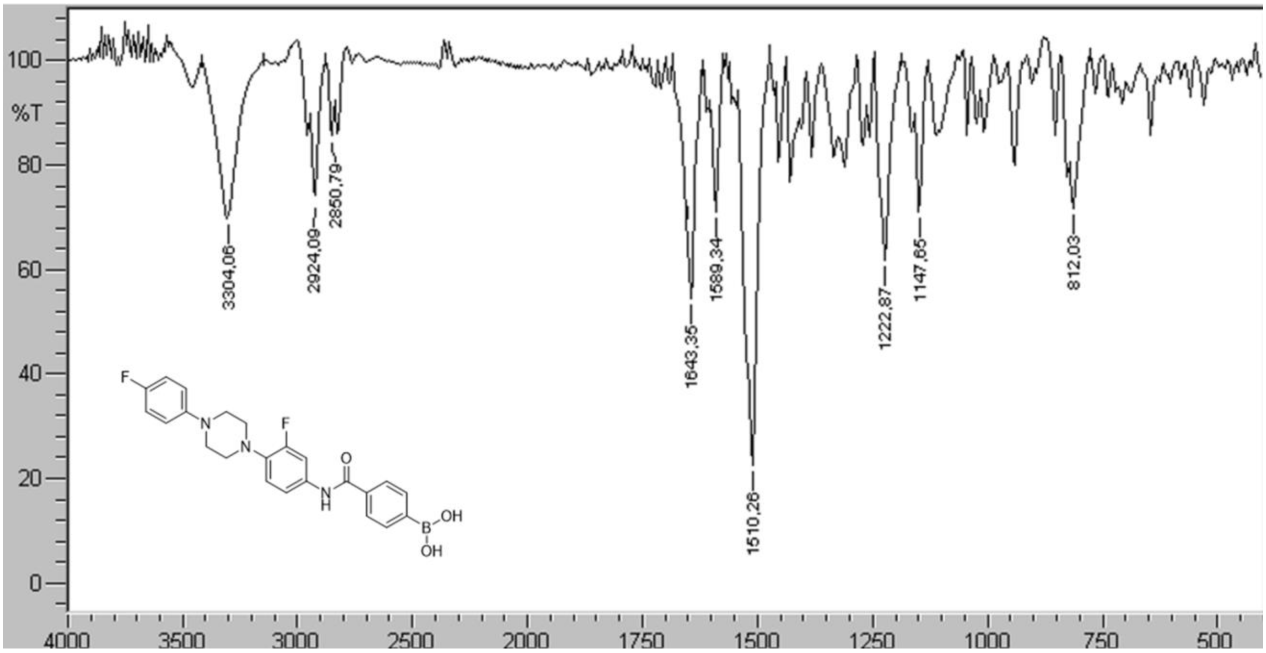

Figure S61: IR Spectrum of compound **37**.

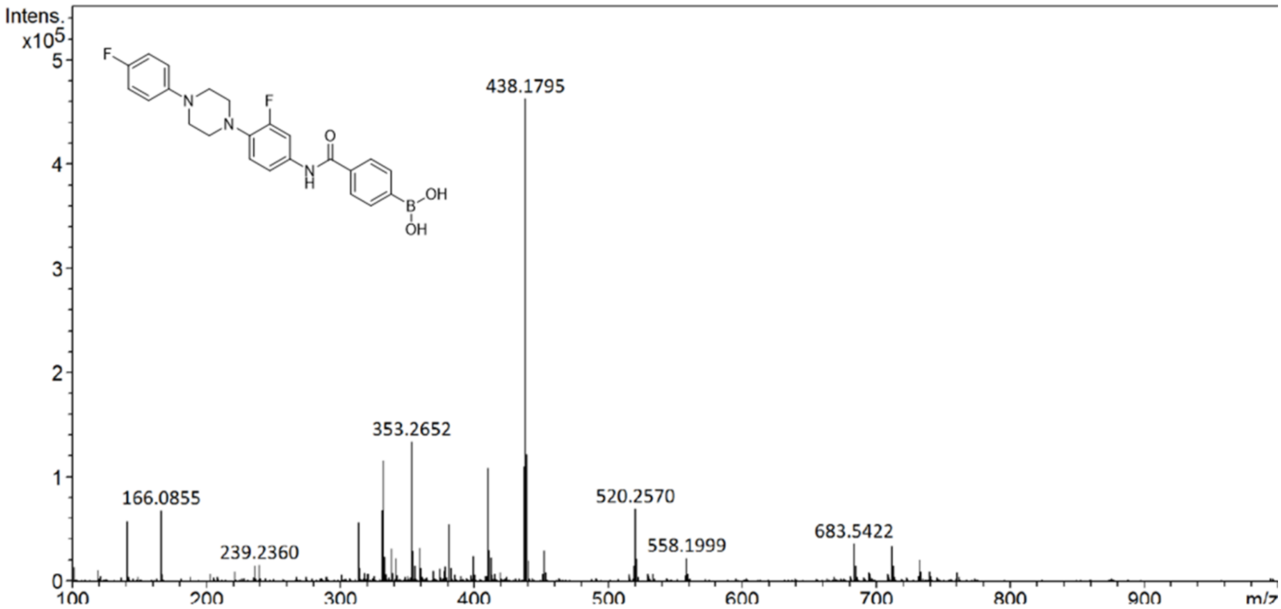

Figure S62: HRMS Spectrum of compound **37** (ESI$^+$).

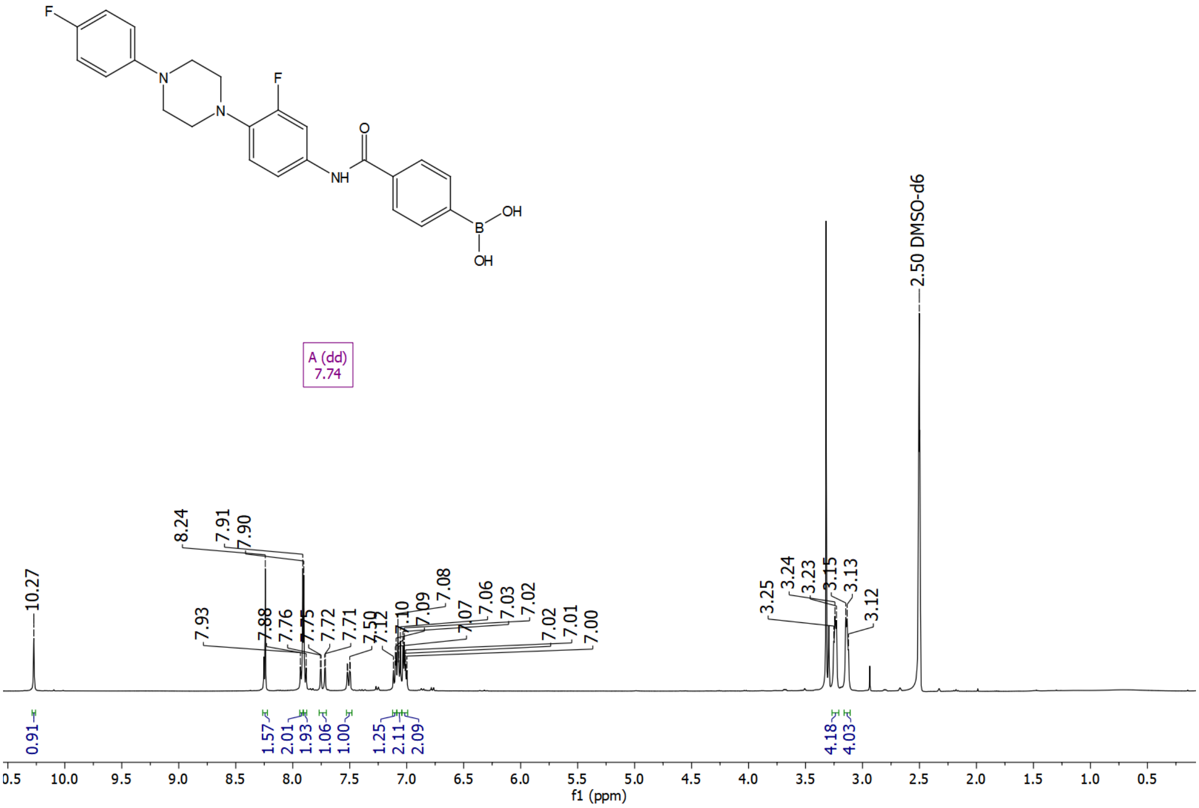

Figure S63: ^{1}H-NMR Spectrum of compound **37** (600 MHz DMSO-d_6).

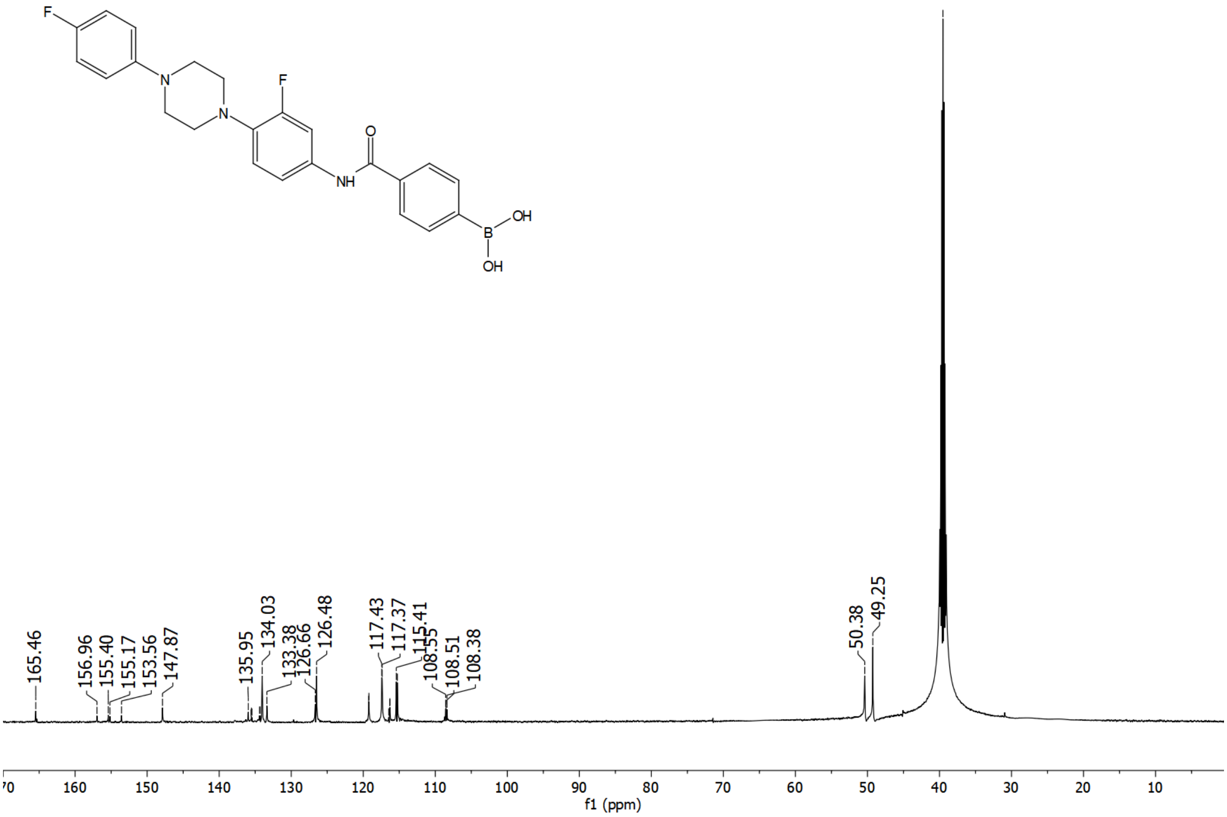

Figure S64: ^{13}C-NMR Spectrum of compound **37** (150 MHz DMSO-d_6).

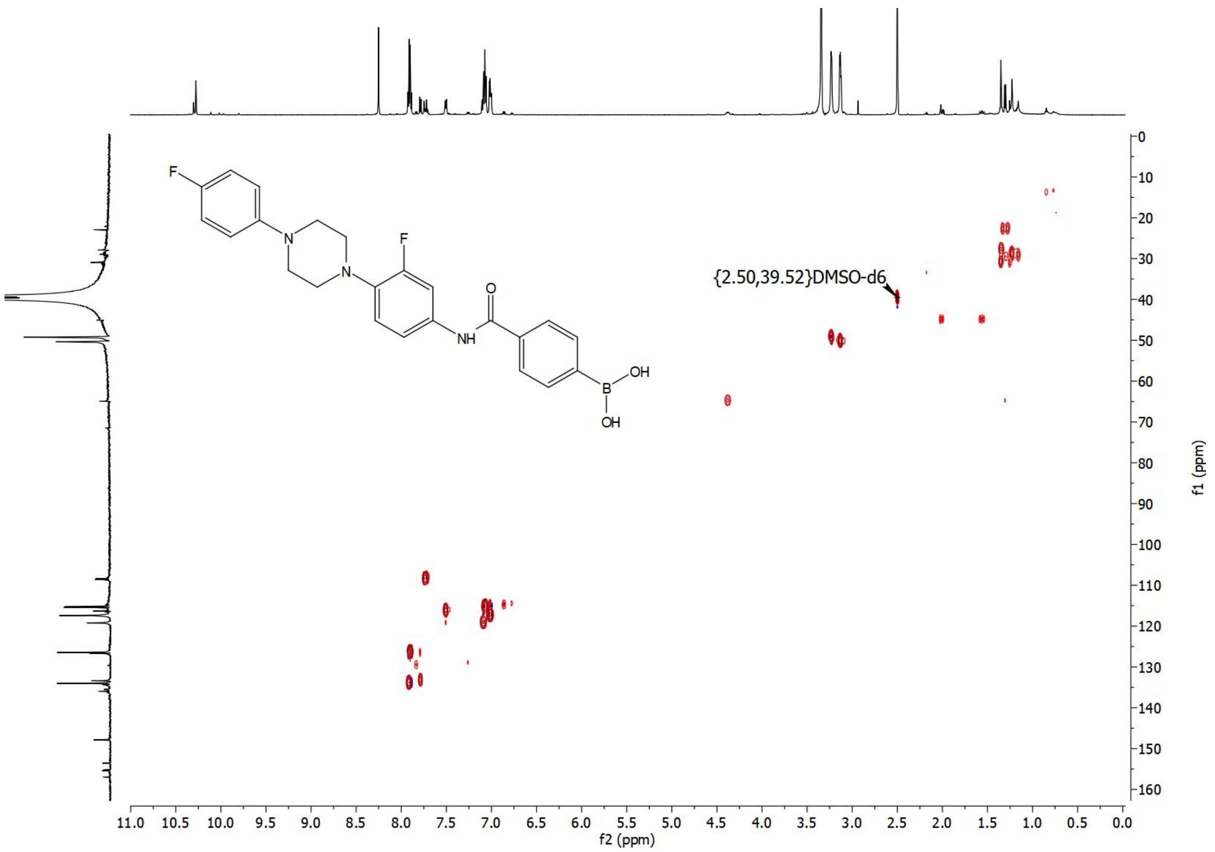

Figure S65: HSQC-NMR Spectrum of compound **37** (600 MHz DMSO-d_6).

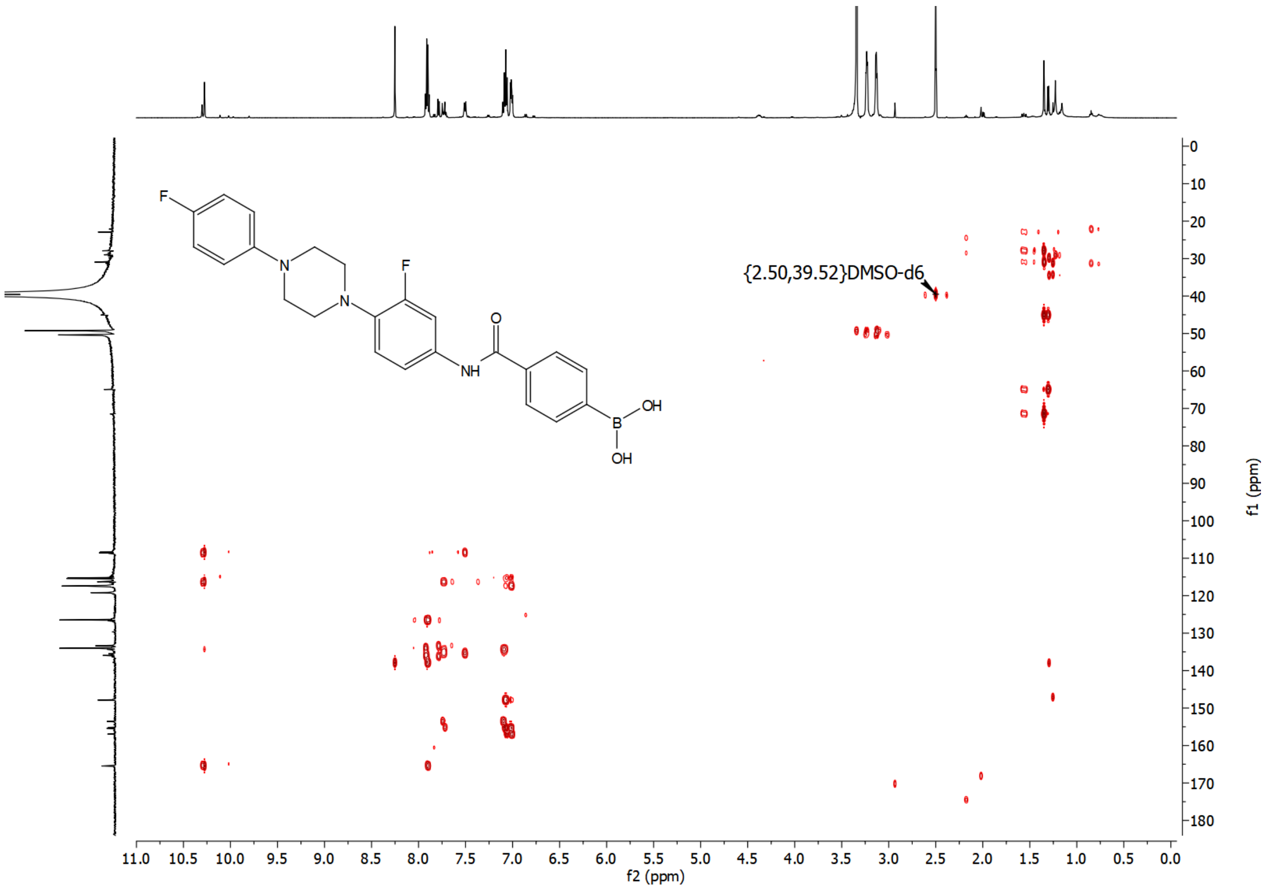

Figure S66: HMBC-NMR Spectrum of compound **37** (600 MHz DMSO-d_6).

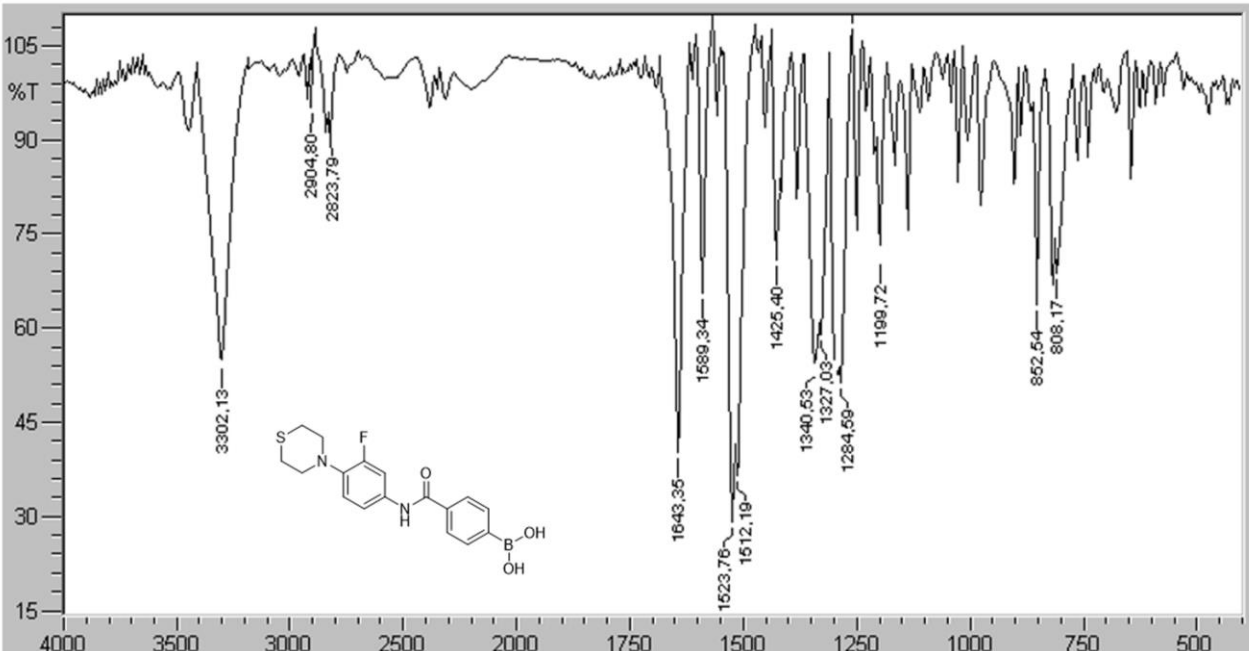

Figure S67: IR Spectrum of compound **38**.

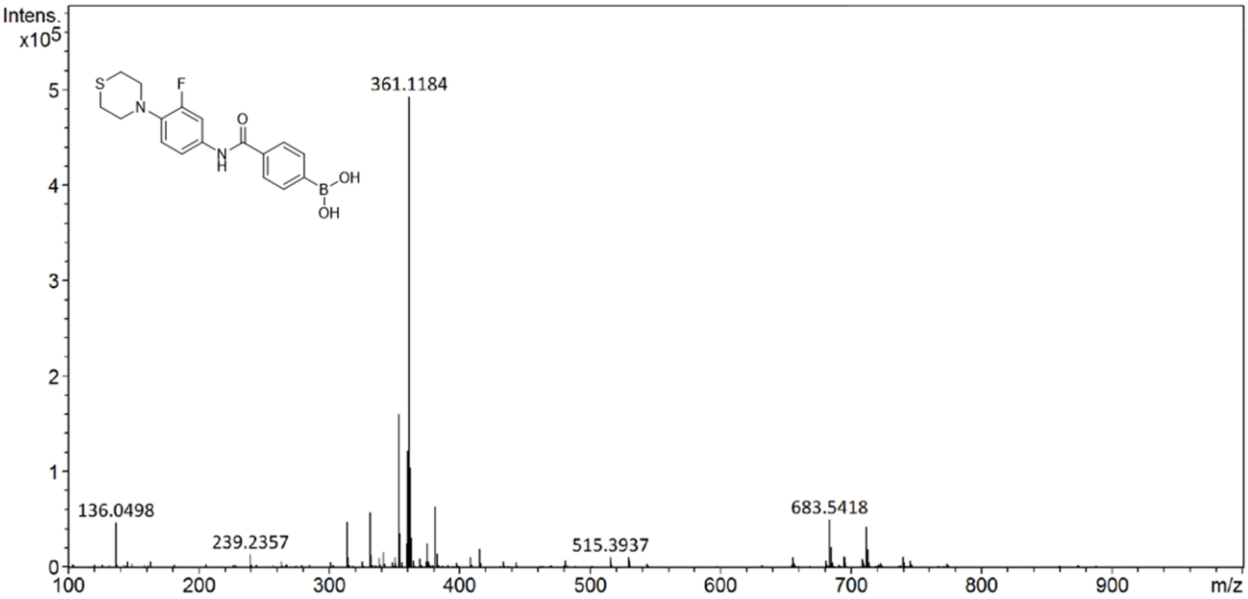

Figure S68: HRMS Spectrum of compound **38** (ESI+).

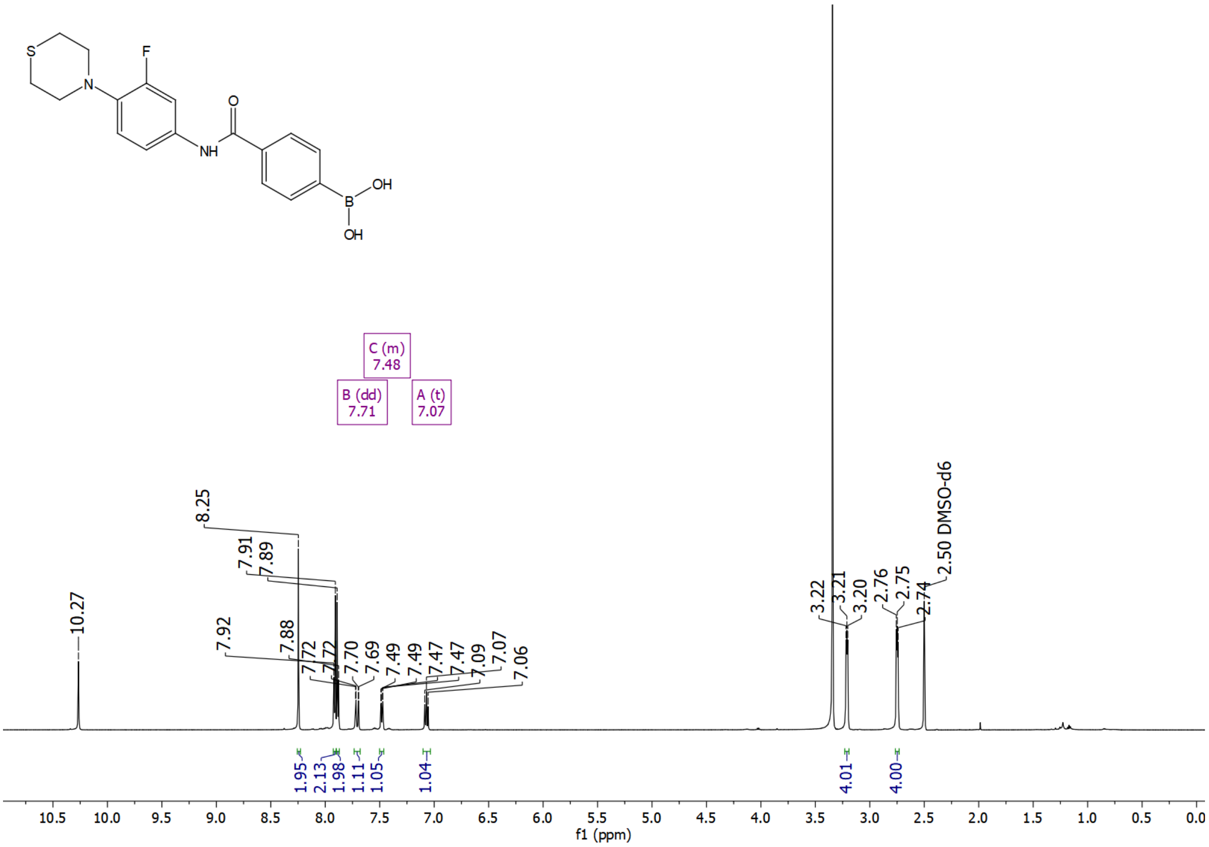

Figure S69: ^{1}H-NMR Spectrum of compound **38** (600 MHz DMSO-*d*$_6$).

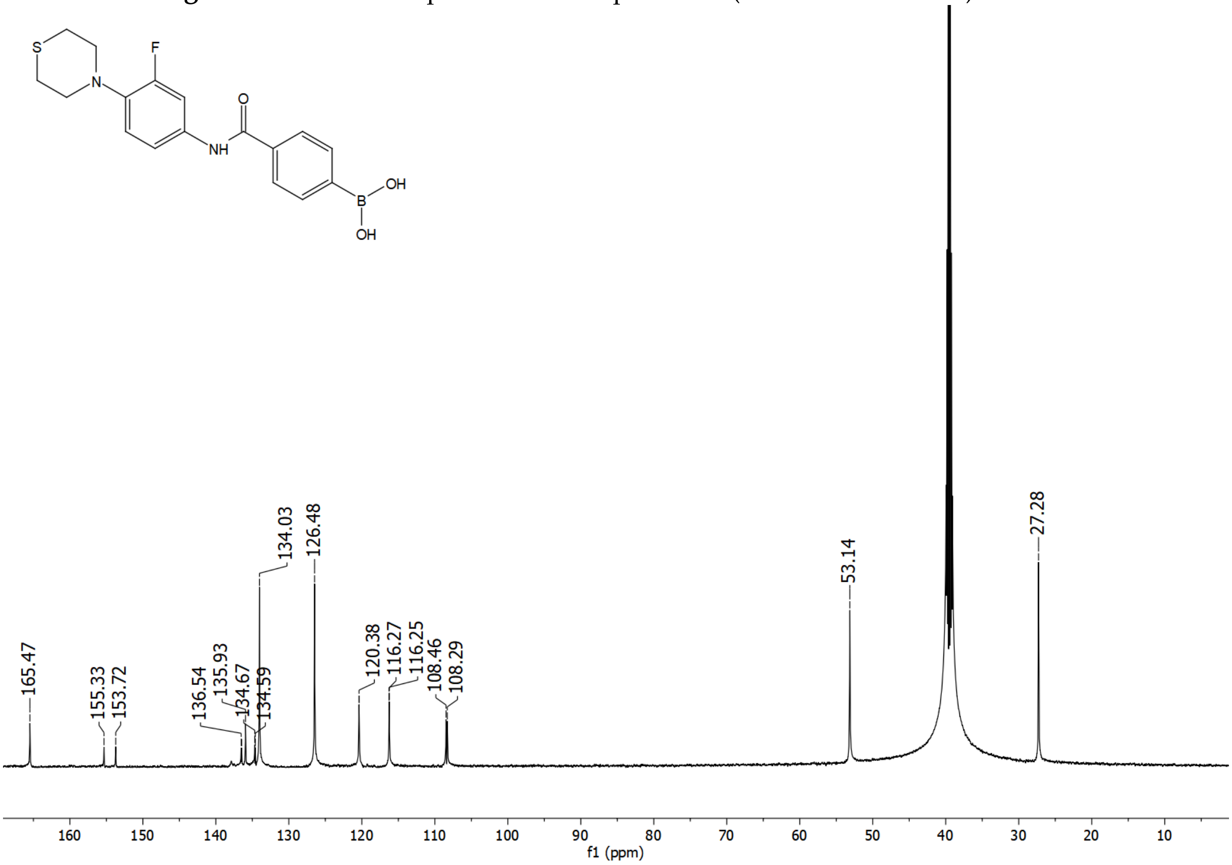

Figure S70: ^{13}C-NMR Spectrum of compound **38** (150 MHz DMSO-*d*$_6$).

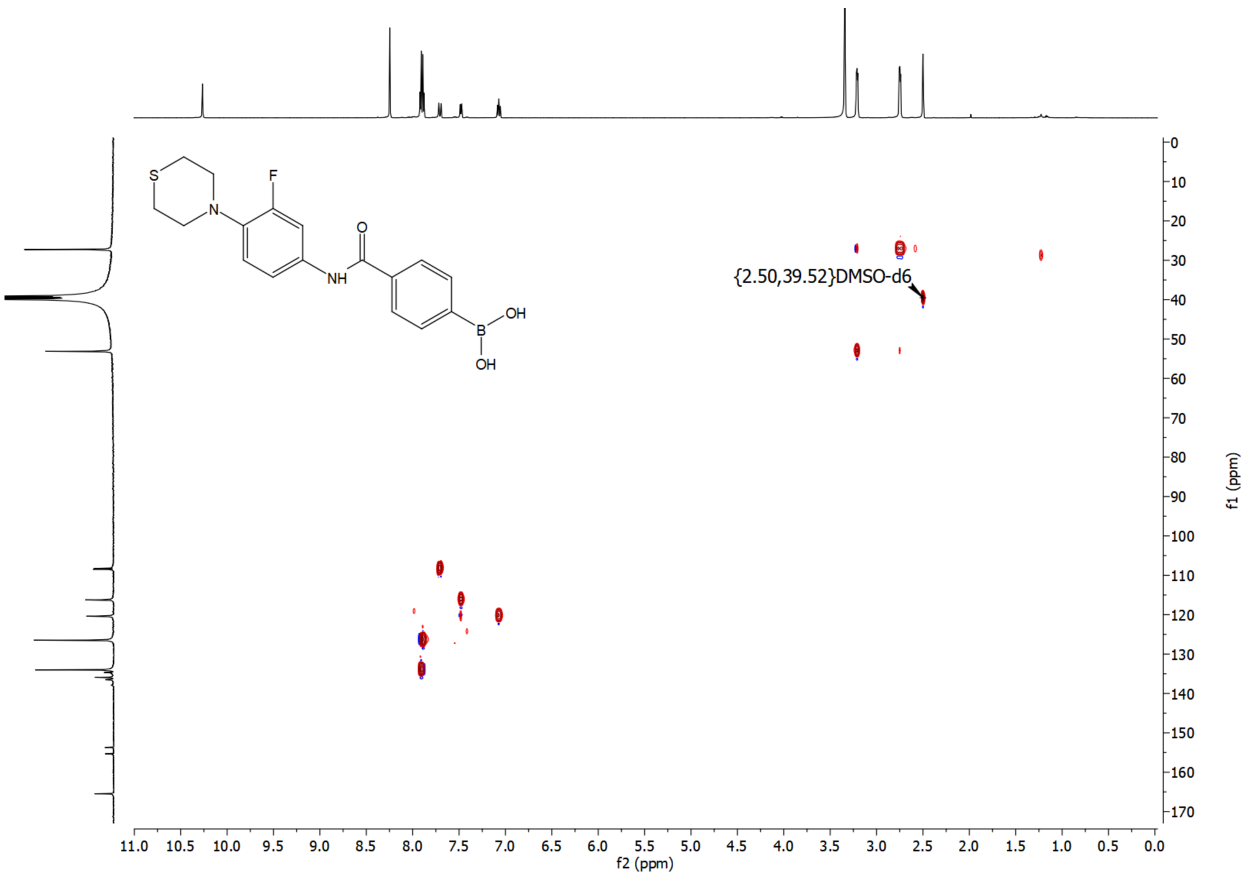

Figure S71: HSQC-NMR Spectrum of compound **38** (600 MHz DMSO-d_6).

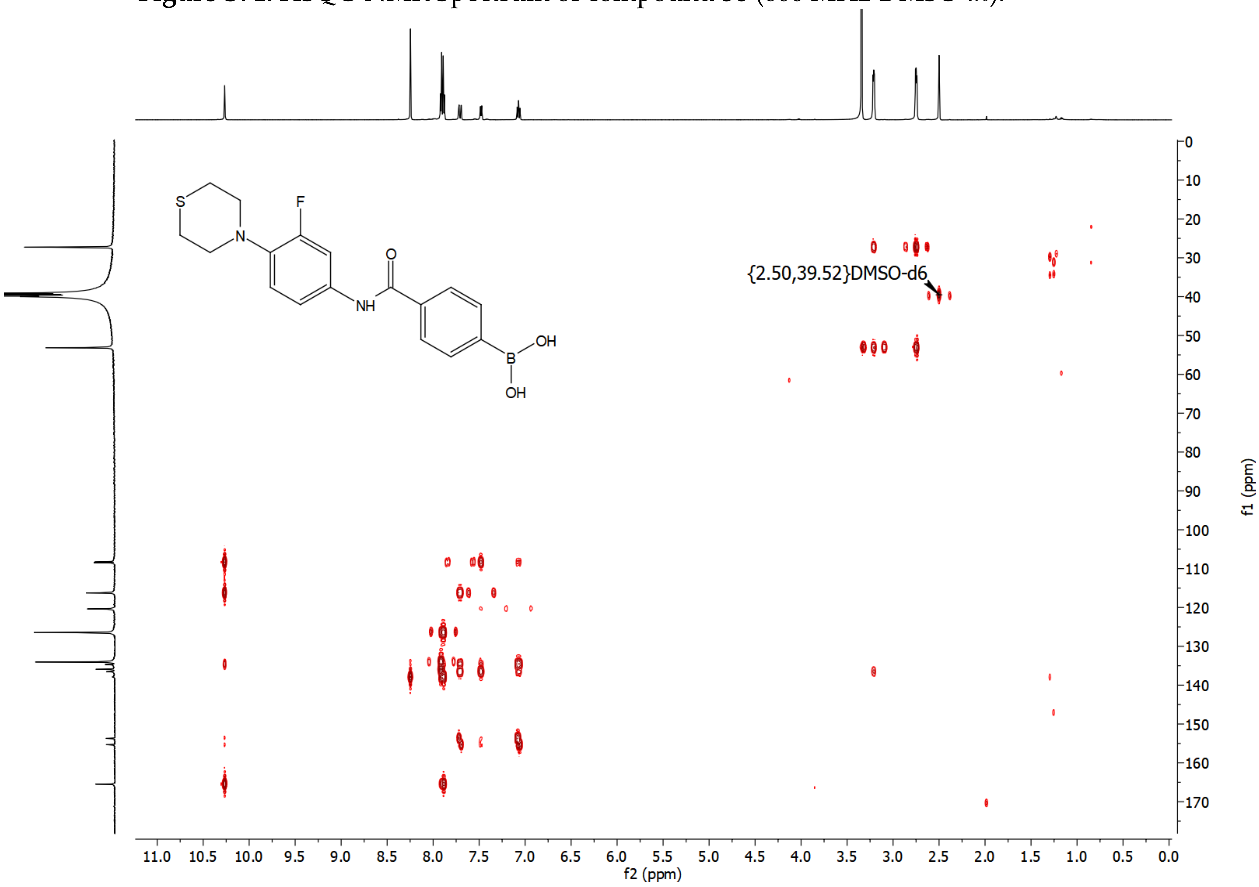

Figure S72: HMBC-NMR Spectrum of compound **38** (600 MHz DMSO-d_6).

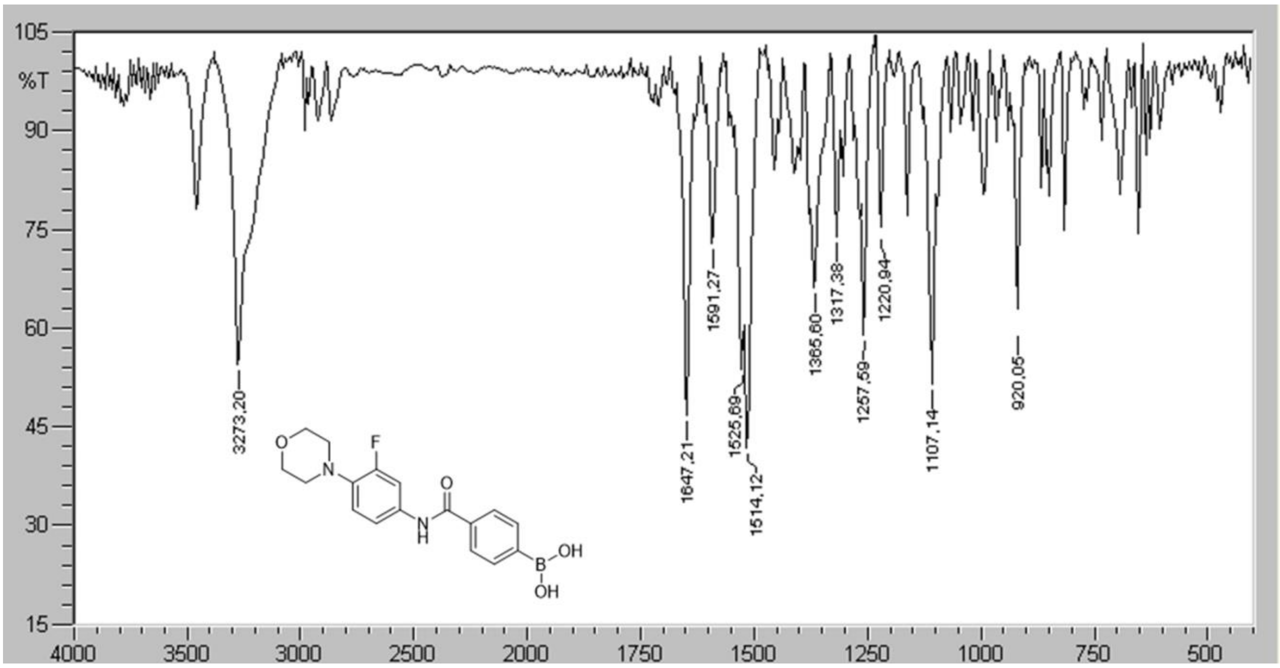

Figure S73: IR Spectrum of compound **39**.

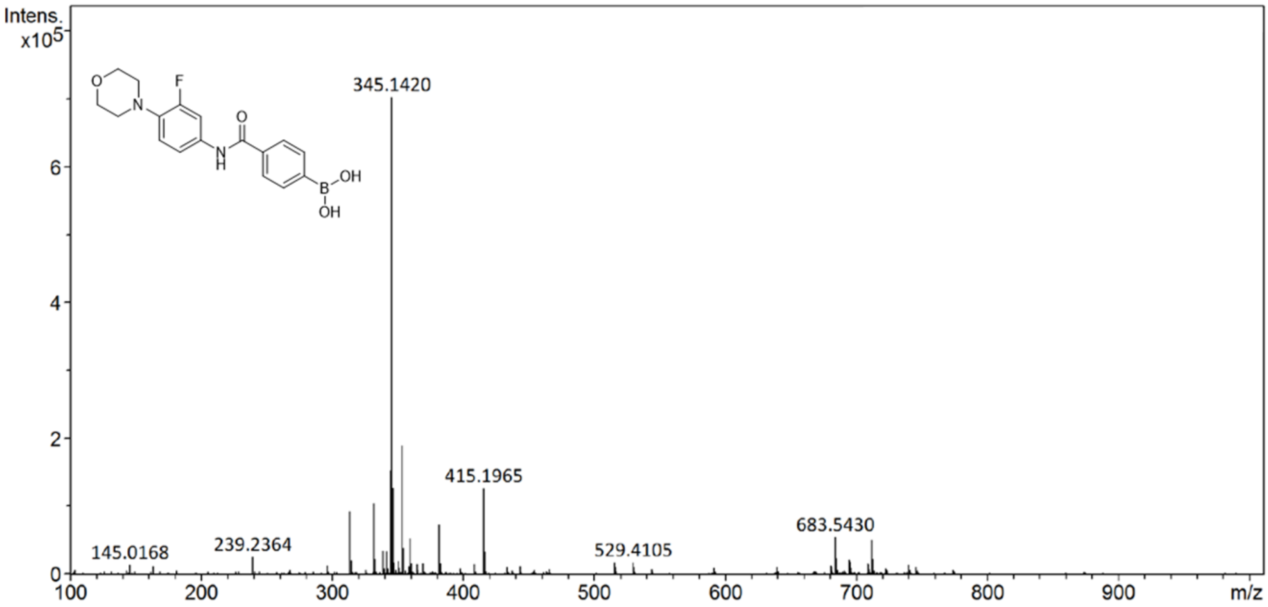

Figure S74: HRMS Spectrum of compound **39** (ESI+).

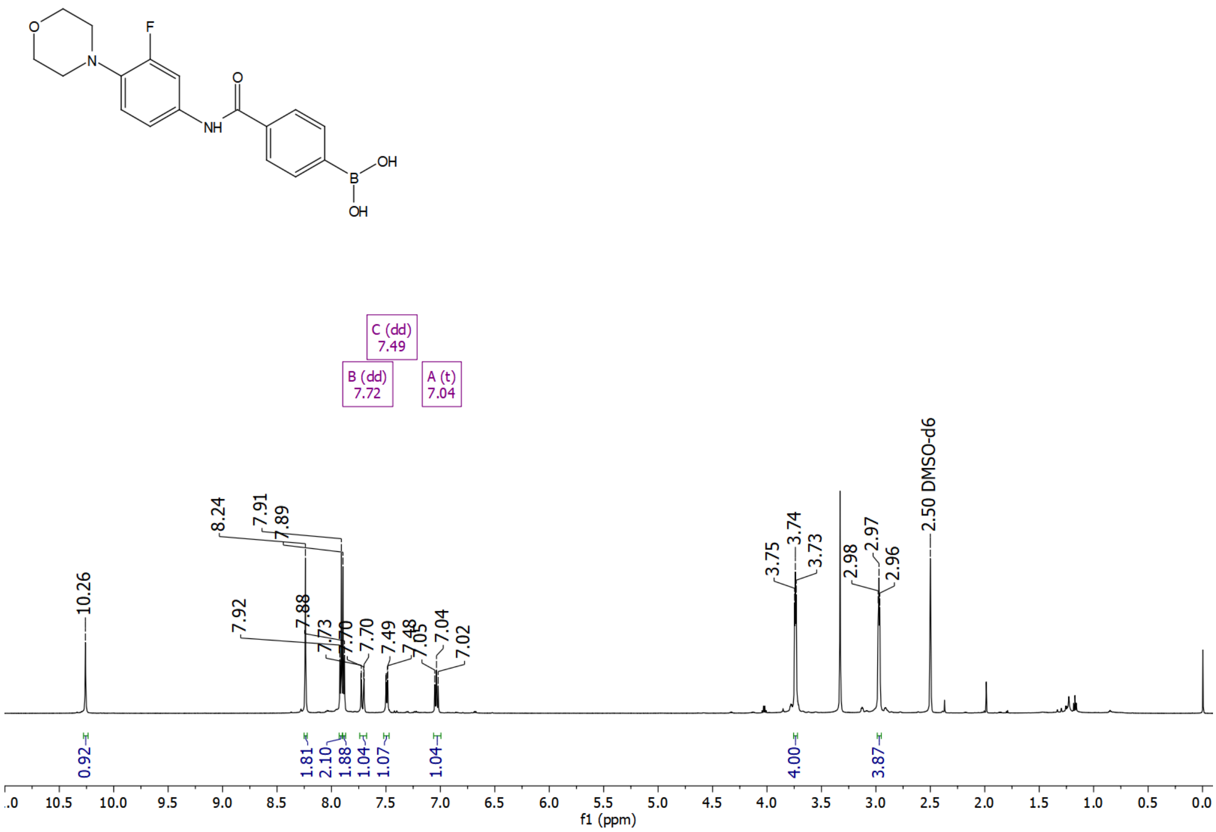

Figure S75: ^{1}H-NMR Spectrum of compound **39** (600 MHz DMSO-d_6).

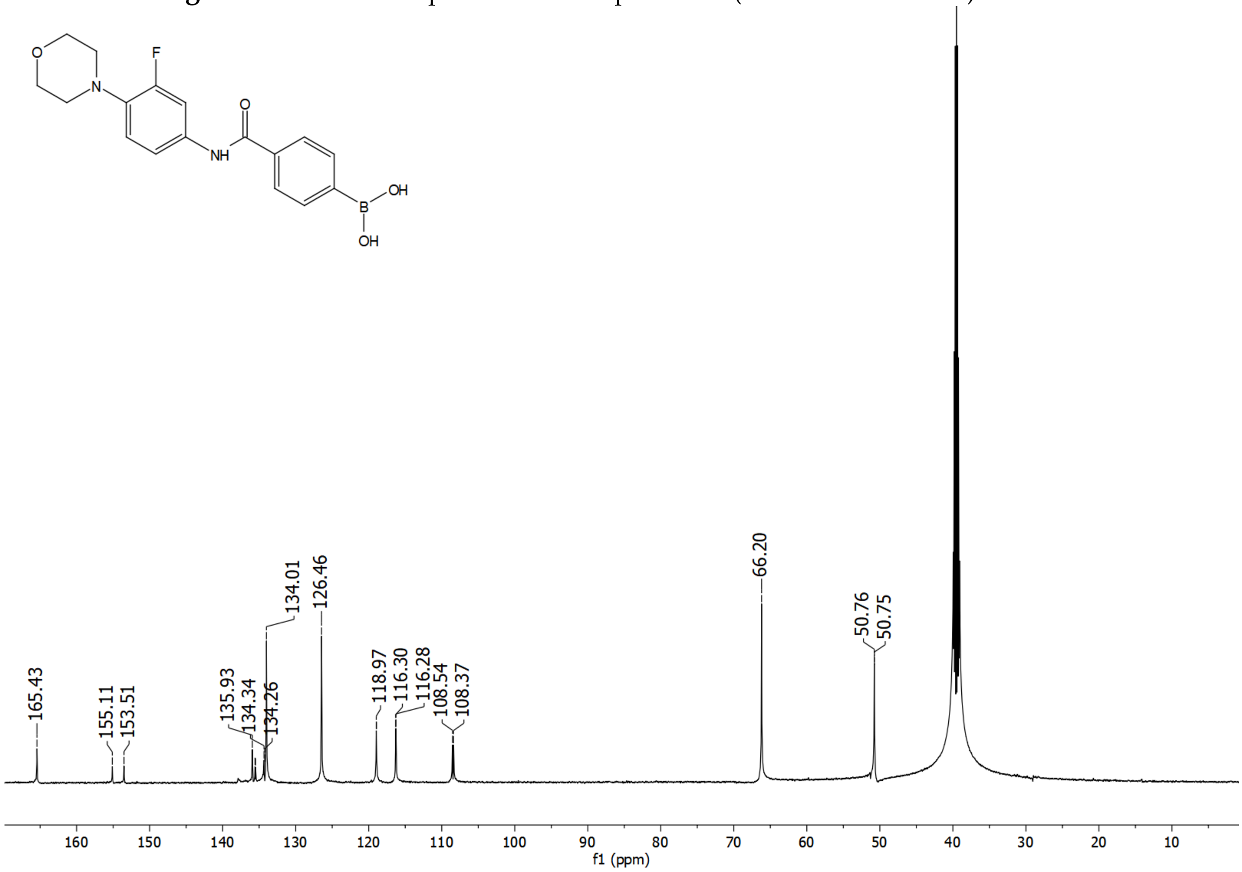

Figure S76: ^{13}C-NMR Spectrum of compound **39** (ESI^{+}).

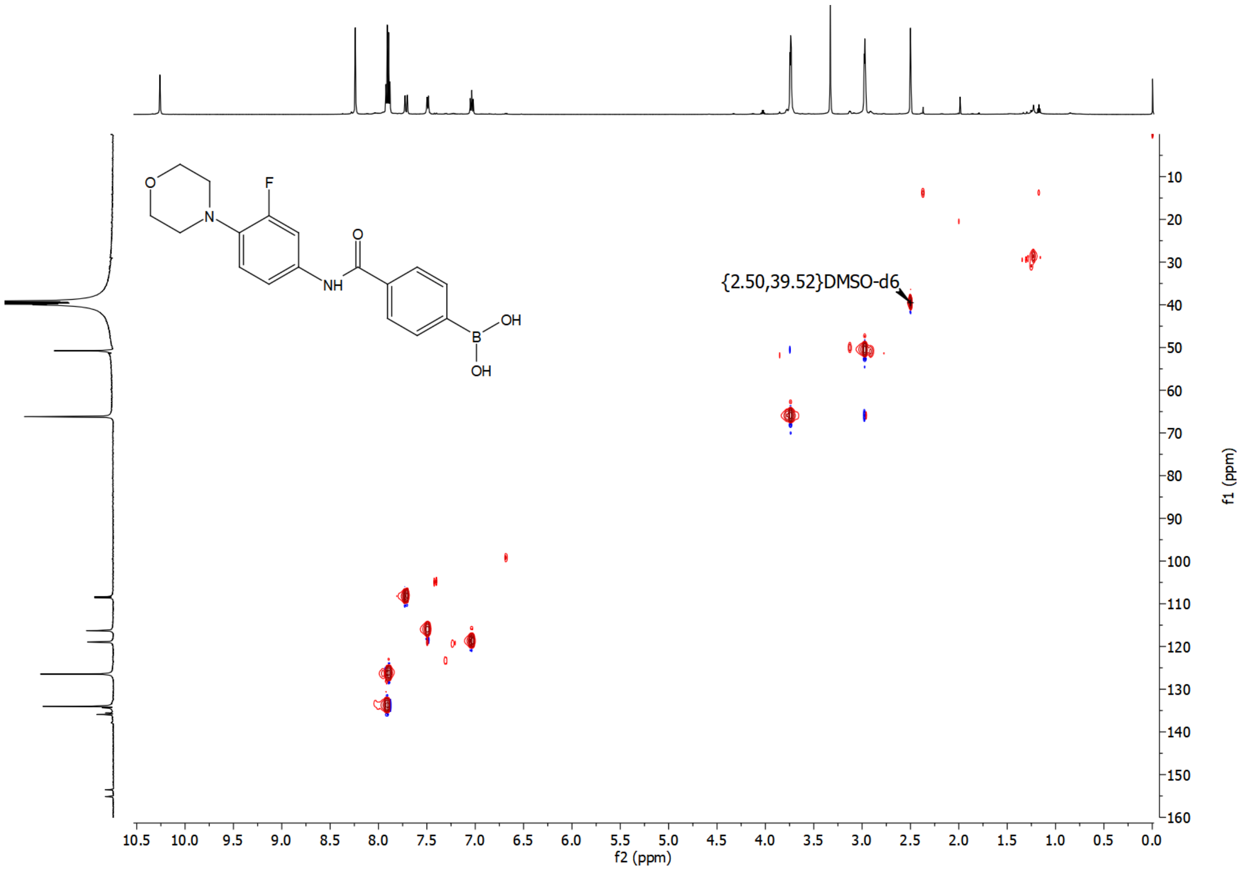

Figure S77: HSQC-NMR Spectrum of compound **39** (600 MHz DMSO-d_6).

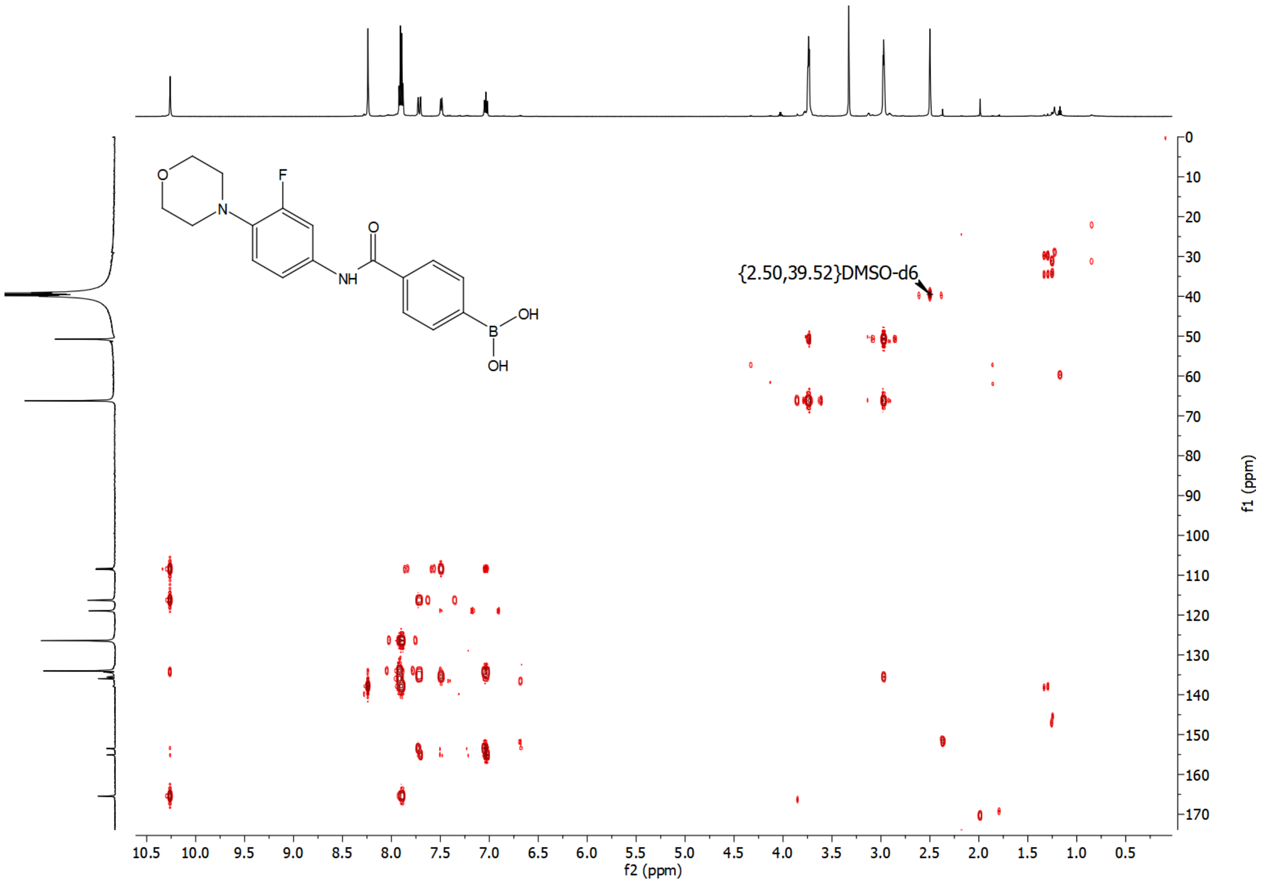

Figure S78: HMBC-NMR Spectrum of compound **39** (600 MHz DMSO-d_6).

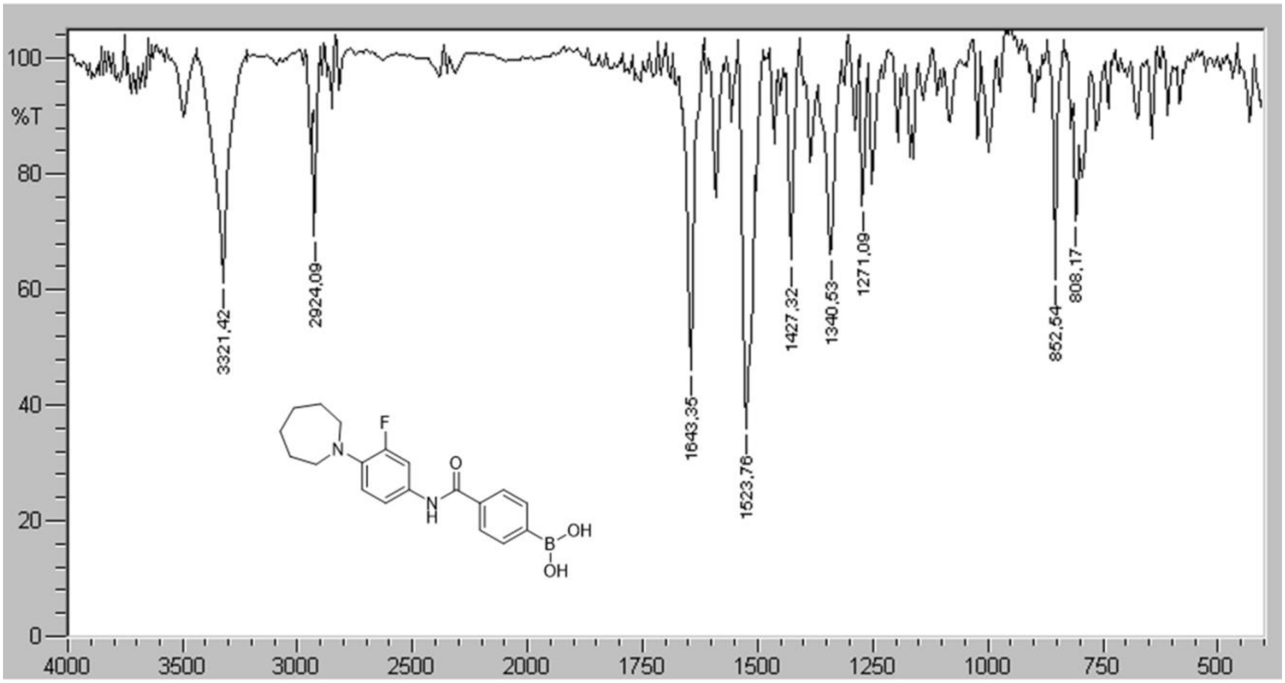

Figure S79: IR Spectrum of compound **40**.

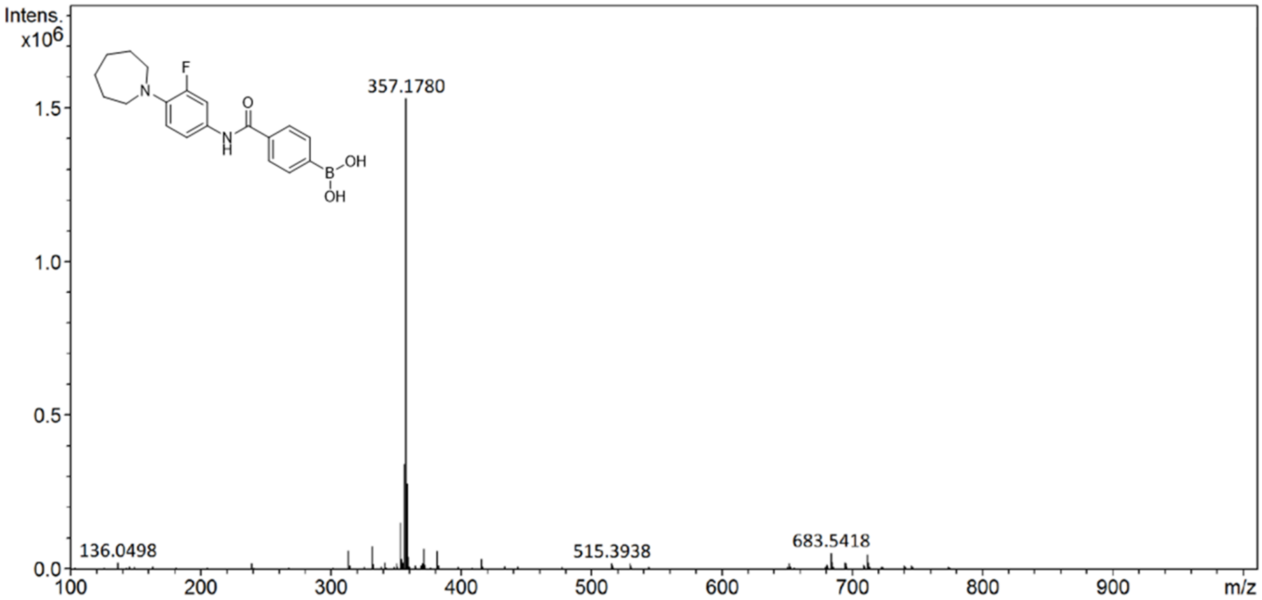

Figure S80: HRMS Spectrum of compound **40** (ESI^{+}).

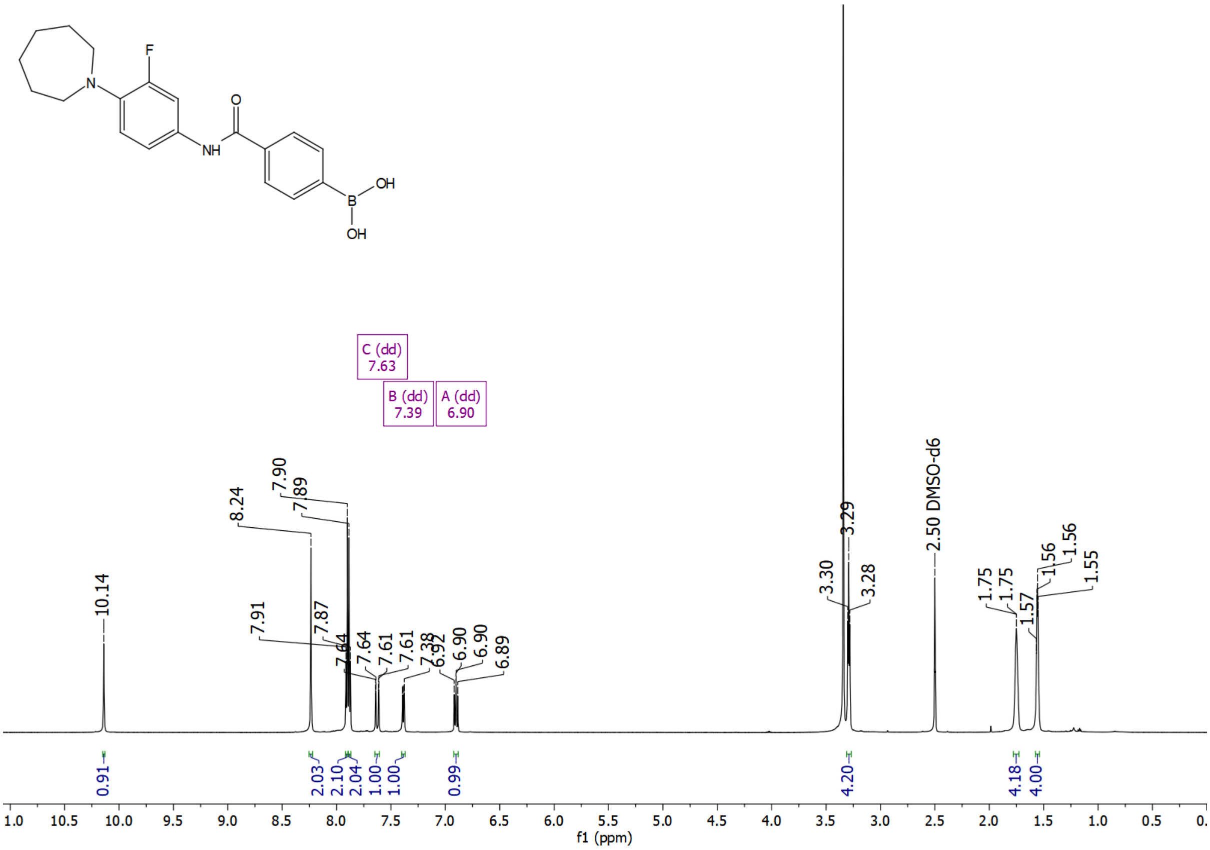

Figure S81: ^{1}H-NMR Spectrum of compound **40** (600 MHz DMSO-*d*$_6$).

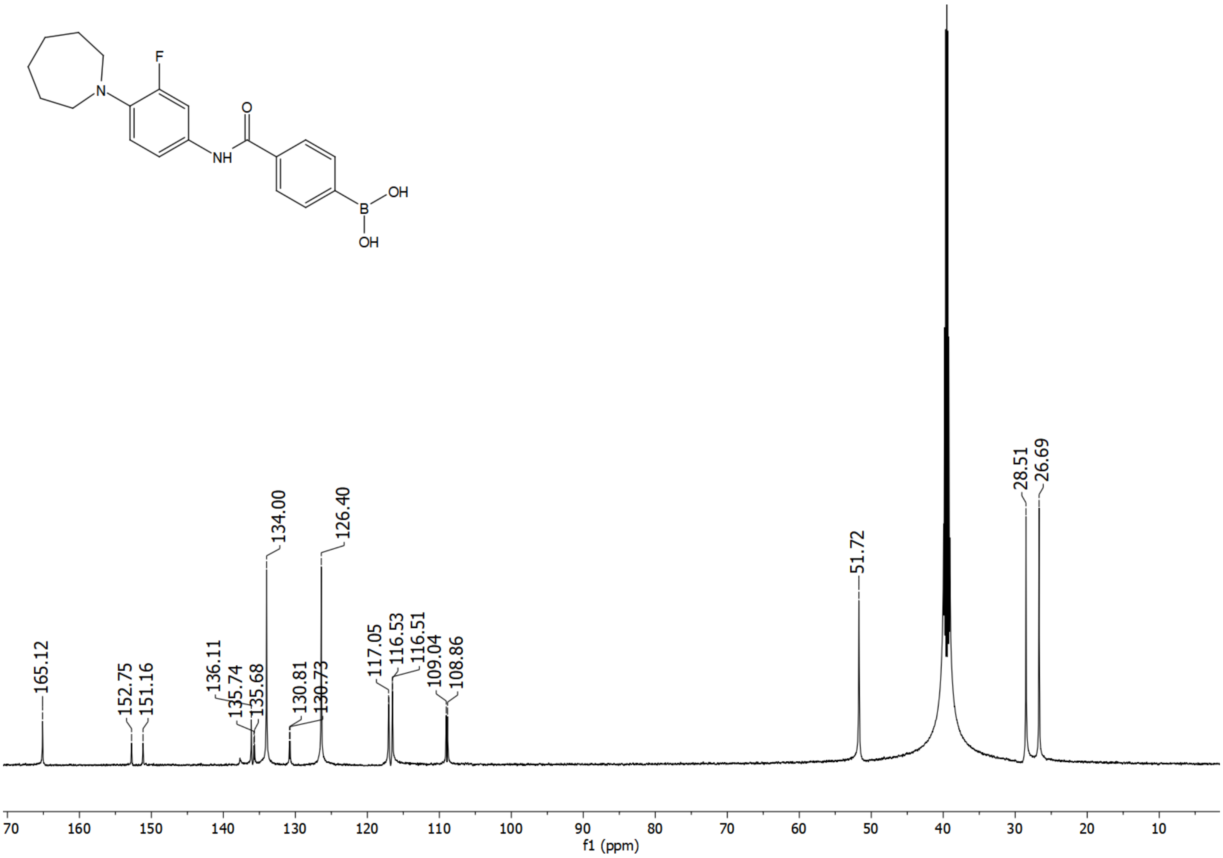

Figure S82: ^{13}C-NMR Spectrum of compound **40** (150 MHz DMSO-*d*$_6$).

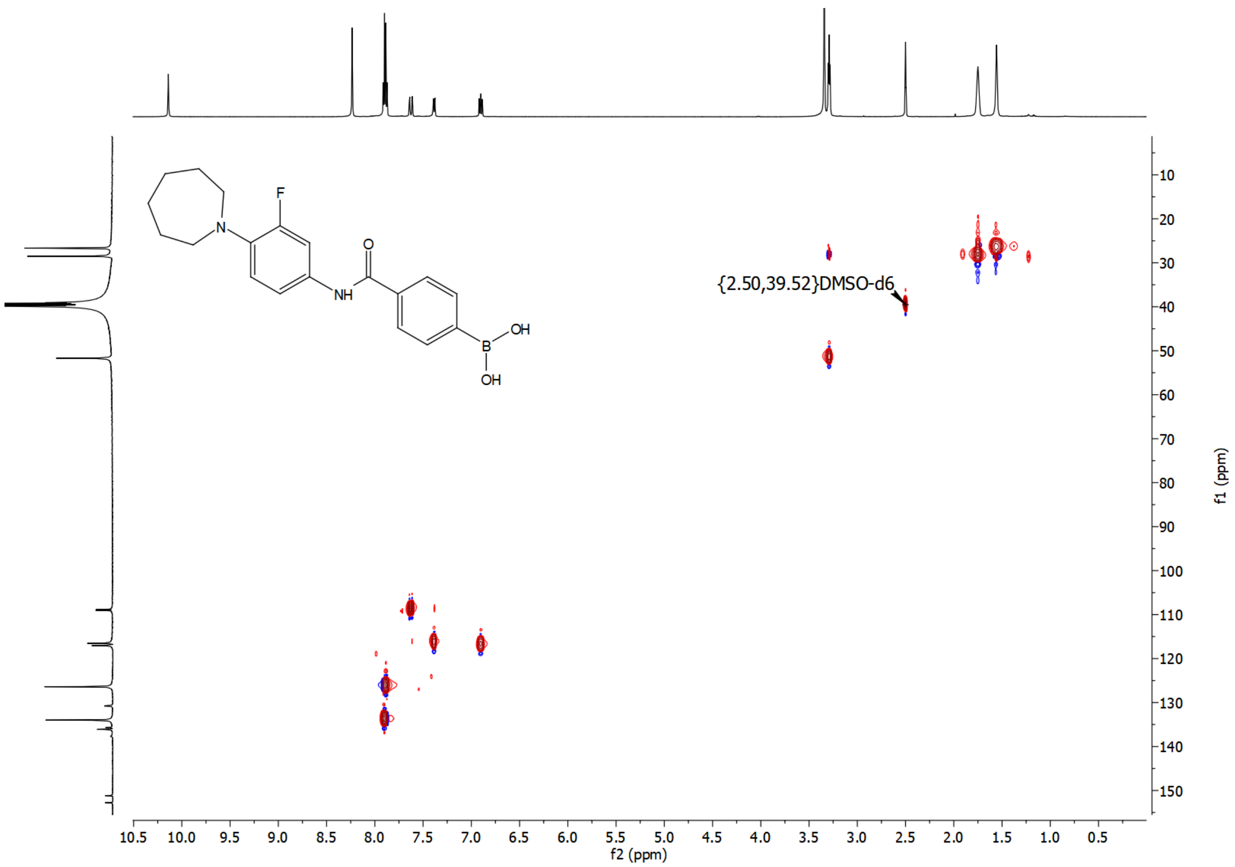

Figure S83: HSQC-NMR Spectrum of compound **40** (600 MHz DMSO-*d*6).

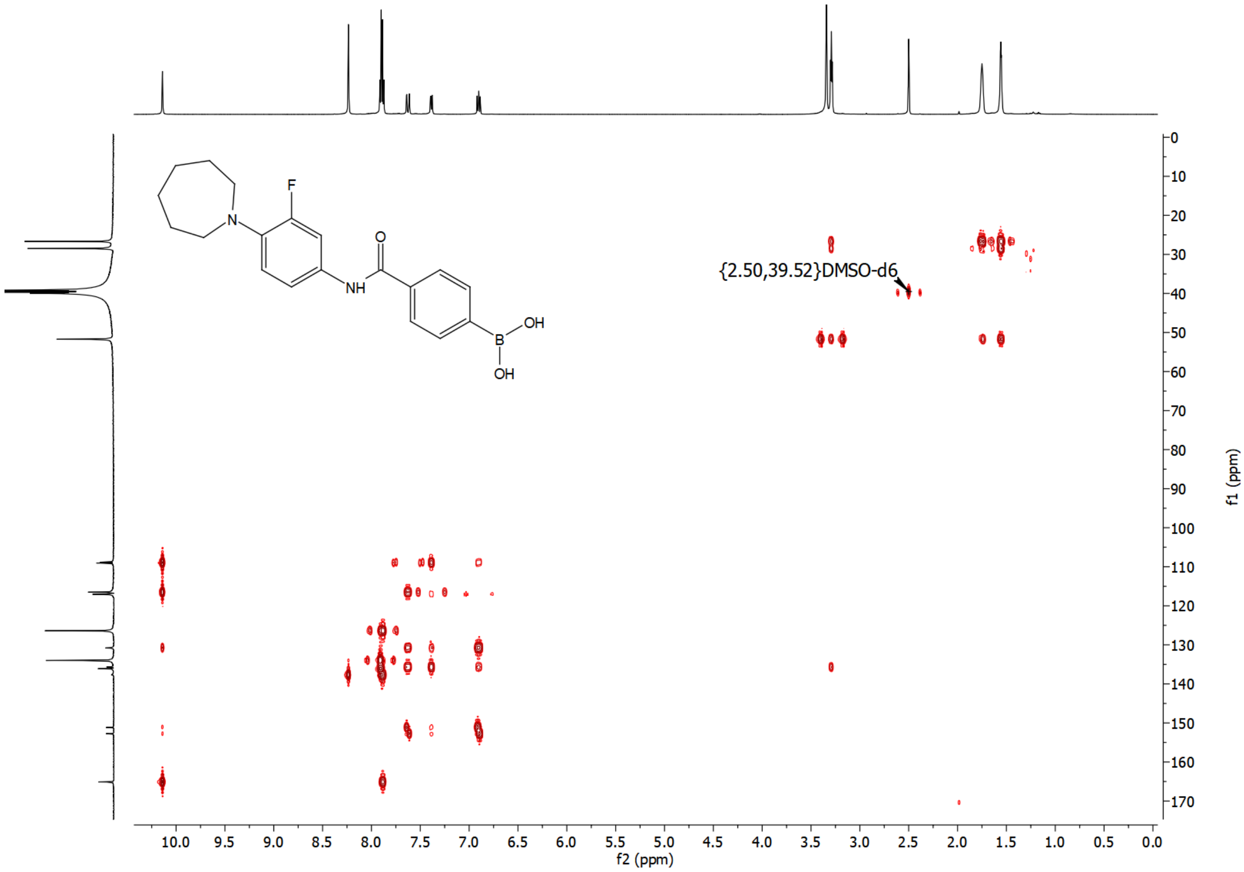

Figure S84: HMBC-NMR Spectrum of compound **40** (600 MHz DMSO-*d*6).

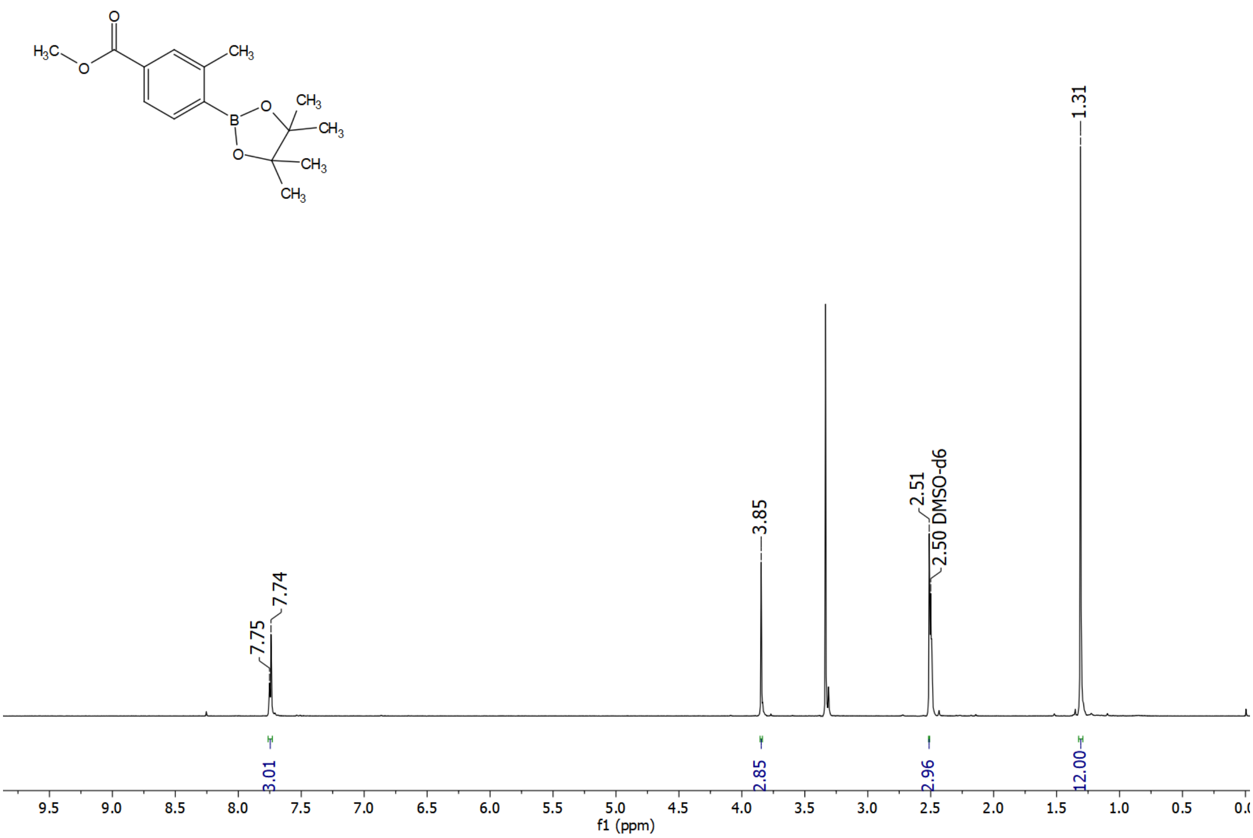

Figure S85: ^{1}H-NMR Spectrum of compound **41** (300 MHz DMSO-d_6).

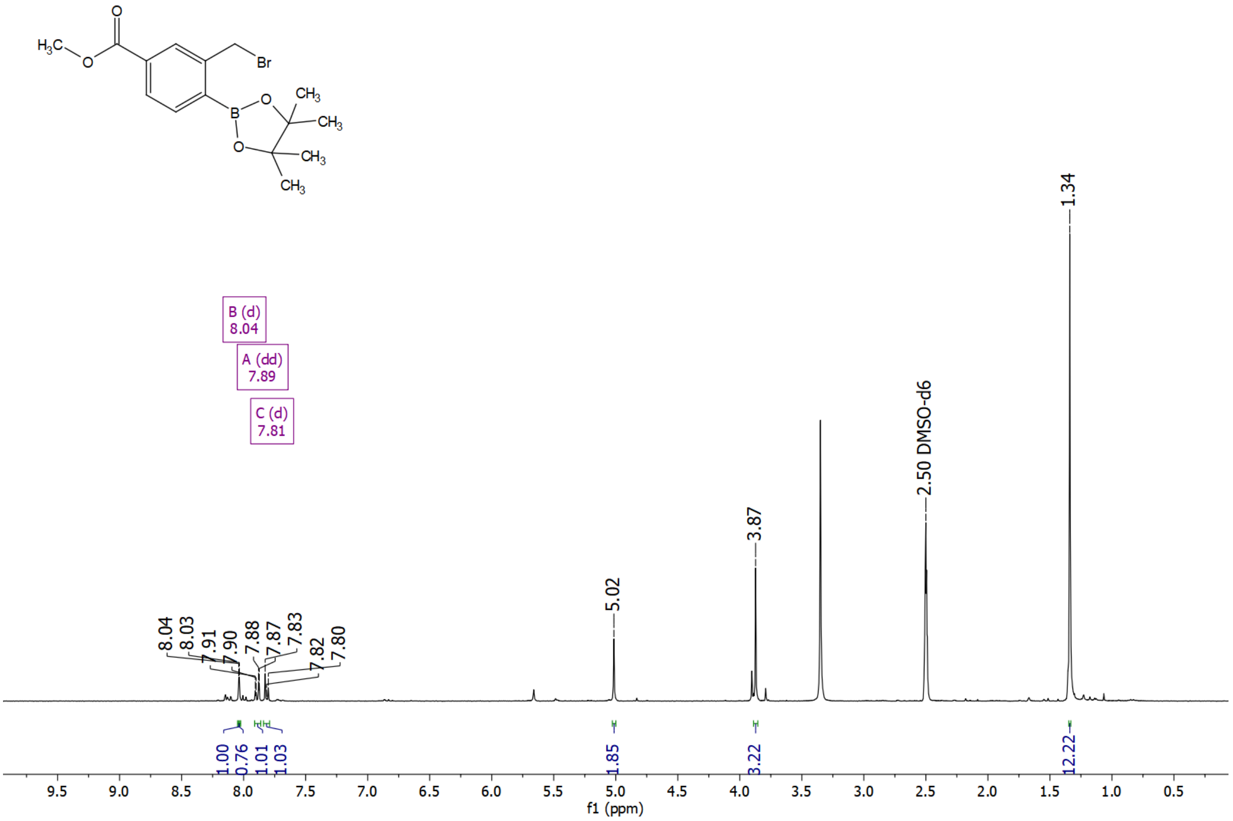

Figure S86: ^{1}H-NMR Spectrum of compound **42** (300 MHz DMSO-d_6).

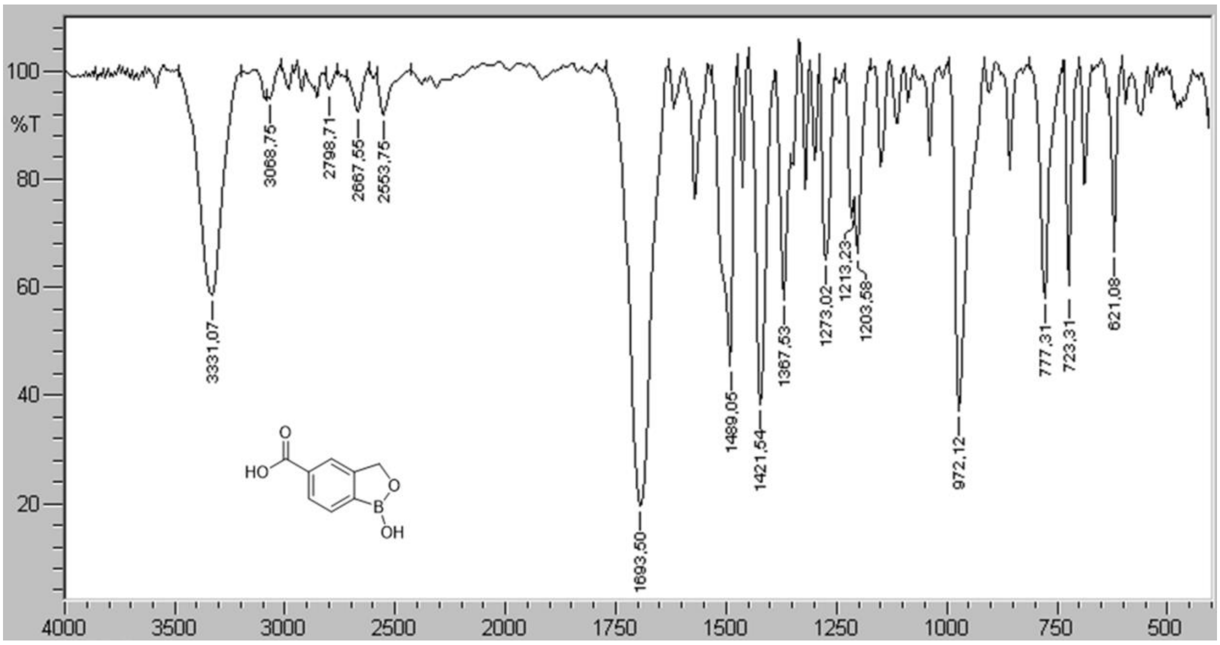

Figure S87: IR Spectrum of compound **43**.

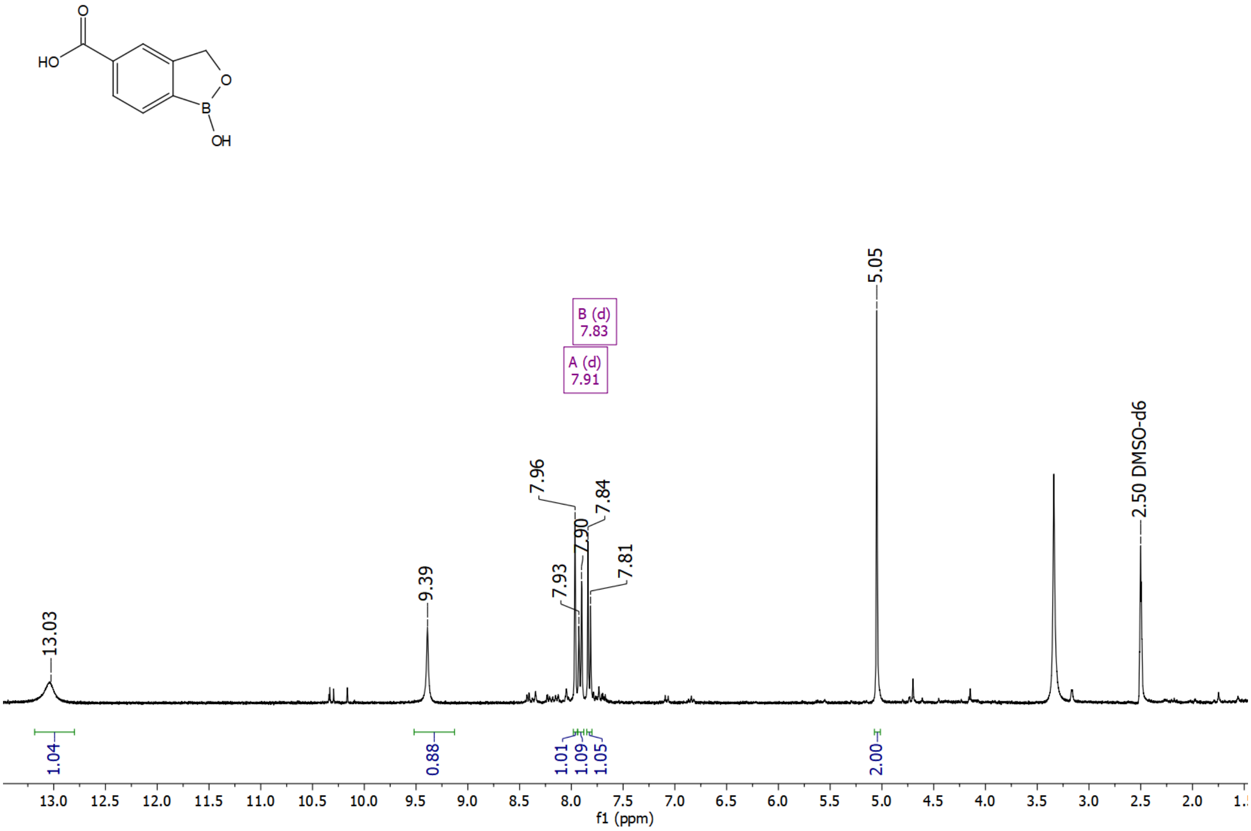

Figure S88: ^{1}H-NMR Spectrum of compound **43** (300 MHz DMSO-d_6).

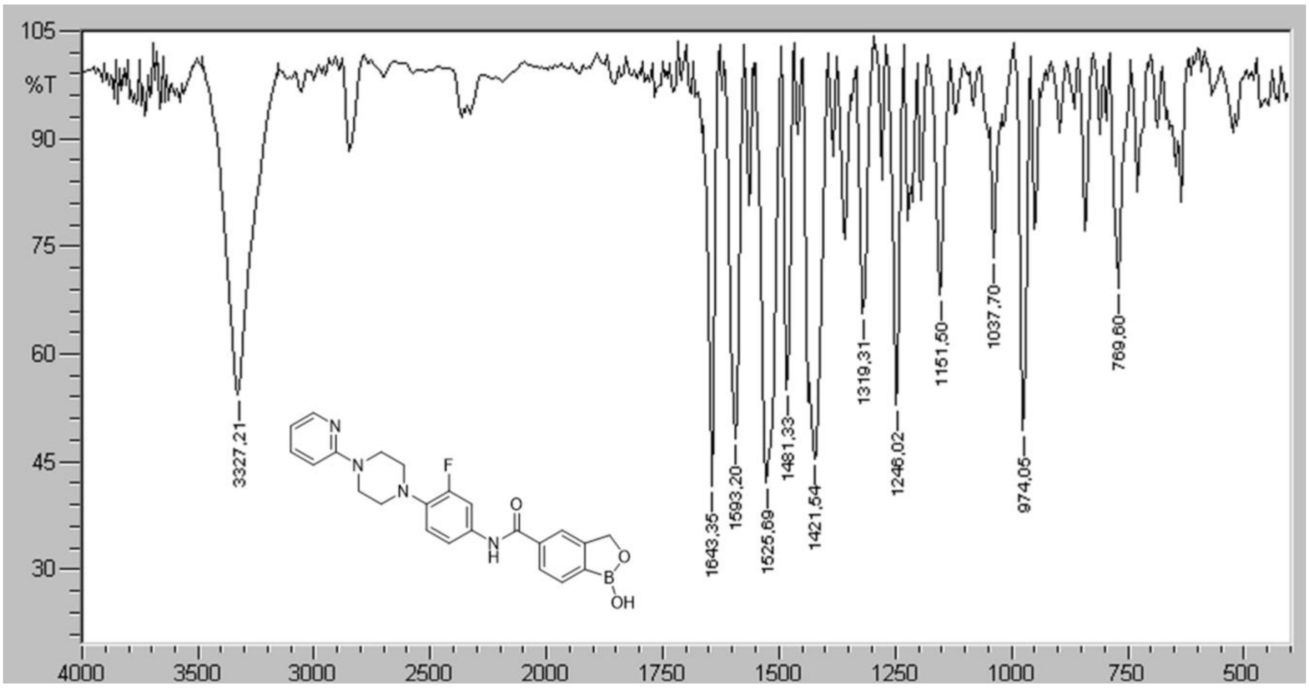

Figure S89: IR Spectrum of compound **44**.

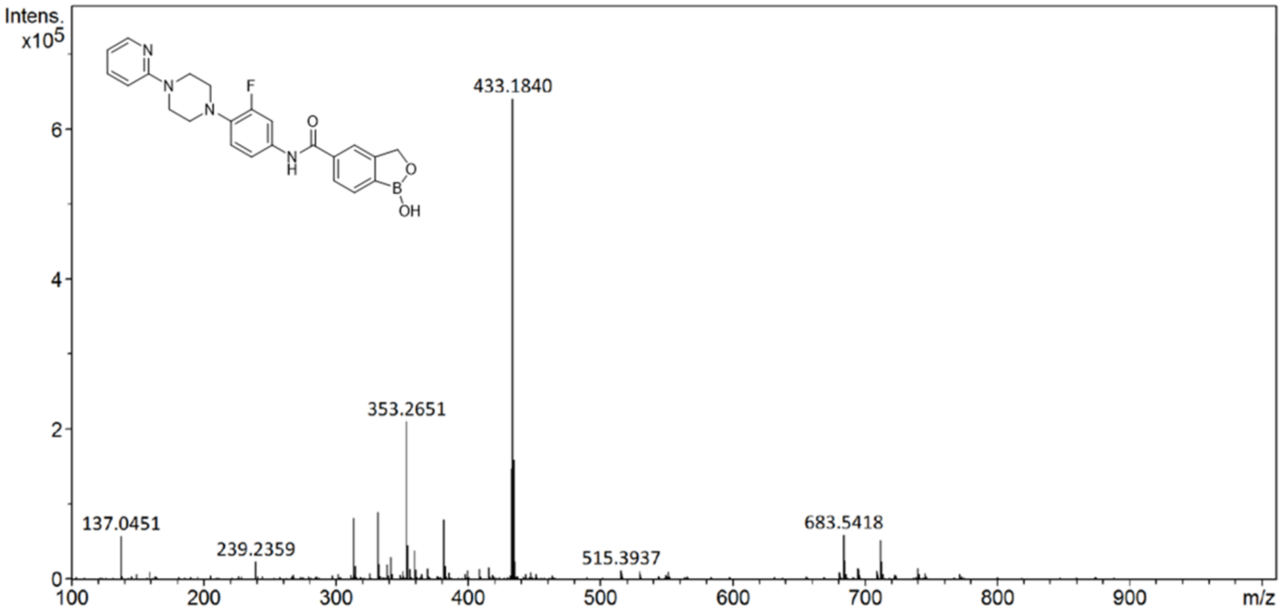

Figure S90: HRMS Spectrum of compound **44** (ESI^+).

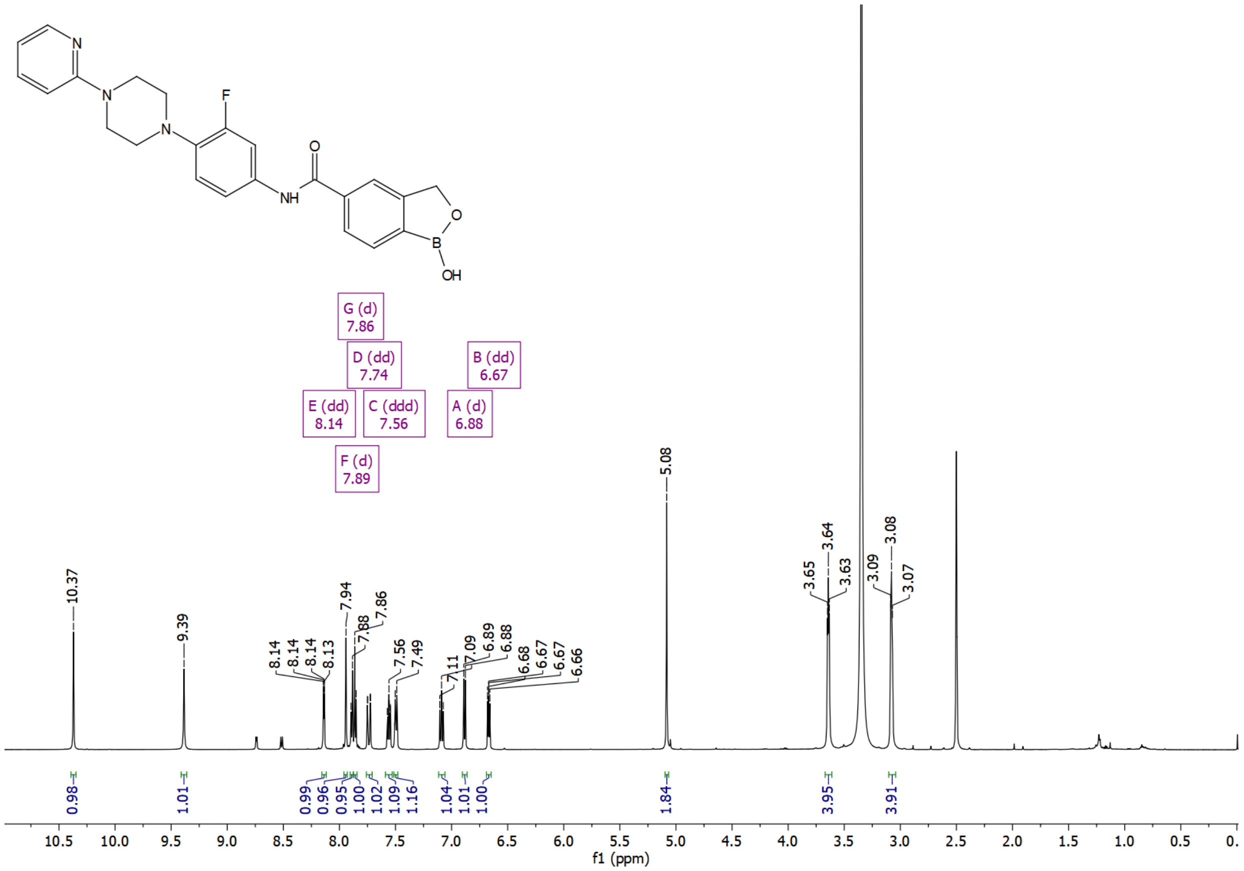

Figure S91: ^{1}H-NMR Spectrum of compound **44** (600 MHz DMSO-d_6).

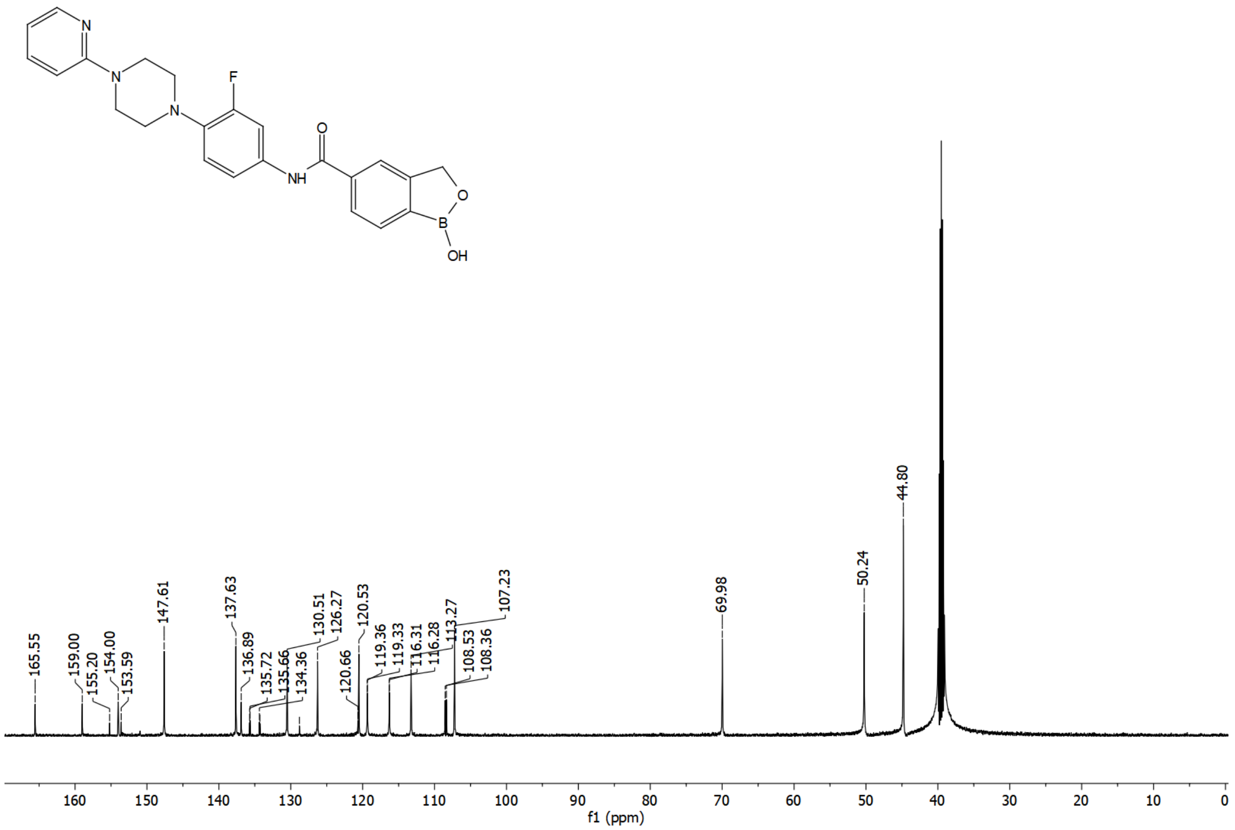

Figure S92: ^{13}C-NMR Spectrum of compound **44** (150 MHz DMSO-d_6).

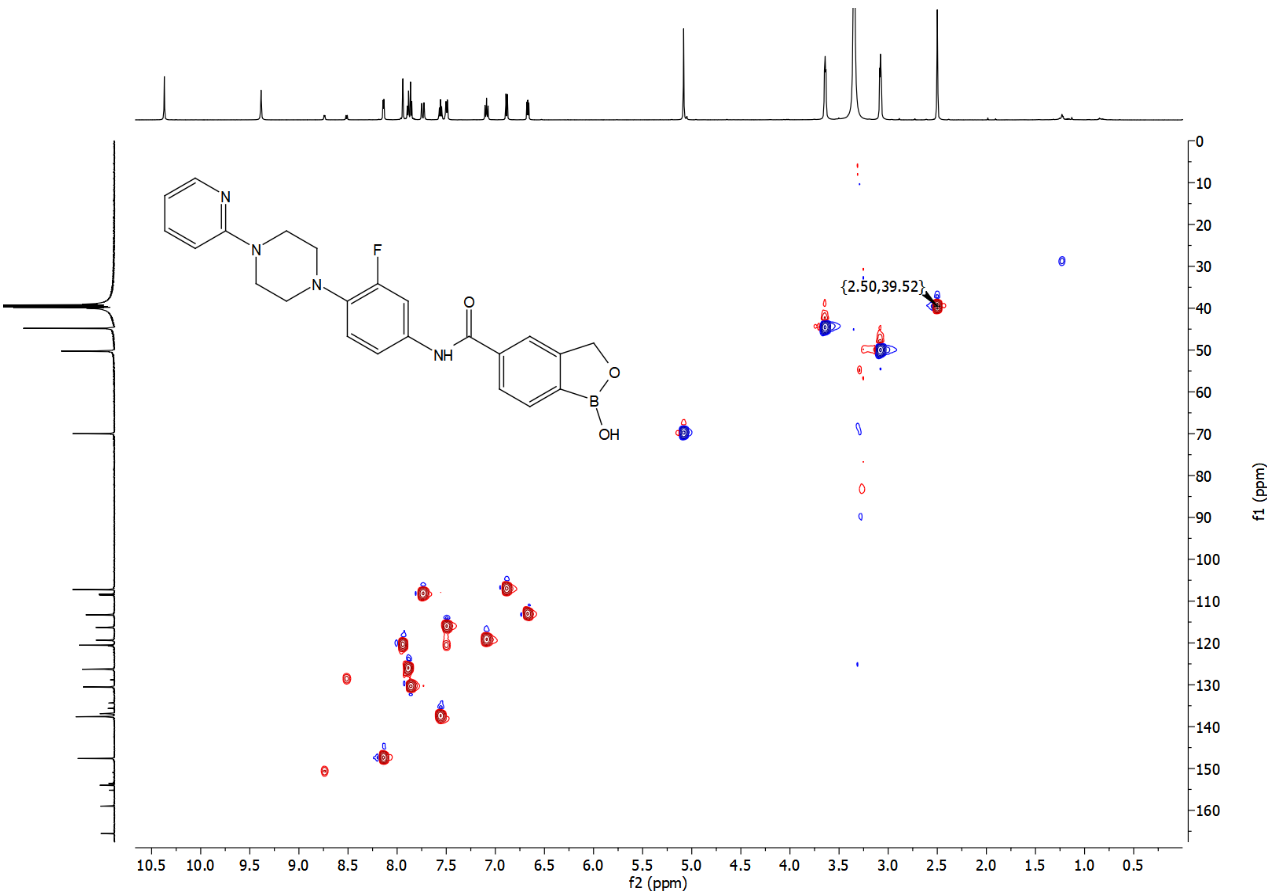

Figure S93: HSQC-NMR Spectrum of compound **44** (600 MHz DMSO-d_6).

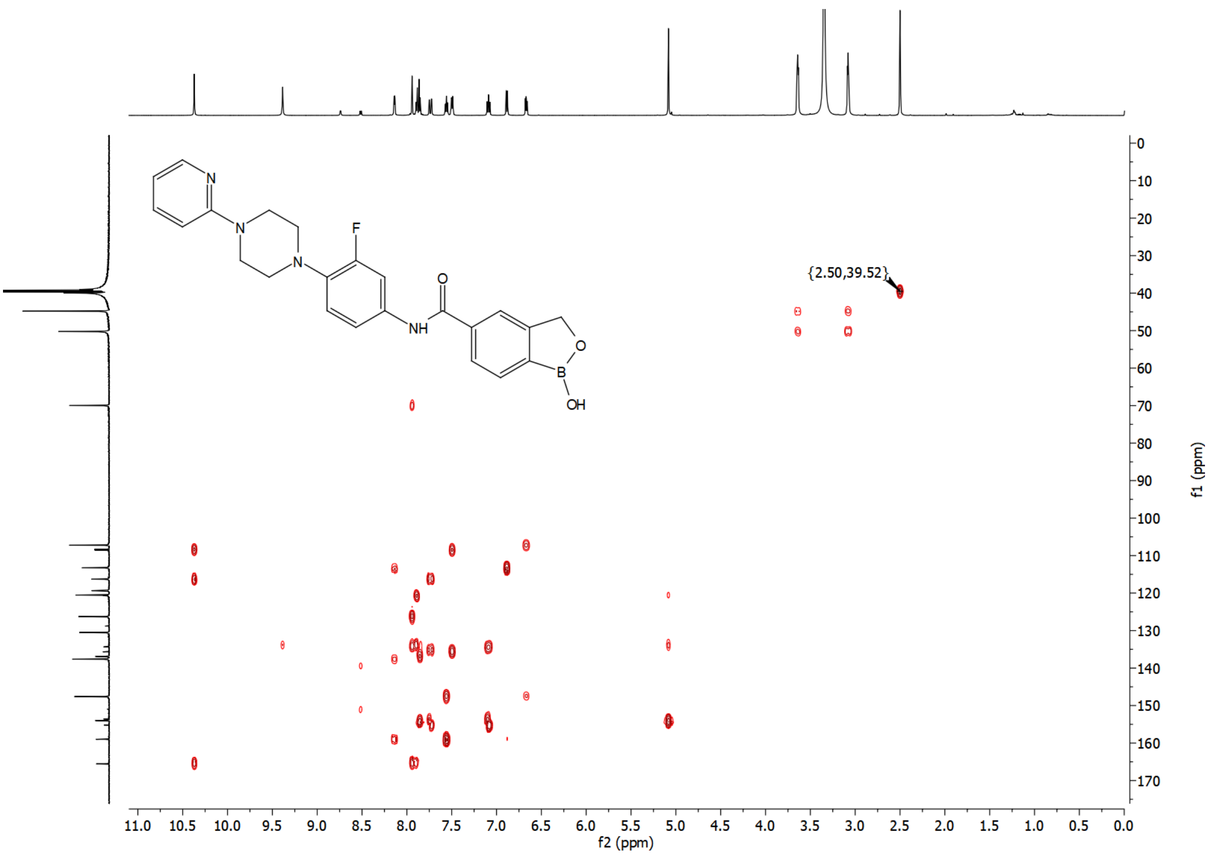

Figure S94: HMBC-NMR Spectrum of compound **44** (600 MHz DMSO-d_6).

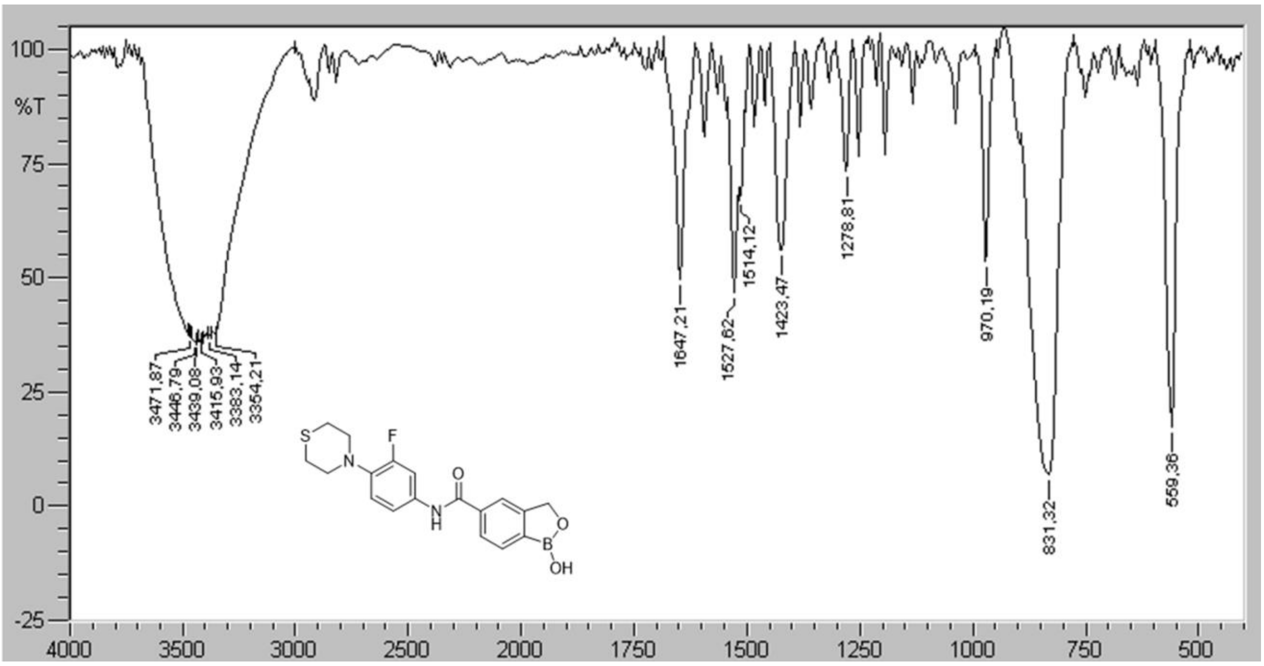

Figure S95: IR Spectrum of compound **45**.

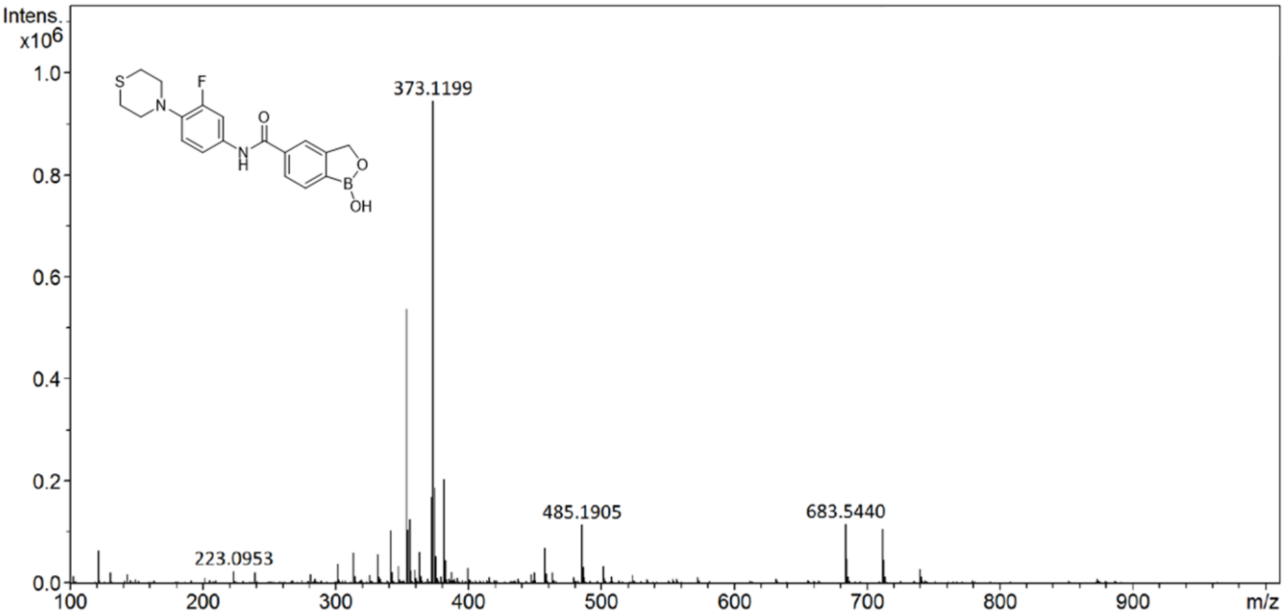

Figure S96: HRMS Spectrum of compound **45** (ESI+).

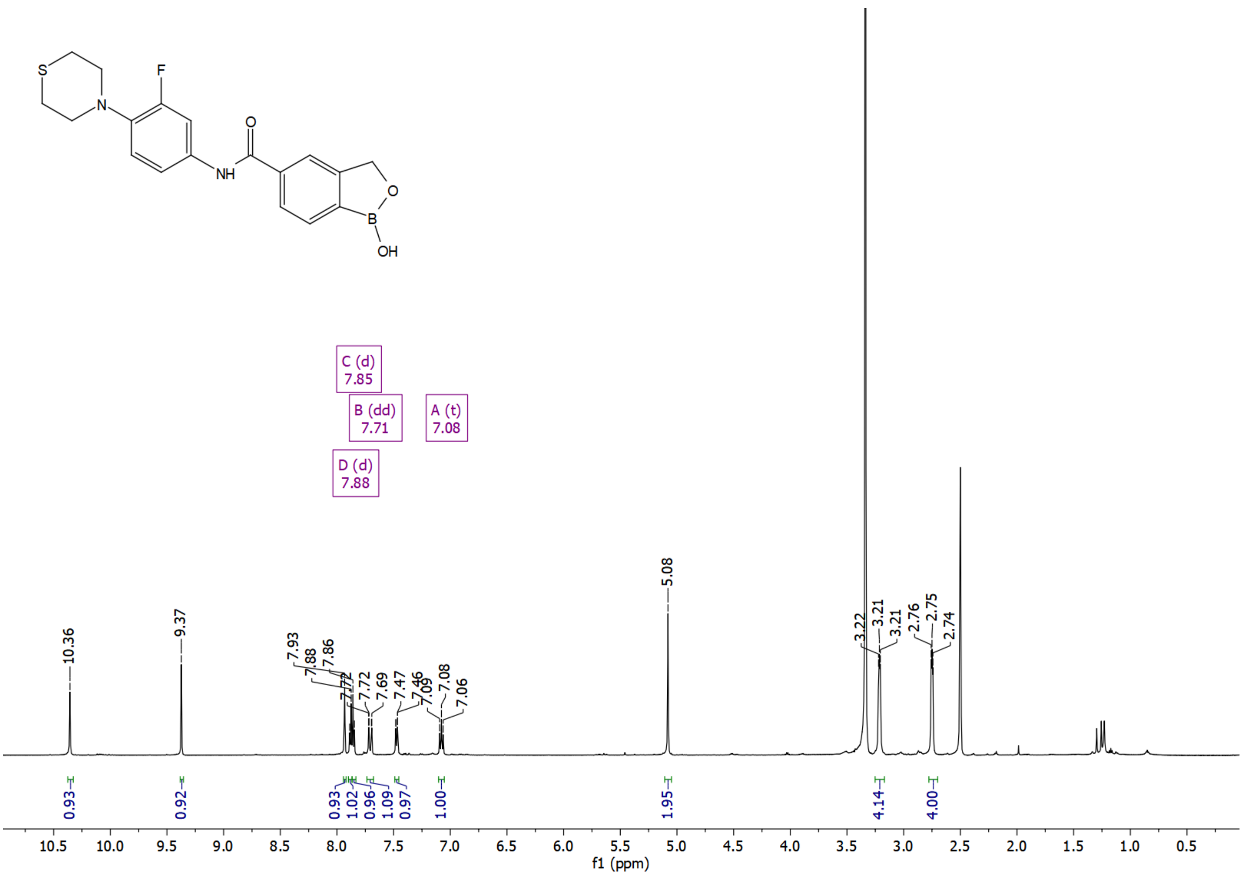

Figure S97: ^{1}H-NMR Spectrum of compound **45** (600 MHz DMSO-d_6).

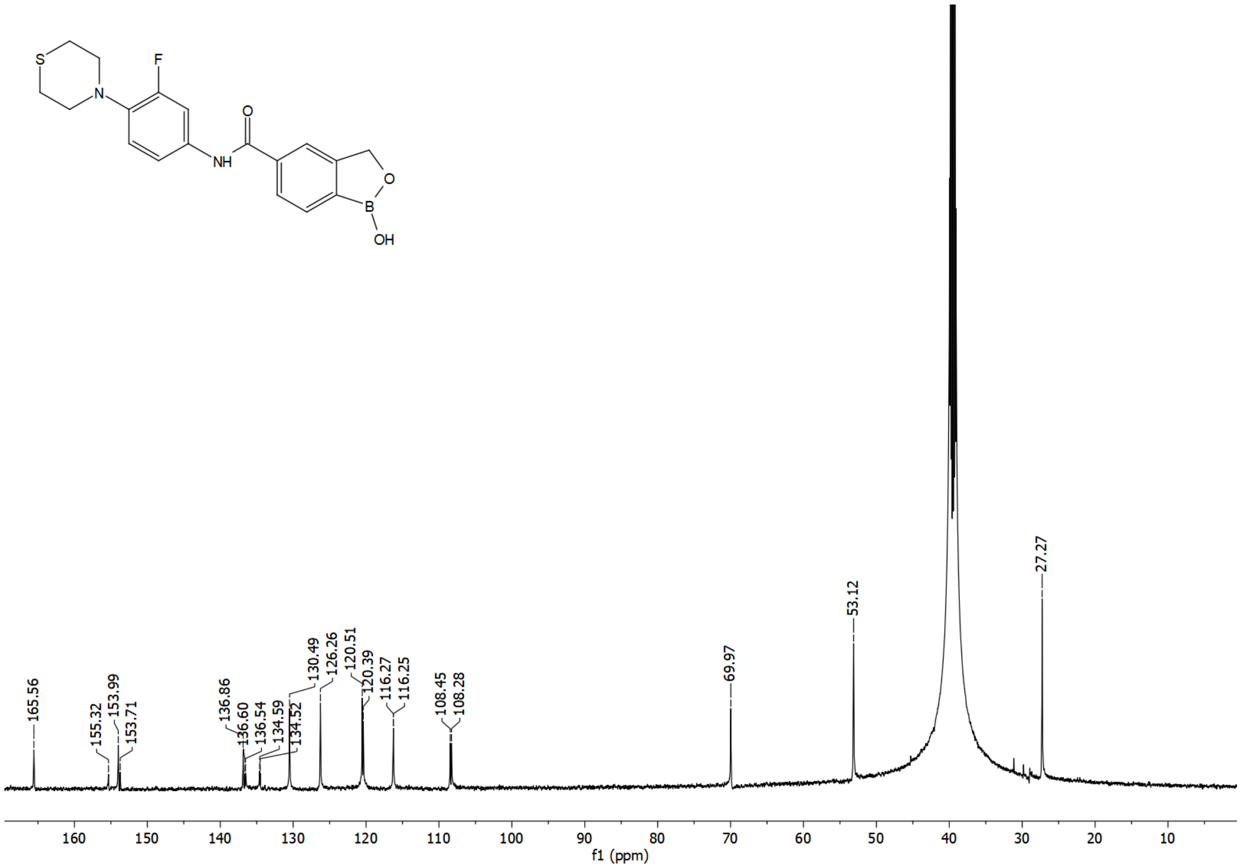

Figure S98: ^{13}C-NMR Spectrum of compound **45** (150 MHz DMSO-d_6).

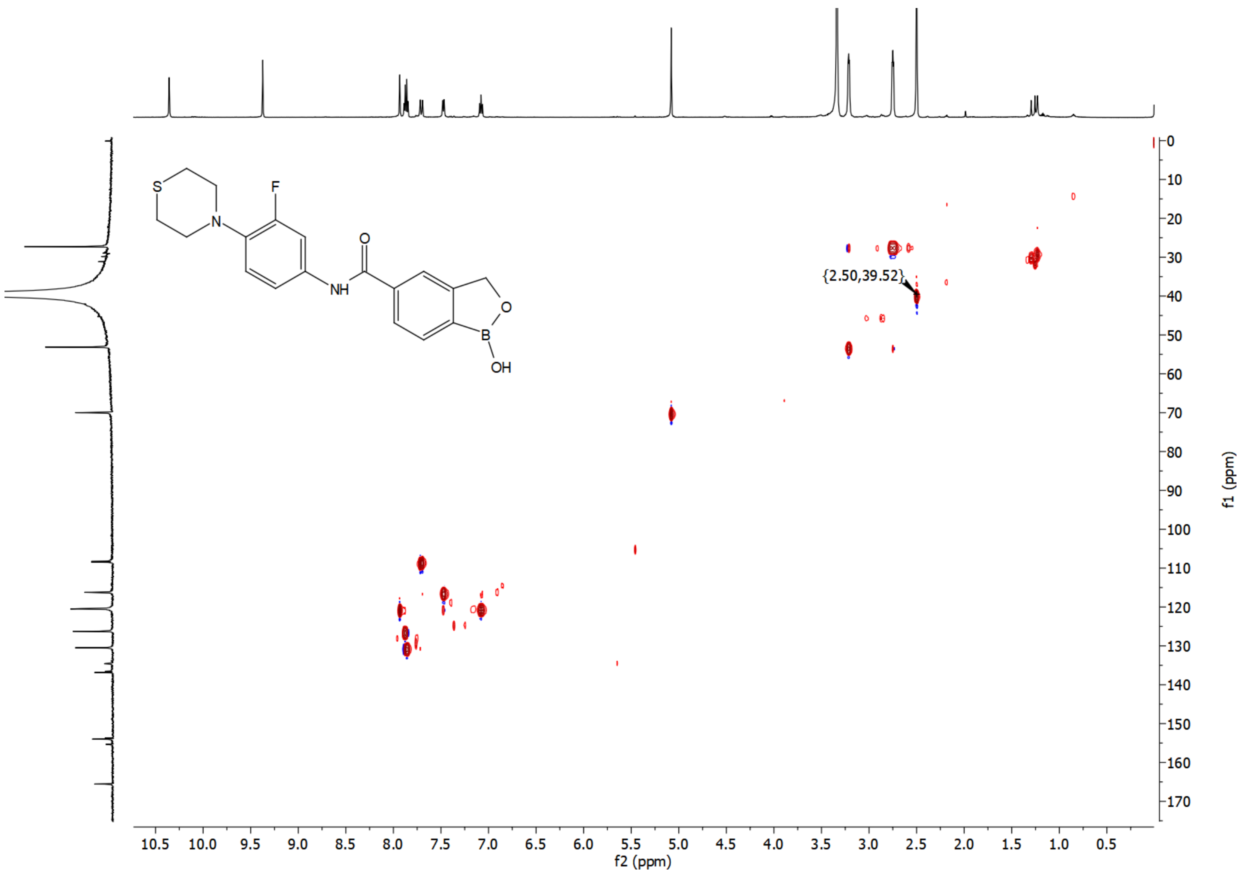

Figure S99: HSQC-NMR Spectrum of compound **45** (600 MHz DMSO-d_6).

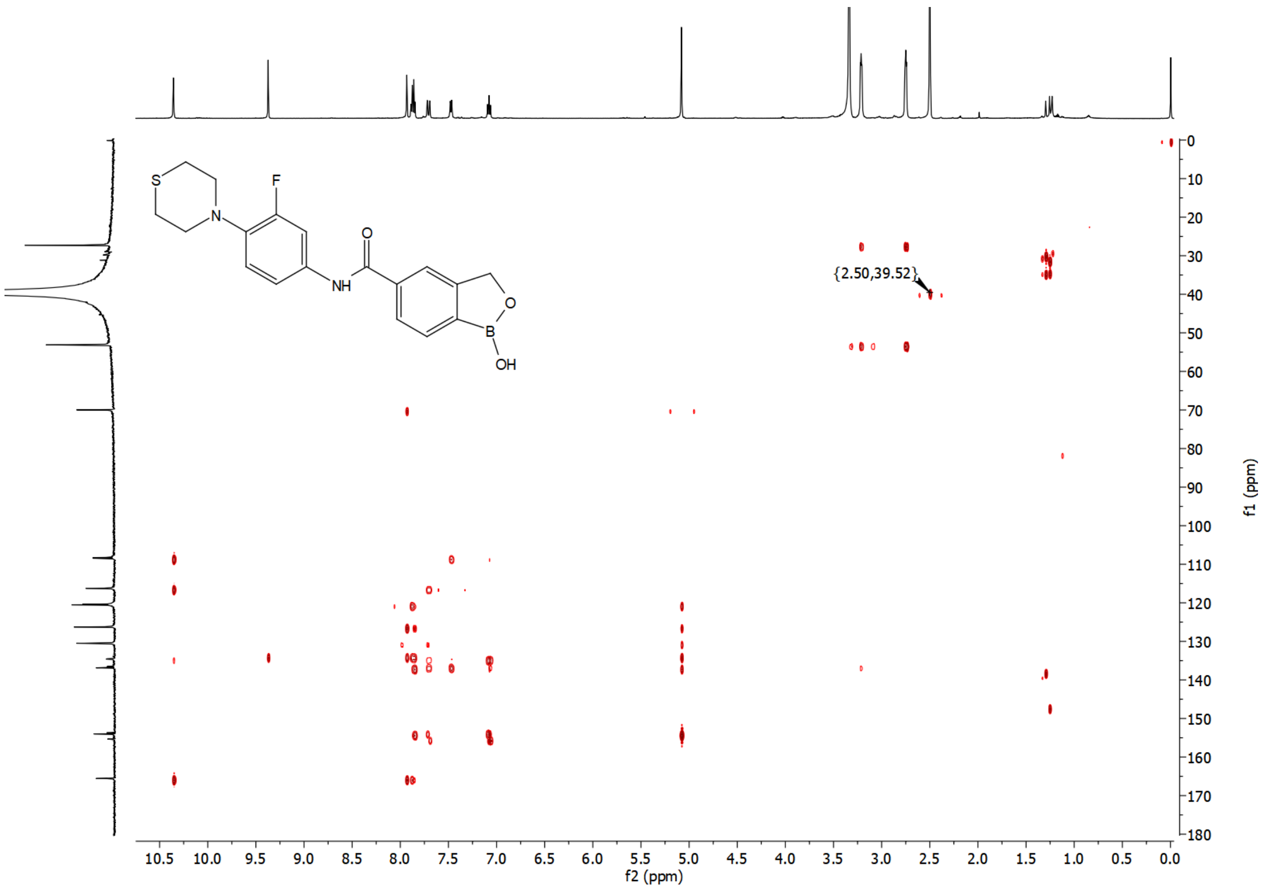

Figure S100: HMBC-NMR Spectrum of compound **45** (600 MHz DMSO-d_6).
